# Using Electroencephalogram-Extracted Nonlinear Complexity and Wavelet-Extracted Power Rhythm Features during the Performance of Demanding Cognitive Tasks (Aristotle’s Syllogisms) in Optimally Classifying Patients with Anorexia Nervosa

**DOI:** 10.3390/brainsci14030251

**Published:** 2024-03-04

**Authors:** Anna Karavia, Anastasia Papaioannou, Ioannis Michopoulos, Panos C. Papageorgiou, George Papaioannou, Fragiskos Gonidakis, Charalabos C. Papageorgiou

**Affiliations:** 1 Eating Disorder Unit, 2nd Department of Psychiatry, Medical School, National & Kapodistrian University of Athens, ‘Attikon’ University Hospital, 1 Rimini St., 12462 Athens, Greece; imihopou@med.uoa.gr; 2 1st Department of Psychiatry, Medical School, National & Kapodistrian University of Athens, Eginition Hospital, 74 Vas. Sofias Ave., 11528 Athens, Greece; anas.papaioannou24@gmail.com (A.P.); fragoni@yahoo.com (F.G.); chpapag@med.uoa.gr (C.C.P.); 3 Neurosciences and Precision Medicine Research Institute “COSTAS STEFANIS” (UMHRI), University Mental Health, Soranou tou Efesiou 2, Papagou, 11527 Athens, Greece; 4 Department of Electrical and Computer Engineering, University of Patras, 26504 Rion-Patras, Greece; papageorgiou@ece.upatras.gr; 5 Center for Research of Nonlinear Systems (CRANS), Department of Mathematics, University of Patras, 26500 Rion-Patras, Greece; gpthespies@gmail.com

**Keywords:** approximate entropy, EEG features, machine learning classifiers, anorexia nervosa, Aristotelian syllogisms, information processing, reasoning tasks

## Abstract

Anorexia nervosa is associated with impaired cognitive flexibility and central coherence, i.e., the ability to provide an overview of complex information. Therefore, the aim of the present study was to evaluate EEG features elicited from patients with anorexia nervosa and healthy controls during mental tasks (valid and invalid Aristotelian syllogisms and paradoxes). Particularly, we examined the combination of the most significant syllogisms with selected features (relative power of the time–frequency domain and wavelet-estimated EEG-specific waves, Higuchi fractal dimension (HFD), and information-oriented approximate entropy (AppEn)). We found that alpha, beta, gamma, theta waves, and AppEn are the most suitable measures, which, when combined with specific syllogisms, form a powerful tool for efficiently classifying healthy subjects and patients with AN. We assessed the performance of triadic combinations of “feature–classifier–syllogism” via machine learning techniques in correctly classifying new subjects in these two groups. The following triads attain the best classifications: (a) “AppEn-invalid-ensemble BT classifier” (accuracy 83.3%), (b) “Higuchi FD-valid-linear discriminant” (accuracy 75%), (c) “alpha amplitude-valid-SVM” (accuracy 83.3%), (d) “alpha RP-paradox-ensemble BT” (accuracy 85%), (e) “beta RP-valid-ensemble” (accuracy 85%), (f) “gamma RP-valid-SVM” (accuracy 85%), and (g) “theta RP-valid-KNN” (accuracy 80%). Our findings suggest that anorexia nervosa has a specific information-processing style across reasoning tasks in the brain as measured via EEG activity. Our findings also contribute to further supporting the view that entropy-oriented, i.e., information-based features (the AppEn measure used in this study) are promising diagnostic tools (biomarkers) in clinical applications related to medical classification problems. Furthermore, the main EEG-specific frequency waves are extremely enhanced and become powerful classification tools when combined with Aristotle’s syllogisms.

## 1. Introduction

### 1.1. The Main Aim of the Work

Reasoning refers to the cognitive process of reaching a conclusion even when the available information is incomplete [1]. Inductive reasoning includes the introduction of predictions about novel situations or events based on experience and pre-existing knowledge concerning the probability that a certain event occurs [2]. On the other hand, deductive reasoning is considered a hallmark of higher cognition since it involves drawing specific conclusions based on premises [1]. Theoretically, it should be expected that this type of reasoning depends on logical forms only, but research shows that reasoners become influenced by the determinate content of the premises [3].

The process of reasoning is not based entirely on formal considerations; it is affected by content or belief bias effects [4]. Researchers are interested in investigating the underlying mechanisms of inductive and deductive reasoning and whether these mechanisms are common or separate [2].

Despite the fact that the process of reasoning does not lack biases, the relevant cognitive mechanisms remain to be clarified. There is supporting evidence that when people are asked to make decisions themselves or evaluate decisions made by others, they are aware of their biased reasoning, as shown by a decreased level of confidence in their answers to cognitive tasks [5].

Considering all the above, a fundamental query that arises is whether psychopathology and/or personality traits are involved in the reasoning process and may impact syllogistic performance [6]. In this context, there is growing interest as well as a variety of existing data concerning different aspects of cognitive function in anorexia nervosa; however, to our knowledge, the process of reasoning itself has not been explored in relation to possible distinct brain activation patterns with the concomitant use of EEG recordings in this population when facing reasoning tasks.

Increased diagnostic accuracy and a deeper mechanistic understanding of psychopathology can be attained using nonlinear features extracted from EEG analyses [7]. The main aim of the present work is to compare different descriptive features extracted from EEG recordings using a variety of linear and nonlinear complexity measures in the context of a classification problem. More specifically, EEG recordings from healthy subjects (controls) and patients with anorexia nervosa (AN), a type of eating disorder (ED), reflect the performance of the above subjects in their effort to “manage” the induced cognitive loads from their exposure to cognitive tasks of varying difficulty, associated with the “peculiarities” of Aristotelian valid, invalid, and paradoxical syllogisms, and optical illusions. The extracted (EEG) features based on the estimated values of AppEn, HFD, and the relative power of the alpha, beta, delta, and theta (wavelet-extracted) rhythms are then tested for their discriminating capacity, using ANOVA and nonparametric tests. The best features found are then used as input to a classification problem where the target feature is the “group” factor (variable) that assigns subjects to the following two classes: control group or AN group. Therefore, finding the optimum triad “feature–syllogism–classifier” becomes the most crucial step in the process of detecting which feature works better with a given syllogism and classifier for effectively classifying subjects in the correct groups of controls and patients with AN.

The possible variability and differences in the average response values of the subjects of the two groups when exposed to such mentally demanding tasks need to be examined in association with possible neuroanatomical differences between the subjects. Therefore, this consideration requires us to explore the involvement or coupling of the following factors: (a) the reasoning strategies used by these groups in handling such demanding mental tasks; (b) Aristotle’s types of syllogisms; (c) the complexity and variability of EEG signals of healthy and “pathological” subjects, as the aforementioned features quantify them; and finally (d) the primary cognitive processes (cognitive control, attention, working memory, etc.) that are present during the tasks and more importantly how their associated changes, as “captured” by the EEGs at a number of brain regions (channels), are reflected via the extracted features as, e.g., the AppEn and the relative power of alpha, theta, beta, delta, and gamma rhythms. 

The interplay between these concepts will provide useful information for building, as accurately as possible, classification models (or classifiers) in successfully assigning new subjects to the two groups. The clinical benefits of such powerful classifiers are, therefore, profound. 

### 1.2. The Structure of Aristotle’s Syllogism and Its Relation to Cognitive Processes—Reasoning Starts with Premises, Which Can Be Statements, Perceptions, or Beliefs

At this point, we briefly describe the syllogistic reasoning task and Aristotelian classification that explain the cognitive loads the Aristotelian syllogisms impose on subjects when they try to “handle” them during experiments. Syllogisms are constructed with two premises and one conclusion. A characteristic illustration of reasoning is presented by the following example:

*All men are mortal*

*Socrates is a man*

*(then) Socrates is mortal*

Reasoning is intended to derive reasonable conclusions from premises and is carried out in working memory. Human performance on syllogistic reasoning is based on mental representations, as the mental model theory (one of the earliest comprehensive psychological theories of reasoning) postulates [8]. As the authors in this study assume, the *difficulty of syllogistic reasoning is a function of the number of mental models that must be constructed to derive a logically valid conclusion.* In addition, a task that requires more models to be constructed to lead to a correct answer increases the probability of errors in the inference process, possibly resulting in failure. As the mental model theory claims, the difficulty of (or cognitive load exerted in) syllogisms is determined mainly by the number of models and the Aristotelian *figure* (one of the four likely possibilities described in Section 2.3). The mental model theory of reasoning incorporates the working memory capacity, an important component of the cognitive process [9]. According to the *Sample Mental Model (SMM),* a probabilistic approach in a mental representation [10], *people “adopt” or sample six or seven instances in working memory to derive a conclusion to a syllogism.* Nevertheless, according to Halford et al. [11], the limit of working memory capacity is three to five chunks (“fat pieces”) reflecting a human’s capacity for attention and constraints the relational representations, enabling inference making.

All the preceding information leads to the conclusion that the structure of Aristotelian syllogisms, reflected by *modes* and *figures*, in combination with the difficulty of syllogistic reasoning (determined by the number of mental models needed to be constructed before reaching a valid conclusion) and the limited capacity of working memory (that forces the ability to work inferences), may be considered as the sources of cognitive loads exerted on subjects exposed to Aristotelian syllogisms, responsible for shaping the dynamics of the EEGs recorded during relevant experiments. The sources of cognitive loads may be located at and activated by different brain regions in different ways, depending on task conditions or the subject’s “pathological” condition. It is challenging to investigate how healthy (controls) and AN subjects “behave” when facing Aristotelian syllogisms since the loads exerted on subjects of the two groups differ as described above. Aristotle’s deduction method is probably the first to analyze logical reasoning or syllogism (also called valid reasoning). In his famous work “ORGANON–Prior analytics” [12,13], a series of statements (the “building blocks”) is presented in the process of reasoning that leads to a *valid conclusion with absolute certainty*. The dual processing model for logical reasoning [14,15,16] is developed to help understand how the brain functions when a subject is “engaged” in this type of reasoning. A natural question that is generated is whether Aristotle’s syllogisms activate the same or different mental processes in the brain. This is a current, still open, and challenging question intending to shed light on the fundamental operation of reasoning in its two extreme conditions. 

In this work, the center of our attention is an experiment called “*Aristotle’s experiment*”, in which EEG signals of subjects from two groups (control, AN) are recorded while exposed to the valid, invalid, and paradoxical types of reasoning. During the experiment, each participant’s working memory (WM) was induced, as it is a critical cognitive activity to retain information “alive” not just for memorizing purposes but also for other necessary cognitive tasks like reasoning, problem-solving, decision-making, planning, etc.

One of the main objectives of this work is to discover whether different modes of electro-physiological activity are activated when healthy subjects (controls) and patients with AN (ED) are exposed to *Aristotle’s syllogisms*. The contribution of this study is that it sheds light on how critical aspects of the reasoning process related to attention, perception, and cognitive behavior differ between the groups of participants. The difference in EEG signals in healthy participants exposed to valid syllogisms and paradoxes has been analyzed before in the work of Papageorgiou et al. [1,17] and Papaodysseus et al. [18]. Recently, an fMRI study by Belekou et al. [19] provided further evidence that valid and paradoxical reasoning engages distinct brain activation patterns. Finally, the work of Papaioannou et al. [20,21] is very relative to this study since it has similar objectives, at least with respect to the reasoning strategies of subjects when they are exposed to Aristotelian syllogisms, yet with differences in the groups of subjects analyzed (ASD and ADHD) and the EEG extracted features used. 

### 1.3. Literature Review on Related Work

#### 1.3.1. Literature Review on Aspects of Cognitive Function, Linear and Nonlinear EEG Features Extractions in AN

According to the Diagnostic and Statistical Manual of Mental Disorders, DSM-5 (5th ed.) of the American Psychiatric Association [22], *Anorexia Nervosa (AN) is a psychiatric disorder associated with severe restriction of energy intake, which results in significantly low body weight, accompanied by a strong fear of gaining weight, and a perceptional distortion of one’s body weight or appearance*. For adolescent girls and young adults, AN is widely viewed as an important etiology of high-rate physical and psychosocial morbidity. More specifically, AN is a disorder with high rates of morbidity and mortality among women (it may be up to 4%) [22], while the crude rate of mortality due to all causes of AN is estimated at 5.9% [23]. The neural foundations of the etiology of AN are not well understood, and consequently, the long-term prognosis of AN is weak, and its effective treatment is unfortunately limited. Furthermore, anorexia nervosa is associated with difficulties in different aspects of cognitive function, such as attention, processing speed, visual and verbal memory, and visuospatial construction [24,25,26,27,28].

Intense research is currently in progress, focusing on the structural and functional abnormalities of the brain in patients with AN, using a variety of tools and methods [23]. In their work, Su et al. [23] conducted a whole brain meta-analysis on voxel-based morphometry (VBM) using rsfMRI, and they found that overall, patients with AN exhibited decreased gray matter volume (GMV) in the bilateral median cingulate cortex and left middle occipital gyrus, a result that contributes to a better understanding of the pathophysiology of AN. Their multimodal meta-analysis of the neuroimaging studies in structural and functional brain alterations in patients with AN is one of the most recent complete and very informative reviews on this issue. The importance of examining brain findings in relation to maladaptive behavior present in AN has been highlighted before, pointing out the need for new techniques to better understand the neural mechanisms of the disorder [29].

One of the many possible other reasons that AN is hard to treat is that cognitive flexibility is found to be impaired in these patients [30]. In Aristotle’s experiment of the present study, cognitive flexibility is a crucial construct since, during the experiments, the subjects are facing “harsh” cognitive loads emanating from the structural complexity of Aristotelian syllogisms. Thus, the study of Sato et al. [30] is very relevant to our study since they examined the brain activity of patients with AN during a task computing cognitive flexibility. While their task was the Wisconsin Card Sorting Test (WCST) (one of the most widely used neurocognitive measures of cognitive flexibility and problem-solving), our task consisted of Aristotle’s valid and invalid syllogisms and paradoxes. Sato et al. [30] found that patients with AN performed significantly poorer than controls (their correct rates on WCST were poorer), exhibiting *weak activity* in the *right ventrolateral prefrontal cortex* and *bilateral para-hippocampal cortex* on set-shifting [31] in relation to controls. A positive correlation between correct rate and ventrolateral prefrontal activity in response to the set-shifting was found in healthy subjects, whereas patients failed. Thus, patients with AN showed impaired cognitive flexibility due to ventrolateral prefrontal and para-hippocampal cortex dysfunction. 

In order to provide a review of the available literature associated with the functional neuroimaging (SPECT, PET) of AN and outline the possible role of neurobiological factors in its pathogenesis, Pietrini et al. [32] performed a systematic review of the literature, mainly from PubMed database (from 1950 to 2009). Their main conclusion is that the most consistent alterations in AN cerebral activity seemed to involve the dorsolateral prefrontal cortex, the inferior parietal lobule, the anterior cingulate cortex, and the caudate nucleus. Therefore, *different neural systems may be affected, such as the frontal visual system, the attention network, the arousal and emotional processing systems, the reward processing network, and the network for the body form or appearance*. 

Patients with AN also exhibit the so-called “Brain fog”, a mild cognitive impairment, with symptoms such as impaired cognition, reduced concentration, and short- and long-term memory loss, while tasks that involve mental activity take longer. Kaufmann et al. [33] investigated structural changes in AN using T1-weighted magnetic resonance images with surface-based morphometry. Compared to controls, patients with AN showed globally decreased cortical thickness and subcortical volumes at baseline, i.e., findings indicating that structural brain alterations in adults with severe AN recover independently of the duration of illness during weight-restoration therapy. Food craving regulation in patients with AN, from its clinical and neurophysiological correlation to their emotions, is the aim of the paper by Mallorquí-Bagué et al. [34]. They examined how difficulties in emotion and craving regulation have been linked to the eating symptomatology of AN. Both patients with AN and healthy controls (HC) were involved in the process of completing a computerized task during EEG recording and were instructed to down-regulate negative emotions or food cravings. The subjects completed self-report measures of emotional regulation and food addiction, and the EEG signals P300 and Late Positive Potential (LPP) ERPs were analyzed. The main conclusion is that the AN group, in comparison to the HC group, displayed greater food addiction, greater use of maladaptive strategies, and emotional dysregulation. 

The quantification of cognitive deficits in severe AN before and after medical stabilization was the main aim of the study by Rylander et al. [35]. The primary outcome of their study was the alteration in test scores on the Repeatable Battery for the Assessment of Neuropsychological Status (RBANS) at baseline and after medical stabilization. The main conclusion is that baseline deficits in cognition were not documented in this sample of women with severe AN. No differences were found in core aspects of cognitive function in patients with long-lasting AN, who exhibited better concentration and grammatical reasoning accuracy in relation to healthy controls [36]. On the other hand, given the notion that information processing in AN is characterized by cognitive biases that affect patients’ behavior, Tenconi et al. [37] found that impaired ability to integrate disconfirmatory evidence in the reasoning process of patients with AN is not correlated with clinical characteristics. Differences in the performance of patients reported before when examining visuospatial processing have been attributed to information processing bias in AN determined by the requirements of each test. The finding of a bias toward local information processing at the level of detail in a demanding visual search task is in accordance with the psychopathology of the disorder, reflecting difficulties in central coherence [38]. Impaired cognitive flexibility and weak central coherence (i.e., the ability to overview complex information) are intended as maintenance factors in AN and targeted in specialized therapies [39].

The study of Collantoni et al. [40] aimed to explore inhibitory control capacity and its functional connectivity correlates in patients with AN. As it is known, *inhibitory control* (an executive function that allows behavior to be adapted according to environmental conditions) has been examined using the *Stop-Signal paradigm.* Compared to the healthy controls, AN patients have exhibited difficulty in response inhibition as well as a disruption of the functional connectivity of the VAC neural network (*ventral attention circuit*), which is involved in behavioral response in case of an unexpected stimulus. This malfunctioning of the VAC present in patients with AN indicates impairment of bottom-up signal filtering, which might be involved in difficulties in adapting behavioral responses to external stimuli (as in our case, the effort that patients with AN exert in handling difficult cognitive tasks, i.e., demanding Aristotelian logic). Moreover, recent data from an ERP study by Yue et al. [41] report demand-related alterations in fronto-central N2 latencies, further supporting the hypothesis of an abnormal inhibitory control process in AN.

The complexity of the etiology of AN, i.e., of the stages from vulnerability to illness, is well and thoroughly described by Kaye et al. [42]. Additionally, Connan et al. [43] incorporated different factors in their work to “establish” a concrete neuro-developmental theoretical framework for the causes-etiology of AN. 

#### 1.3.2. Feature Extraction and Selection Methods Used in EEG Classification Problems

In classification schemes, a variety of feature extraction and selection methods are used and are *time-* and- *frequency-oriented*. Acharya et al. [44] applied a nonlinear and wavelet-based feature technique for detecting epileptic EEGs automatically. These methods, based on time or frequency or both approaches, extract features from EEG that are input in classification models, the main target of which is to attain the highest possible performance in classifying subjects into healthy and pathological classes (a short description of the classification assessment criteria is provided in 2.8).

*Time domain features* include Permutation Entropy (PE), Approximate Entropy (AppEn) (used in this work), as well as nonlinear features as Lyapunov Exponents (LyapEx) [45,46,47,48,49,50], Correlation Dimension (CorrDim) [51], Higuchi Fractal Dimension or Curve (HFD), also used in this study [52,53,54], Hurst Exponent [55,56], and Hjorth parameters [57,58].

An example of using an Entropy feature in the analysis of signals (time series) recorded from patients with AN is described in the work of Jelinek et al. [59], in which the objective was to detect changes in heart rate variability (HRV) in response to orthostatic challenge. They concluded that the *Sample Entropy (SampEn)* and *Higuchi FD* features extracted from HRV signals are more sensitive in identifying sympathetic and parasympathetic changes connected to orthostatic challenges in patients with AN. SampEn was also used in a very recent (2023) paper by De la Cuz et al. [60] in analyzing the dynamic changes in the central autonomic network (CAN) of patients with AN. The authors have found that *patients exhibit higher complexity* in the functional connectivity (FC) time series, recorded via fMRI, thus providing evidence that core regions of the CAN involved in cardiac regulation are functionally affected in AN. Two important papers regarding the strength and “suitability” of various Entropy and Complexity measures are the works of Ferenets et al. [61] and Ahmadi et al. [62]. We used these sources to compare and comment on the “conflicting” results of HFD and AppEn on Anorexia data, as described in the Section 3 and Section 4.

One of the main objectives of this paper is the computation of two nonlinear measures, the *HFD* and *AppEn*, based on EEG recorded from healthy participants and patients with AN, to quantify their overall brain complexity during a cognitive task experiment and use these extracted features as input to various classifiers to categorize healthy controls and patients with AN. Both HFD and AppEn provide quantitative assessments of complexity and have been extensively used in EEG analysis (see the References Section). The main reasons for their choice are: (a) they have been proposed as powerful indicators in several recently published papers in the EEG analysis of various disorders (ASD, ADHD, etc.) and in Eating Disorders as in AN, (b) their calculation is straightforward and computationally efficient, and (c) the algorithms give reliable and consistent results (see Section 2.4 and Section 2.5), so they can be applied in real-time patient monitoring. In conclusion, we stress the necessity of interpreting complexity measures in the context of the specific analysis and features of the EEG time series under examination (as in our case, EEG taken from patients with AN).

#### 1.3.3. Measurement of Brain’s Responses during Demanding Cognitive Tasks—The α, β, δ, θ Specific Frequency Bands (Rhythms)

Changing levels of cognitive stimuli (e.g., due to exposure of subjects to an Aristotelian syllogism) induce neuronal responses that EEGs can effectively measure. The measurements of cognitive load (the load of working memory-WM during a demanding cognitive task) have been extensively reviewed during the last 20 years. EEG signals are composed of several frequency bands, called Rhythms or Waves, such as delta-δ (0.5–4 Hz), theta-θ (4–7 Hz), alpha-α (8–13 Hz), beta-β (13–30 Hz), and gamma-γ (>30 Hz). The alpha rhythms in the human EEG signals are the most prominent and dominant, playing different roles in different mental assessments. 

During resting states, the Power Spectral Density (PSD) of alpha waves remains *synchronized*, while during a (cognitive) task, it is increasingly *desynchronized* [63]. The same authors found in another work that alpha and beta waves play a major role in quantifying a cognitive load, with the properties of delta rhythm being capable of discriminating the hidden separate mental states. The study of brain dynamics during cognitive tasks (as in our case, Aristotle’s syllogisms), using different approaches, has become the main target of several research papers [64]. They stress the idea that alpha waves have been found to discriminate between the various cognitive loads at different brain states. Using Detrended Fluctuation Analysis (DFA), a “detector” of long-range correlations in a signal, Karkare et al. [65] tested their existence in EEGs taken from artists and not artists when they were performing perception and mental imagery tasks. They found that low-frequency waves, such as the *alpha*, are the primary signal to reflect the brain’s cognitive processing. *Do alpha or beta waves play a similar role in our case, i.e., during Aristotle’s syllogisms? And if yes, how do their dynamics change with the group of subjects (AN and Control)?* The reader will find the answers at the end of this study. 

*Frequency domain features* are based on the EEG signal’s power (energy), absolute or relative, or on their ratio. In this study, we consider the relative energy, computed within the context of the wavelet analysis (described in Section 3.6), of each separate rhythm (α, β, θ, etc.) with respect to the total energy, i.e., the sum of energies of all individual rhythms. For each participant’s (in our experiment) 14 channels of EEG data, the extracted descriptive feature vectors include the relative of normalized rhythm energy δ/Ε, α/Ε, β/Ε, γ/Ε and θ/Ε, where E is the energy of the total rhythm.

A work that is very relative to our work, at least with respect to the extracted EEG rhythm relative power (via wavelet analysis), is the work of Amin et al. [66]. The authors examined the dynamics of complex cognitive tasks using four classifiers, among which SVM provided the highest accuracy, 98%.

A combination of the extracted frequency- and time-domain-based features, using Stockwell transformation [67], is proposed by Hariharan et al. [68] to analyze baseline (at rest) and mental tasks related to letter and multiplication of numbers. K-means Nearest Neighborhood (KNN), linear discriminant analysis (LDA), and SVM are the classifiers used, exhibiting 84% to 98% accuracy. Empirical Mode Decomposition (EMD), another powerful tool based on the time-frequency combination, was applied by Noshadi et al. [69] to extract EEG rhythms for assessing various demanding cognitive loads. The authors report good performances of the SVM and KNN classifiers.

#### 1.3.4. EEG Rhythms α, β, δ, θ Specifically in AN

Although there are different aspects considering the neurophysiology of AN, to the best of our knowledge, there are few studies associating EEG rhythms with AN pathology. Since in this work, we examine the response of patients with AN in *cognitive loads* “imposed” on them by Aristotelian syllogisms, it is natural to search for relevant works that deal with cognitive difficulties that patients with AN face [70,71]. These patients also may often exhibit other personality and emotional characteristics like neuroticism, rigidity, introversion, and obsessive-compulsiveness [72,73]. Low-shifting abilities in AN are reported by Wu et al. [74], and weak central coherence is reported by Lang et al. [75] and Lopez et al. [76]. Taking into consideration that the Aristotelian syllogisms are expected to exert cognitive loads that require a great effort from the participants (both healthy controls and patients with AN), any subsequent cognitive difficulties will be reflected as differences in the brain activity between the subjects of these two groups, in possibly different EEG electrode (channel) locations. Thus, although many patients exhibit normal EEGs [77], it is logical to expect that a significant number of patients with AN may exhibit irregular background activity or even pronounced atypical EEG patterns of spike discharges. Crisp et al. [78] provide a control study of the dynamic behavior of patients with AN. Therefore, a *quantitative* analysis of these EEGs is welcomed to detect any differences in brain activity in localized regions by using *linear and nonlinear quantitative features* extracted from cleaned EEGs, which are considered to have *strong discriminatory power*, the ability to capture effectively “hidden” and underlying differences in the brain activations of the two groups.

In this work, six such features are used: four EEG rhythms of specific frequency bands, the alpha (8–13 Hz), beta (13–30 Hz), delta (1–4 Hz), and theta (4–8 Hz), as well as two nonlinear dynamics-oriented features (Higuchi fractal Dimension, HFD, and Approximate Entropy, AppEn). The traditional summary features were included to compare and demonstrate their complete failure in revealing differences. 

Regarding how EEG rhythms α, β, δ, θ “behave” in patients with AN, Rodriguez et al. [79] have reported *reduced alpha* rhythm amplitude in posterior regions, while Hatch et al. [80] and Toth et al. [81] have also reported *reduced alpha* rhythm amplitude in anterior regions and across frontal, temporal and parietal areas, respectively.

The results for the theta rhythm vary significantly: patients with AN *increased* posterior theta [80] and *normal* theta amplitudes [79] have been reported when they are in *relaxed wakefulness.* In this condition, (out)patients are rarely found to exhibit slow delta activity (a more severe brain malfunction). Increased theta amplitude was documented in frontal regions in patients with AN compared to healthy controls in a relaxed condition with closed eyes. This finding, accompanied by reduced relative alpha and delta amplitudes found among patients with AN, could possibly be related to increased attention/arousal [82]. 

To the best of our knowledge, no studies exist regarding the amplitude dynamics of α, β, δ, θ rhythms in patients with AN participating in a very demanding cognitive experiment like the one described in this study.

## 2. Materials and Methods

### 2.1. The Workflow

**Figure 1:**
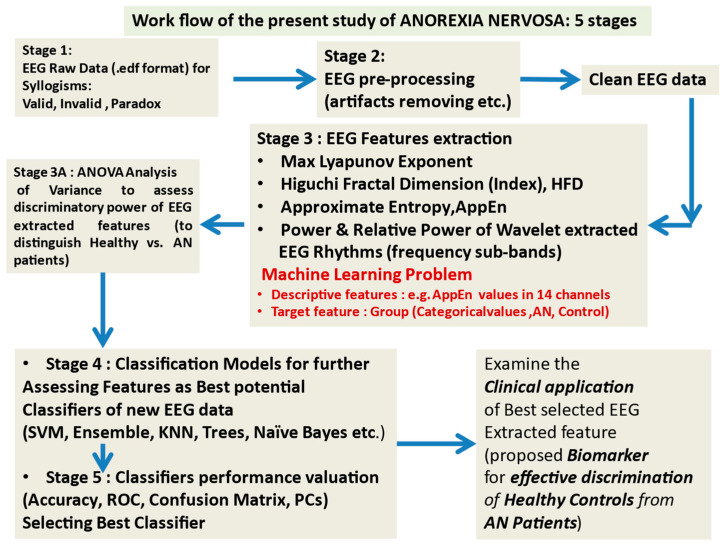
The workflow used in this work.

Figure 1 depicts the workflow adopted in this work. Raw EEG data (in xxxx.edf format suitable for using the EELAB tool, a powerful tool operating in MATLAB environment, for analyzing EEGs) are available and recorded from subjects of the two groups separately for each syllogism. Removal of artifacts and noise is performed at the stage of EEG preprocessing, and clean data is used to compute the extracted descriptive features (AppEn, HFD, etc.). A multivariate analysis of variance MANOVA is performed to assess the suitability of each feature for further analysis. From this stage, the most suitable feature is chosen based on its capacity to effectively and accurately separate the subjects of the two groups, Control and AN, using the results of the multivariate and between-subjects tests of the MANOVA. In Appendix A we provide information on the matrixes that contain the values of the extracted features. Then, all features are entered into various classification models or classifiers, and their classification performance is evaluated based on several quantitative and visual tools. The final stage examines the potential of combining the most significant syllogisms with the best-selected features (in our case, specific EEG waves and AppEn) and the best-found classifier (*presenting the higher accuracy*), with possible application in clinical practice for classifying healthy subjects and patients with AN. 

### 2.2. Participants and EEG Recordings

Sixty-one adult volunteers participated in this study. The group of anorexia nervosa comprised 31 patients with a mean age of 29.97 years (SD = 9.47) from the Eating Disorder Unit of the 1st and 2nd Department of Psychiatry, Medical School, National and Kapodistrian University of Athens. The group of patients consisted of 28 women and 3 men (mean ± SD age of 29.07 ± 9.54 years and 38.33 ± 1.15 years respectively). The mean Body Mass Index (BMI) of patients was 14.72 (SD = 2.03). An AN diagnosis was made through a clinical interview conducted by an experienced psychiatrist, according to the Diagnostic and Statistical Manual of Mental Disorders, Fifth Edition (DSM-V) [22]. Inclusion criteria were: (a) age <50 years, and (b) patients not on any medication. 

The control group comprised 30 healthy subjects (mean age = 31.03 years, SD = 7.15), of which 25 were women and 5 men (mean ± SD age of 30.52 ± 6.96 years and 33.60 ± 8.35 years respectively), with a mean BMI of 21.50 (SD = 1.48). Eating disorders or other psychiatric disorders were excluded by clinical interview. 

The sample of patients and healthy controls was homogeneous in age, as confirmed by an independent-sample *t*-test (t (59) = −0.495, *p* = 0.623). BMI differed significantly between groups (*p* < 0.001), as expected. No significant differences in educational background between groups were documented (*p* = 0.084). 

All participants had normal or corrected vision, spoke Greek as their native language, and had no history of neurological disorders. They signed an informed consent form after being widely informed about the procedure. The study was approved by the Scientific Council and received ethical approval by the Institutional Bioethics Committee and was conducted in collaboration with the First Department of Psychiatry, Medical School and the University Mental Health, Neurosciences, and Precision Medicine Research Institute “Costas Stefanis” (U.M.H.R.I).

**Figure 2:**
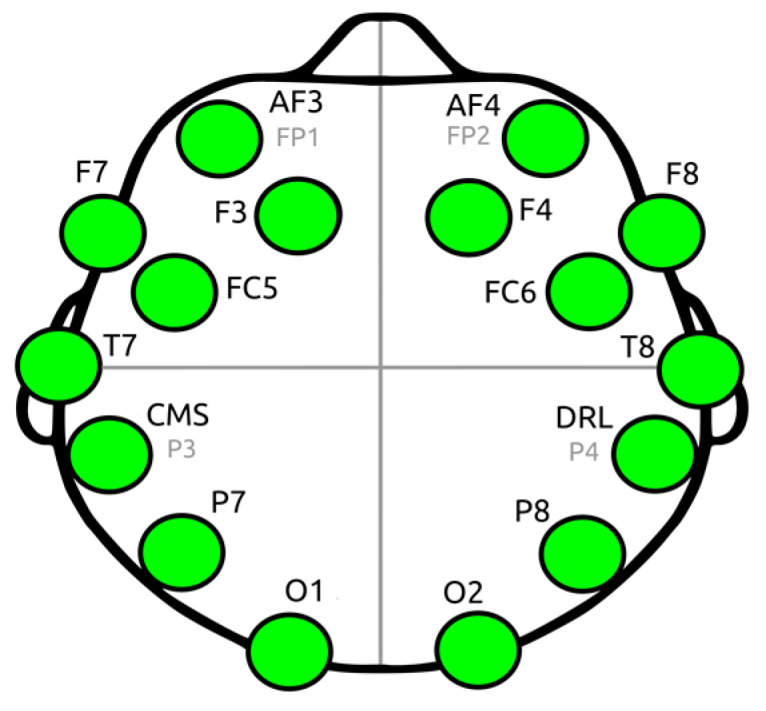
EEG recording electrode scalp locations in emotive EPOC system.

**Figure 3:**
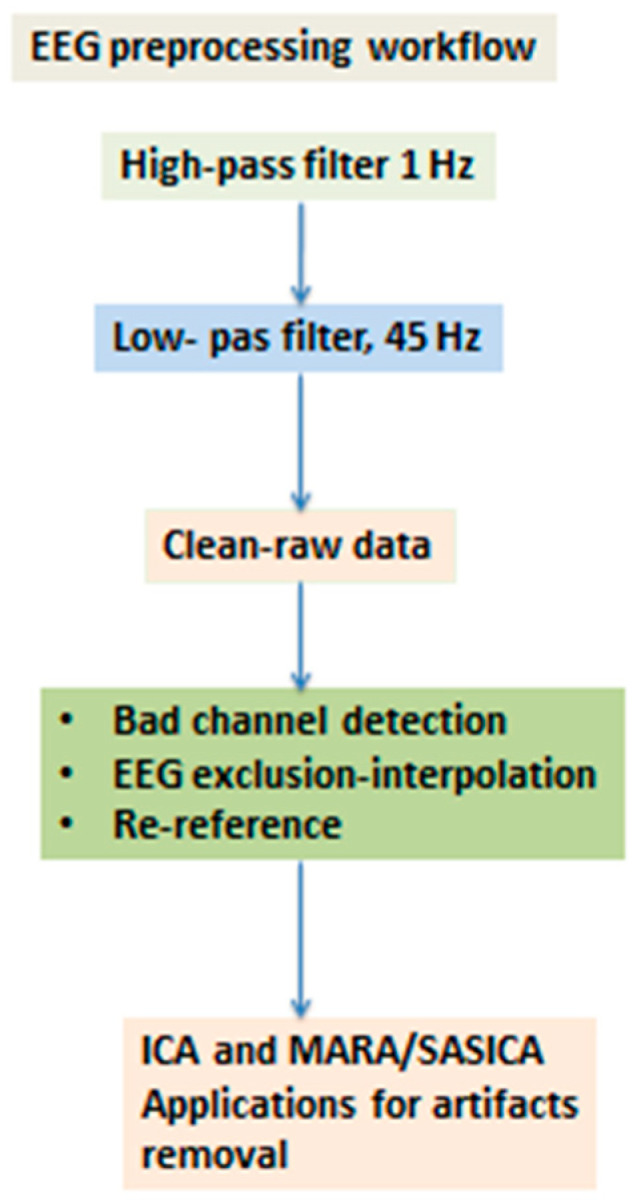
Workflow of EEG preprocessing.

In our study, the Emotiv EPOC device has provided considerable benefits compared with other multichannel equipment: (i) the setting up time of the Emotiv EPOC system is substantially shorter compared to an expensive EEG system, (ii) additionally, previous research estimating the reliability of the Emotiv EPOC EEG device provides evidence indicating its capacity to measure consistently EEG signals [83,84]. The EEG signals were recorded from 14 Ag/AgCI electrodes mounted on an elastic cap, in accordance with the international 10–20 System (see Figure 2 and Table 1) [20]. The *sampling frequency* was 128 Hz, and electrode impedance was kept continuously below 5 K Ω. The EEG activity was referenced to the mean of the left and right ear lobes by an online setup. The ground electrode was placed on the mastoid. For each participant, the continuous EEG signals were high pass filtered at 1 Hz to remove DC offsets and various drifts. Then, the signals were low-pass filtered at 45 Hz. Using the EEGLAB 2020.0’s version [85] plug-in functions, “clean-raw data” were examined for bad channels, also using a visual inspection of the resulting bad electrodes; the electrodes then exhibiting abnormal time course were excluded and interpolated. The EEG signals were re-referenced to the whole scalp common mean EEG value. An Independent Component Analysis (ICA) of the continuous data and the MARA-SASICA tools [86,87] was performed to correct eye blinks and saccades. Both software tools automatically labeled artefactual components as candidates for rejection. The average number of rejected components was larger in the case of patients with AN than in the case of healthy controls. After cap fitting, good conductivity was confirmed with Emotiv software(Emotiv EPOC+, Emotiv Inc. USA) through wet saline electrodes [88]. From an initial group of 31 patients, only twelve (12) were kept for further analysis (feature extraction from their EEGs) since the EEGs of the remaining 18 subjects were excluded due to many artifacts and other “noises”, possibly due to excessive motions of patients and the subsequent large EEG signals’ amplitudes. One patient was excluded due to missing EEG data. The reduced number of patients with AN is, however, adequate for statistical comparisons and in accordance with other studies found in the literature. Figure 3 depicts the main stages of the workflow of EEG preprocessing followed in this work.

### 2.3. Cognitive Tasks Description in Aristotle’s Experiment

Using EEG signals, we aim to isolate the brain regions involved in Aristotelian syllogisms and differentiate their engagement during the different types examined: valid, invalid, and paradox. In addition, we will try to answer how this differentiation is accounted for in the case of two groups of subjects (Control, AN). 

The sentences or prepositions in the syllogisms examined were presented visually (on a computer screen) to dissociate brain regions related to syllogism processing from those related to sensory processes and low-level reasoning. This is an essential stage in the analysis process, considering that isolating substrates associated with these high-level syllogisms (activating cross-modal cognitive systems) is difficult. In our experiment, the syllogism statements consisted of categorical types, including different types of deductive arguments (valid, invalid) and paradoxical reasoning. Participants were asked to reach a conclusion from the premises following the instructions explained by the instructor and indicate whether the given statement was “Right” or “Wrong” (see below).

Aristotle’s experiment is based on the study of reasoning or syllogism process depending on logical rules and considers the reasoning process (how do we conclude) and the decision-making process. The process starts with assumptions (hypotheses) that ideally lead to a valid conclusion. 

If S and P are used to denote the subject and predicate of the conclusion, then, there must be some third entity involved in the two premises to draw an inference. This additional entity is called the middle term (M).

*All men are mortal. (MaP)*
*Socrates is a man. (SaM)*
*Socrates is mortal. (SaP)*

Thus, each proposition or statement belongs to a group of four forms or patterns [10,12,13,89]. Eliminating the particular in this way sets the stage for examining the abstract patterns of reasoning.

*(All S is P)*
*(Some S is P)*

Aristotle’s contribution is significant because he abstracted a general pattern from such examples. This step is similar to the step of replacing numbers with algebraic symbols such as the letters x, y, and z [89]. The gap between the subject and the predicate of each proposition can be filled with any one of the four letters a, e, i, or o, giving abbreviated forms that clarify the abstract patterns of propositions that Aristotle was looking at. In this framework, it should be noted that one of Aristotle’s major accomplishments was to find all the valid ones [10,89].

These patterns give 4 × 4 × 4 × 4 = 256 possible syllogisms. From all possible patterns, not all are valid. Aristotle first discovered the list of valid syllogisms, while the rest of the syllogisms are invalid [89]. The four forms or patterns mentioned above are called *figures.* Only 19 syllogisms have a logical conclusion (they are *valid*), even though the validity of a syllogism is a relatively controversial concept [10].

According to the model of a dual process theory of reasoning, it has been claimed that the presence of two different cognitive systems underlies reasoning: system I (heuristic system) is considered older, fast, and automatic, tending to solve problems by using prior knowledge and beliefs, while system II (analytic system) is considered evolutionarily newer, slower and allows reasoning based on logical rules within the limits of working memory resources [14,90]. In type I, we are aware only of the result (conclusion), while type II includes both the procedure and the result. Usually, there is an interaction between the two types of reasoning. Previous evidence suggests different neurobiological substrates for these two systems of reasoning [90,91]. The first system is considered to activate mainly fronto-temporal areas, while the second system engages working memory and parieto-occipital areas [1]. Inductive and deductive reasoning processes could be directed by both these systems, yet in different parts, the first depending more on aspects such as previous knowledge (system I), while the last is considered to include the logical validity of an argument (system II) [92].

The question that often arises is whether different types of syllogisms engage different cognitive mechanisms. Aristotle’s experiment is a process consisting of four stages. Valid and invalid syllogisms are used, together with paradoxes and illusions-visual paradoxes (not considered in this study). 

More specifically, the participants were exposed to three different sets of syllogisms (valid, invalid, and logical paradoxes), each one containing 39 syllogisms [1,21], as well as to a set of 39 optical illusions [93]. A further explanation of the types of syllogisms used in our study can be retrieved from previous works that followed different approaches using the same task [1,18,19,21].

The subjects under test were comfortably seated 1m away from a computer screen. Instructions about the procedure were presented to them before their exposition of the experiment so that they could be familiar with the process and its requirements. Then, syllogisms or arguments started to appear on the screen in sets of 39, every one of which was accompanied by a question “right”, or “wrong”. The time duration of each slide was proportional to the number of letters in the syllogisms, and a blank slide continuously replaced the slide. Immediately after, the next stimulus appeared. Each participant was driven to carefully read each statement, followed by the question “Right or Wrong?” and give a response, after two presentations of a warning stimulus, to a) whether the syllogism presented is right or wrong and b) the percent (%) of certainty for the response provided (from 0% not at all certain, to 100% absolutely certain).

Answers to each of the valid syllogisms are considered right if the subject considers them right, while answers to invalid syllogisms are considered right if the subject considers them wrong. Answers to paradoxical syllogisms depend on the subjective critical point of view, and therefore, the meaning of a wrong answer is not dogmatic [19]. Each subject’s answer is accompanied by the percent (%) of *certainty* given, which reflects the certainty in the answer provided. An indicative example of valid, invalid, and paradoxical syllogism used in Aristotle’s experiment is shown in Table 2. A complete description of the syllogisms used in the experiment is available in a previous work by Papageorgiou et al. [1]. 

In parallel with the process described before, the emotional condition of each tested subject (intensity, control of the emotions experienced that moment and mood) is recorded, according to the Self-Assessment Manikin (SAM), a technique that directly assesses the pleasure, arousal, and dominance associated with emotional reaction in response to a variety of stimuli [20,94]. Also, EEG signals of alpha, beta, delta, and theta frequency bands (and their sub-bands), are simultaneously recorded using a wireless system of 14 electrodes (EMOTIV PRO, see Table 1 above).

### 2.4. Approximate and Sample Entropy AppEn

According to information theory, entropy is considered an “uncertainty measure” of a given system. High entropy corresponds to no information flow, thus characterizing a system as random, while low entropy corresponds to deterministic systems. The approximate entropy (AppEn) [95], which is bounded, is utilized in the framework of this work. It is derived as follows:

Let *u* be a time series $u\left(1\right),u\left(2\right),...,u\left(N\right),$ of N observations. Given a non-negative number m,m≤Ν, we formulate m-blocks of *subsequences:*

$$x\left(i\right)\equiv \left(u\left(i\right),u\left(i+1\right),\ldots ,u\left(i+m-1\right)\right)$$

The distance between 2 blocks can be calculated as:

$$d\left(x\left(i\right),x\left(j\right)\right)\equiv \left(\left|u\left(i+k-1\right)-u\left(j+k-1\right)\right|\right)$$

which gives the maximum point-wise distance.

Given r∈R, for a given block $x\left(i\right)$, we calculate the *percentage* of blocks $x\left(j\right)$ with distance r, which we call $C_{i}^{m}\left(r\right)$.

Using the Heaviside Θ function, which counts how many times the distance exceeds the given threshold r, we define:

(1) $$C_{i}^{m}\left(r\right)=\frac{1}{N-m+1}\sum_{j=1}^{N-m+1}\Theta \left(r-d\left(x\left(i\right),x\left(j\right)\right)\right)$$

(2) $$\Phi^{m}\left(r\right)=\frac{1}{N-m+1}\sum_{i=1}^{N-m+1}logC_{i}^{m}\left(r\right)$$

Finally, AppEn is defined as:

(3) $$AppEn\left(m,r,N\right)\left(u\right)=\Phi^{m}\left(r\right)-\Phi^{m+1}\left(r\right),m\geq 0$$

(4) $$ApEn\left(0,r,N\right)\left(u\right)={-\Phi }^{1}\left(r\right)$$

$AppEn\left(m,r,N\right)$ is always a well-defined function. From Equation (1), when a percentage of the block is <1, the distance from itself $d\left(x_{i},x_{i}\right)$ is always measurable. It follows that, $C_{i}^{m}\left(r\right)\geq 1$ and consequently, the logarithm of $C_{i}^{m}\left(r\right)$ is always defined.

The AppEn measures the degree to which different segments of the series follow similar patterns.

(5) $$-AppEn\left(m,r,N\right)\left(u\right)=\Phi^{m+1}\left(r\right)-\Phi^{m}\left(r\right)\;\frac{1}{N-m}\sum_{i=1}^{N-m}logC_{i}^{m+1}\left(r\right)-\frac{1}{N-m+1}\sum_{i=1}^{N-m+1}logC_{i}^{m}\left(r\right)\;\approx \frac{1}{N-m}\sum_{i=1}^{N-m}\left(logC_{i}^{m+1}\left(r\right)-logC_{i}^{m}\left(r\right)\right)\;\frac{1}{N-m}\sum_{i=1}^{N-m}log\left(\frac{C_{i}^{m+1}\left(r\right)}{C_{i}^{m}\left(r\right)}\right)$$

The last line in Equation (5), is the average logarithm, for all *m*-blocks, of the *conditional probability* $\left|u\left(j+m\right)-u\left(i+m\right)\right|<r$ given $\left|u\left(j+k\right)-u\left(i+k\right)\right|<r$ for all k=0,1,…,m−1, thus the approximate entropy estimates the logarithmic probability that sequences of patterns which are close for m observations will remain close. In this sense, the AppEn measures *persistence, correlation,* and *regularity*. Higher values indicate lower persistence and independence between the observations, while lower values of approximate entropy correspond to higher persistence, autocorrelation, and regularity. If $C_{i}^{m+1}\left(r\right)=C_{i}^{m}\left(r\right)$, the approximate entropy is 0, which corresponds to a weak serial correlation.

Another useful form of the AppEn comes from Equation (3), for m=1, so AppEn$\left(m=1,r,N\right)$ can be the expected log-likelihood value of two blocks that remain close to each other when they are expanded by one data point. Assuming stationarity of the stochastic process, the number of steps in calculating AppEn $\left(m=1,r,N\right)$ is significantly reduced as follows:

(6) $$AppEn\left(1,r,N\right)\left(u\right)=\Phi^{1}{-\Phi }^{2}=\frac{1}{N-1}\sum_{i=1}^{N-1}log\left(\frac{C_{i}^{2}\left(r\right)}{C_{i}^{1}\left(r\right)}\right)=-E\left(log\left(\frac{C_{i}^{2}\left(r\right)}{C_{i}^{1}\left(r\right)}\right)\right),\left(forN\to \infty \right)$$

The threshold r can be seen as the parameter that differentiates the measured distances (as stated by Takens), while the embedding dimension m is the number of adjacent points. Thus, AppEn measures the degree to which different segments of the time series follow similar patterns.

For a random process, AppEn $\to log\left(\frac{r}{\sqrt{3}}\right)$ for all m [95], while for a purely deterministic process, AppEn →0. We can, therefore, rescale so that 0≤AppEn≤1, where 1 defines a purely random process, which is the characteristic of an efficient market.

$$0\leq AppEn\leq 1\left(afterrescaling\right)$$

A modification of the AppEn has been recently proposed [96], called *Sample Entropy, SaEn*, which has the advantage of being less dependent on the length of the time series and exhibits relative consistency over a broader range of possible r,m∧N. On the basis of *K*_2_ entropy, Richman and Moorman defined the parameter:

(7a) $$SaEn\left(m,r\right)=\left(-log\frac{U^{m+1}\left(r\right)}{U^{m}\left(r\right)}\right)$$

which is estimated by the statistic:

(7b) $$SaEn\left(m,r,N\right)=-log\frac{U^{m+1}\left(r\right)}{U^{m}\left(r\right)},N\to \infty$$

The differences between the previously defined $C^{m+1}\left(r\right)$ and $U^{m+1}\left(r\right)$ (similarly between $C^{m}\left(r\right)$ and $U^{m}\left(r\right)$), are due to (a) defining the distance between two vectors as the maximum absolute difference between their components, (b) vectors are not compared to themselves, and finally, (c) for a time series of length *N*, only the first *N-m* vectors of length *m*, $U_{m}\left(i\right)$, are considered by taking care of defining also the vector $U_{m+1}\left(i\right)$ of length *m* + 1, when 1≤i≤N−m.

SaEn is exactly equal to the negative of the natural logarithm of the conditional probability that *m* data sequences that are very close in proximity will also remain close to each other when one more point is added to the sequence. A detailed algorithm for calculating SaEn is given by Costa et al. [97]. Now let $n_{i}^{m}\left(r\right)$ represent the number of vectors $x_{m}\left(j\right)$ that are close to the vector $x_{m}\left(i\right)$ i.e., the number of vectors that satisfy $d\left(x\left(i\right),x\left(j\right)\right)\leq r,$ where d is the distance. Then we note that:

(8) $$ApEpn\left(m,r,N\right)≅\frac{1}{N-m}\sum_{i=1}^{N-m}log\frac{n_{i}^{m}}{n_{i}^{m+1}}$$

and

(9) $$SaEn\left(m,r,N\right)=log\frac{\sum_{i=1}^{N-m}n_{i}^{\prime m}}{\sum_{i=1}^{N-m}n_{i}^{\prime m+1}}$$

where $n_{i}^{\prime m}$ differs from $n_{i}^{m}$ to the extent that for SaEn, self-matches are not counted $\left(i\neq j\right)$ and 1≤i≤N−m.

A smaller value of sample entropy indicates a lower level of complexity or more self-similarity in a data series. SaEn is largely independent of record length and displays relative consistency under circumstances where AppEn does not [96]. 

The AppEn approximates the Renyi entropy of order q=1 (corresponding to Shannon entropy) and SaEn the Renyi entropy for q=2. The SaEn has the advantage of being an unbiased estimator [98].

Both SaEn and AppEn measure the degree of randomness (or the degree of orderliness, inversely). However, it is pointed out that there is no direct relationship between regularity, as measured by entropy-based metrics, and complexity. An increase in complexity is usually but not always associated with an increase in entropy. Entropy-based metrics are maximized for random sequences, although it is generally accepted that both regular or perfectly ordered and complete irregular or disordered systems process no complex structures. A meaningful complexity measure of a brain signal, therefore, should vanish for these two extreme states. The Approximate and Sample Entropy can contemplate the existing nonlinear dynamics in Brain EEG signals and are also considered as *alternative volatility* measures to quantify the *degree of randomness or determinism* of these signals. By adopting the entropy concept as an alternative measure of volatility, the assumption of the i.i.d process is not needed.

### 2.5. Higuchi Fractal Dimension (HFD) Measure 

Complexity is quantified via an index based on the fractal dimension, FD, that reflects how the measure of the length of a curve *L(k)* varies in relation to the scale k used as a unit of measurement. There are various approximation techniques for estimating the fractal dimension [99]. One of the most often employed methods in EEG analysis is Higuchi’s algorithm [52] in estimating the fractal dimension D [53,54]. In *Higuchi’s* time-domain approach, the estimation of FD of a signal (time series) is based on the *log*[*L*(*k*)] versus *log*(*k*) curve of the time series, computed as follows:

(a) Let an EEG epoch is *s* = {*s*(1), *s*(2), …, *s*(*N*)} of length N, then for each epoch’s sample *i*, we compute the absolute differences between values *s(i)* and *s*(*I* − *k*) (i.e., the samples at distance *k*), for *k* = 1, …, *kmax*.
(b) We multiply each absolute difference by a normalization coefficient taking into consideration the different numbers of samples that are contained for each value of *k*. The coefficient is computed based on the starting point *m* = 1,…, *k* as well as on N, the total number of data points of an epoch.
(c) Thus, the *Lm*(*k*) is estimated as:

$$L_{m}\left(k\right)=\frac{1}{k}$$

where *q = int[(N − m)/k]*, and *L(k)* is computed as follows:

(10) $$L\left(k\right)=\frac{1}{k}\sum_{m=1}^{k}L_{m}\left(k\right)$$

Let *lk* indicates the *log[L(k)]* versus *log(k)* curve. Let now *klin* defines the upper range of the linear region of *lk,* then by definition, if *L(k)* is proportional to $k^{D}$ for *k =* 1*, …, klin*, then the curve is *fractal* with dimension D. Thus, *as the signal irregularity increases, Higuchi’s fractal dimension D increases, too.* So, the *nonlinear part* of the *lk* curve (*k* > *klin*) corresponds to an oscillatory behavior with features depending on the *periodicity of the signal itself* [54]. 

#### 2.5.1. Interpreting and Comparing AppEn and HFD

These two nonlinear measures are used in EEG analysis to quantify their complexity or irregularity; however, they approach complexity assessment from a different perspective. Regarding HFD, this is based on the concept of fractals, i.e., *complex geometric patterns* exhibiting *self-similarity* across different scales. HFD measures a signal’s " roughness " by quantifying the (phase) space-filling capacity of the signal’s trajectory. Therefore, HFD measures the signal’s complexity on the base of its *fractal characteristics.* A high fractal dimension (measured, e.g., by correlation dimension) reflects a more complex or irregular signal, while a low one corresponds to a more regular or predictable signal.

On the other hand, AppEn measures the regularity or predictability of a time series by quantifying the probability that similar patterns will repeat in the time series, considering both the amplitude and the temporal structure as criteria of pattern similarity. Its numerical value reflects the regularity or complexity of the time series. High AppEn corresponds to higher levels of irregularity and complexity, while low AppEn suggests more regular and predictable patterns in the time series.

Thus, in conclusion, regarding the interpretation of the two measures, higher HFD indicates higher complexity or irregularity, while higher AppEn also reflects higher complexity or irregularity but approaches complexity from different perspectives. A crucial question is whether there are cases in which these two measures generate conflicting results, a fact that we have encountered in this paper, assessing the complexity of healthy controls and patients with AN by using both measures. 

The answer to the above-mentioned crucial question is that these two measures may yield opposite results due to the different aspects of complexity these two features capture and their underlying assumptions in their structure. Table 3 provides scenarios where conflicts may occur:

In scenario 1, the signal exhibits irregularity or complexity but lacks the characteristic self-similarity associated with fractals. Thus, HFD may yield a low value (lower complexity), while AppEn may still be high due to the signal’s irregularity.

In scenario 2, the signal exhibits clear fractal properties (e.g., via correlation dimension), thus indicating *high* HFD, but at the same time, the signal follows *predictable patterns* without much irregularity. Thus, AppEn may yield a *low value* (lower complexity) *despite the high fractal dimension.*

Finally, in scenario 3, we recall that HFD emphasizes the *spatial* and *self-similarity* characteristics of complexity, while AppEn is associated with *temporal irregularity* and *predictability*. Thus, certain types of complexity may be more pronounced (prevailing) in one measure in comparison to the other. Thus, HFD may be high in a signal with strong spatial self-similarity but lacking temporal irregularity, while AppEn is constantly low.

In this paper, considering two measures of complexity allows us to make comprehensive analysis and to integrate their “different and seemingly conflicting” findings to shed light on the underlying dynamics of the signals. An example of a deep but mainly technical-mathematical comparison of HFD, AppEn, and other complexity measures is the work of Ferenets et al. [61], who assessed the depth of sedation. In our paper, we have used the same values of the parameters involved in HFD and AppEn, as in the work of Ferenets et al. (actually, for AppEn, m = embedding dimension = 2, r_f_ = 0.2×(sd), and Max interval time = K_max_ = 8, for HFD). In general, our findings of HFD and AppEn for the EEG signals taken from healthy controls and patients with AN are in accordance with the findings of the paper of Ferenets et al. regarding the values of the parameters used in our two measures. 

### 2.6. Wavelet Extracted EEG Power Rhythms (Waves, or Frequency Sub-Bands) as EEG Feature Potential Biomarker

**Figure 4:**
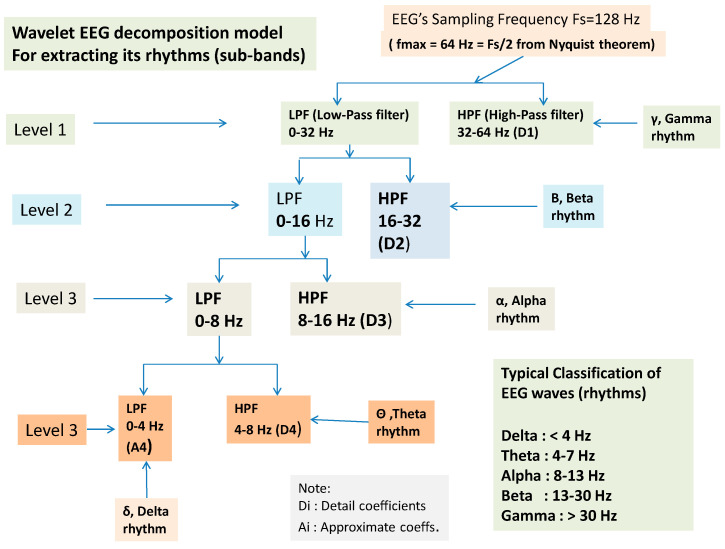
Wavelet EEG decomposition process for extracting its rhythms (frequency sub-bands alpha, beta, etc.).

In this work, we applied wavelet analysis to extract specific information from our raw EEGs. We extract EEG power rhythms or waves of specific frequency sub-bands that are associated with specific brain functions. It is an analysis in both the time and frequency domain. In this paper, Daubechies wavelet (DW) is chosen due to several advantages described by Gowri and Raj [100].

(11) $$Y_{low}\left[n\right]=\sum_{k=-\infty }^{\infty }s\left[k\right]*g\left[n-k\right]\;$$

(12) $$Y_{high}\left[n\right]=\sum_{k=-\infty }^{\infty }s\left[k\right]*h\left[n-k\right]$$

where $s\left[k\right]$,$\;g\left[n\right]$, and $h\left[n\right]$ indicate the *input signal*, and *impulse response* of *low-pass* and *high-pass* filters, respectively. The output of the filter $Y_{low}$ and $Y_{high}$ must then be down-sampled by 2, as follows [101]. The required sampling frequency $f_{s}$ must also decreased to half since $f_{s}\geq 2f_{max}$ according to the sampling or Nyquist theorem [102] and because the maximum frequency $f_{max}$ decreases due to filtering. Following down-sampling, the sample length decreases to half, and the scale is doubled. In this work, DWT (Discrete Wavelet Analysis) is performed using MATLAB R2018b environment. In this paper, the sampling frequency is 128 Hz, and DWT decomposes the EEG signals of all channels into several frequency sub-bands via DW. Figure 4 shows each decomposition level, and each frequency sub-band represents different brain waves, i.e., electrical activities that reflect specific brain functions such as emotion, reasoning, behavior, etc. The primary brain rhythms or waves are shown in the figure (alpha, beta, theta, delta, and gamma). *Delta rhythm* (0–4 Hz) is a loud and slow wave that occurs during dreamless sleep and meditation. When the subject is in an alert state of consciousness, an *Alpha wave* (8–16 Hz) occurs. *Theta wave* (4–8 Hz) occurs during sleep in the presence of dreams as well as in a meditative state [103]. *Beta wave* (16–32 Hz) is often enforced during drowsiness and is considered to be related to the functions of the sensorimotor cortex. Excess of beta activity is considered abnormal. *Gamma waves* (32–64 Hz) are the fastest of all brain waves, occurring in parallel processing of the brain and can be captured in a calm mind [103]. We do not examine gamma waves in this work. EEG signal is decomposed into approximation and detail coefficients, and each one of them is further decomposed to obtain the desired level of decomposition, which, in our case, is three levels, and the features are extracted from detail (D1 to D4) and approximation A4 (see Figure 4).

The power of each frequency band was calculated by averaging the power values within the above bands (rhythms), and electrode and band-specific time-course were calculated for each participant.

The *absolute power* is associated with how much brain power is available and represents the electrical power in each band of EEG. It is compared to all other individuals in the data set to determine whether the results are typical or atypical. The voltage produced by the brain is measured at each of the channels and is used in detecting whether enough brainpower within a specific frequency range is present at each recording channel. The *relative power* can be realized as the power in one frequency band compared to all other bands or the distributed total amount of power at each brain location. It is used to determine whether a specific frequency (e.g., alpha) is *overpowering other vital brain frequencies* or if the power is *low.* In this paper, we have also computed the relative power of α, β, γ, δ, θ rhythms as separate extracted features for each group of subjects and each syllogism. For example, the relative power of alpha is estimated as follows: 

Alpha Relative Power = Alpha Power/(Total power), where Total Power = Alpha power + Beta power + Delta power + Theta power + Gamma power

### 2.7. Classification and Used Classifiers

This section provides a short description of classifiers and an evaluation of their performance to make this work as self-contained as possible. All the classification models developed in this work are implemented in MATLAB’s (vers. 2018b) *classification Learner app (CLA)* toolbox, which supports:

- *Decision trees*: Deep tree, medium tree, and shallow tree;
- *Support vector machines*: Linear SVM, fine Gaussian SVM, medium Gaussian SVM, coarse Gaussian SVM, quadratic SVM, and cubic SVM;
- *Nearest neighbor classifiers*: Fine *k*NN, medium *k*NN, coarse *k*NN, cosine *k*NN, cubic *k*NN, and weighted *k*NN;
- *Ensemble classifiers*: Boosted trees (AdaBoost, RUSBoost), bagged trees, subspace *k*NN, and sub-space discriminant.

With CLA, we assess classifier performance using confusion matrices, ROC curves, or scatter plots, compare model accuracy using the misclassification rate on the validation set, improve model accuracy with advanced options and feature selection, export the best model to the workspace to make predictions on new data and finally we can generate MATLAB code to train classifiers on new data. We compared the performance of several classifiers with their default parameter values to discriminate between patients with AN and healthy controls. Before entering the classifiers, all extracted features were normalized by subtracting sample means and dividing by sample standard deviation, rendering the inputs to have zero means and unit standard deviation. To enhance the effectiveness of their discriminatory capacity of the classifiers, a dimensionality reduction was performed via Principal Component Analysis (PCA) [104], which has the effect of decorating the features. Only the three (3) PCs were kept for further analysis, corresponding to the three largest eigenvalues of the sample covariance matrix. The percentage of the total explained variance by the first three PCs is estimated as the ratio between sums of variances of the 3 PCs and original variables (in our case of the 14 EEG channels). Some of the most important and widely used classifiers (automatically chosen to be the aforementioned toolbox) are the following: 

Naïve Bayes (NB) classifier [105,106]: Using Bayes’ theorem [107], NB is a maximum a posteriori classifier, generating the class c_i_ with the highest probability, given the observed feature values, in our case, the AppEn and HFD and the EEG rhythms. The product of posterior Gaussian probabilities [108] is used to estimate the posterior probability conditioned by the set of attributes $\left\{x_{1},\ldots ,x_{k}\right\}$ (assumed independent). 

Logistic regression, LR [106,109]: For this classifier, a linear combination of features and logistic regression function is used to estimate the class of the class conditional probability, and the model coefficients are computed via the LogitBoost algorithm [110].

Multi-layer perceptron (MLP) is a generalization of logistic regression enforced by incorporating an extra processing layer [106]. The class is estimated via thresholding the value of its output processing unit [110,111], which uses a non-linear transfer function on the linear combination of the generated outputs of hidden neurons, each one of which applies a transfer function to a linear combination of the inputs. The coefficients of the used MLPs with ⌈*k* + 1/2⌉ hidden neurons were computed by a backpropagation algorithm with default values of the toolbox of learning rate, momentum, and number of training epochs [111]. 

Support vector machines (SVM): A powerful approach to predictive modeling based on error-based learning [106]. They are trained in a slightly different way than regression models. The main advantages of SVM are that they are robust to overfitting and perform extremely good in very high-dimensional problems. The classification of this classifier is done by partitioning a feature space via a decision boundary that is linear in the transformed space, defined by the kernel function, and uniquely determined by a subset of data–support vectors [104]. The maximization of the distance between the decision boundary and the support vectors, performed by SVMs, determines a maximal margin classifier. A variety of kernel functions is used in the toolbox, chosen optimally for the given data. In general, by construction, SVMs maximize classifier margin and, hence, probably minimize overfitting. 

Decision trees (DT) [106,112]: In this model, the feature space is recursively partitioned in those regions that correspond to classes, selecting that feature that generates the highest level of information gain. The partition process follows specific rules, and the process ends if a predefined minimal number of samples per node of a decision tree is reached (usually 2 samples/node). The complexity of the DT model may be reduced via estimating classification error in the pruning stage [113], thus improving its generalization capacity. 

Random forests (RF): This model performs classification by combining classes predicted by ensemble members, utilizing an ensemble of unpruned trees [106,114]. For partitioning, the algorithm considers a random subset of the features in each node of a tree. In this work, parameters as members of the used ensembles and random features for each split are the default values chosen by the CLA. 

#### Evaluation of Classifier’s Performance

Classification accuracy was evaluated through a *cross-validation* procedure in which the dataset was split into K subsets of approximately equal size K-1 subsets were used to fit a classification model, and the remaining subset was used to evaluate the classifier. This procedure was repeated K times such that a classifier was evaluated in each subset. In this study, we used K = 10 [115,116]. The classification accuracy was assessed through overall accuracy—the percentage of correctly classified samples—and using the area under the ROC curve (AUC). The overall accuracy of useful classifiers in two-class problems ranges from 50% to 100%. The ROC curve is created by plotting the true positive rate (the proportion of samples with AN that are detected as such) vs. the false positive rate (the ratio of the total number of controls incorrectly detected as with AN and the total number of controls). AUC ranges from 0.5 (for a classifier that randomly guesses a class) to 1 (for an ideal classifier) [117]. In machine learning, model complexity (of the classifier, not of the signal) is defined as the ability of a classifier to distinguish among classes that are separated by multivarious surfaces. Here, model complexity was measured using the Vapnik-Chevronenkis (VC) dimension. Burges [118] indicates that the models with a small number of parameters may have larger VC dimensions and complexity. According to statistical learning theory [113], the classification accuracy on test data (measured by a ten-fold cross-validation method in this study) 14 decreases with a factor directly proportional to the VC dimension of the model and inversely proportional to the size of the training data set. Among the classification methods considered in this paper, the multilayer perceptron and decision tree have VC dimensions that increase with the number of features utilized for classification.

### 2.8. Assessing the Performance of Classifiers: The ROC (Receiver Operating Curve), the CM (Confusion Matrix), and the Parallel Coordinates (PC) Tools

In order to assess the ability of the two features-complexity measures (AppEn and Higuchi FD) and of the four EEG rhythms α, β, δ, θ) to discriminate patients with AN from control subjects at various (or possibly all) electrodes, we use various tools as the Confusion Matrix (CM) and receiver operating characteristic (ROC) plots [119,120]. We used Matlab’s functions that automatically select different thresholds or cut-off points of the complexity measures values and compute their sensitivity/specificity pair. *Sensitivity* is the *true positive rate TPR-*the proportion of patients with a diagnosis of AN who test positive (for example, AppEn value lower than the cut-off point), whereas *specificity* is the *true negative rate TNR*-representing the percentage of Control-healthy subjects that are correctly recognized (e.g., AppEn value higher than the cut-off point). *Accuracy* refers to a related measure, quantifying the total number of subjects (patients with AN and control subjects) that are accurately classified. According to the specifications of the ROC functions, they select the optimum threshold as the cut-off point in which the *highest accuracy* (minimal false negative and false positive results) is obtained. This highest accuracy is determined graphically from the ROC curve and is the closest value to the left top point (100% sensitivity, 100% specificity). 

A rough guide to classify the precision of a diagnostic test is related to the area under the ROC curve, AUC. Values ranging from 0.90 to 1 indicate the precision of the diagnostic test to be excellent, good for values between 0.80 and 0.90, fair if the results are in the range 0.70–0.79, poor when the value of the AUC is between 0.60 and 0.69, and bad for AUC values between 0.50 and 0.59. All results regarding the classification performance assessment described above are provided in Section 3.7.

## 3. Results 

### 3.1. Statistical Analysis AppEn and HFD Features

Statistical analysis was performed by SPSS Statistics, version 20.0, SPSS Inc. Before proceeding to ANOVA analysis of the extracted linear and nonlinear features extracted from the EEGs, the Kolmogorov–Smirnov (KS) and Shapiro–Wilk (SW) tests of Normality were used. A value of *p* < 0.05 (two-tailed) was considered statistically significant.

The main findings of this analysis are that almost all extracted features except the Higuchi FD and all syllogisms were found to be not normally distributed. Therefore, nonparametric methods are implemented to detect the discriminating performance of all linear and nonlinear features before proceeding further to their classification capabilities. Tables S1 and S2 (Supplementary Material) present the descriptive statistics of the Grand Mean (across all channels) of the AppEn extracted data per group and per gender, respectively.

In the Kolmogorov–Smirnov (KS) and Shapiro–Wilk (SW) normality tests (Tables S3 and S4, Supplementary Material), we tested the null hypothesis (both per group and per gender) that the data come from a normal distribution process. Our results showed that the mean value of AE for valid syllogisms for the group of controls is not normally distributed (KS = 0.293, df = 28, *p* = 0.000 and SW = 0.743, df = 28, *p* = 0.000), while for the AN group, it is normally distributed (KS = 0.231, df = 12, *p* = 0.078 and SW = 0.884, df = 12, *p* = 0.000). Similar results hold for the mean value of AE for invalid and paradox syllogisms. Therefore, we can conclude that there is not enough evidence that the mean AE values for patients with AN are non-normal, while the opposite holds for controls.

Figures S1 and S2 (Supplementary Material) depict the box plots of the grand mean values (across all channels) of AppEn (AE) to compare their distributions between the two groups and gender. As it is shown, the median lines of the box plot of the AN group (irrespective of syllogism) lie outside of the box plots of controls for each syllogism, an indication that there is likely to be a difference in mean between the two groups. Similar information is provided in Figure S2 but for gender differences. From the Interquartile range (IQR), the AE values of controls are more dispersed than those of AN patients and are more dispersed between females and males. We observe that the number of outliers in the group of controls is larger than that in the AN group. The mean AE values for paradox syllogisms of the AN group are left skewed, while valid and invalid are symmetric, a result that also holds for controls. 

Tables S5 and S6 (Supplementary Material) present the descriptive statistics of the Grand Mean (across all channels) of the HFD extracted data per group and per gender, respectively. We observe that *the average of the Grand Mean of HFD data of patients with AN, in all syllogisms, is larger than that of controls*, enhancing the widely held view that EEGs of patients are more complex than that of controls. Also, females show larger values of average Grand Mean of HFD than males in all syllogisms. (These findings are further supported by the box plots of Figures S3 and S4 in Supplementary Material).

The normality tests of HFD per group and gender (Tables S7 and S8, respectively, Supplementary Material) indicate that the mean values of HFD for both groups and gender are normally distributed (e.g., in the test of data for group and valid syllogism, KS = 0.116, df = 28, *p* = 0.200 for controls and KS = 0.116, df = 12, *p* = 0.200 for AN). Similar results hold for invalid and paradox syllogisms. Thus, an ANOVA can be applied to detect differences between the mean values of HFD of the two groups (see Section 3.3).

### 3.2. Statistical and Correlational Results of Behavioral Data

#### 3.2.1. Correctness: 

Independent samples *t*-tests were performed to detect differences in correctness between the two groups. For the valid case, the tests showed no significant differences in % correctness (correct answers/39 × 100) between the two groups (F = 0.277, *p* = 0.601). Specifically, the average % of correct responses for the healthy controls was 75.3 (+ or −10.9) %, whereas for the AN group was 72.47 (+ or −12.98). Similarly, no significant differences were found between the two groups in the case of invalid (F = 0.014, *p* = 0.905) and of paradox (F = 0.177, *p* = 0.675). (Figure S5 in Supplementary Material shows a box plot of the distribution of % correctness per syllogism and group).

#### 3.2.2. Degree of Confidence (DoC): 

No significant differences in the level of confidence (%) in the responses of the subjects of the two groups are found in valid, invalid, and paradox syllogisms (*p* = 0.198, 0.708, 0.160), respectively) (Figure S6 in Supplementary Material shows the distribution of DoC across syllogisms and groups).

#### 3.2.3. Correlation Analysis of Behavioral and AppEn/Higuchi FD Values

We performed a Spearman ranks bivariate correlation between Behavioral variables and AppEn (Tables S9–S14, Supplementary Material) and HFD (Tables S15–S20, Supplementary Material) extracted features to detect possible linear relationships.

#### 3.2.4. Correlations of Behavioral Data (% Degree of Confidence-DoC and % Correctness) with AppEn and HFD (Spearman’s Rho, Nonparametric)

We observe that (Tables S21–S24, Supplementary Material) in the case of valid syllogism and for patients with AN, the % correctness is positively correlated (ρ = 0.629, *p*-value = 0.028) with % DoC of these subjects, i.e., the higher % of correct answers is associated with higher DoC. 

#### 3.2.5. Independent Samples *t*-Tests of Behavioral Data (% Degree of Confidence, DoC and % Correctness) 

We also performed independent samples *t*-tests to behavioral data % DoC and % Correctness to detect differences between the two groups (Table S25, Supplementary Material). In the valid case, partial significant differences were found in the % DoC (t = 1.615, df = 38, *p* = 0.057) and % Correctness (t = 1.595, df = 38, *p* = 0.059). Their mean values (83.7% and 75.36%, respectively) are higher in healthy controls than in patients with AN (77% and 69%, respectively).

### 3.3. MANOVA of Extracted Higuchi Fractal Dimension (HFD) Feature

As we observe in the following two tables, from the multivariate tests (Table 4), the Combined Dependent Variable (CDV) formed by all 14 channel values of the Higuchi FD, there is a group effect (i.e., the average of CDV is different in the two groups only for the *valid* syllogism (Pillais Trace value, PTV, 0.573, F = 2.395, *p* = 0.028, partial eta squared, pes = 0.573). The partial eta squared (pes) values are all >0.14 (14%), indicating a statistically significant (large) group effect (pes reflects the proportion of variance in the CDV that is accounted for by the factor group. From the output of Levene’s test (not shown here), *p* > 0.05 for all channels, so for most of the channels, there is equality in variance, a result further enhancing the reliability of the analysis. 

The tests between subjects (i.e., channels in our case) effects (Table 5), show that the two groups, Control and AN, differ significantly in terms of the mean HFD at channels AF3 (*p* = 0.018), F7 (*p* = 0.0540), F3 (*p* = 0.077), O1 (*p* = 0.024) O2 (*p* = 0.031), F4(*p* = 0.007) and AF4(*p* = 0.001), if (alpha-value or *p*-value) is not Bonferroni adjusted (*p* < 0.0035, since Bonferroni adjusted alpha level = 0.05/14 channels, due to multiple comparisons). Only *channel AF4 (Anterio-Frontal, right), in the valid syllogism, satisfies this condition*, while the rest of the channels have no contribution to the total variance of the CDV in all syllogisms (all have *p* > 0.0035). Also, from partial eta squared values, it is shown that the largest amount of variance is accounted for by the “group” factor at channel AF4 in the valid case (~27%).

Similar results can be obtained by using separate One-Way ANOVA for each syllogism, as shown in Table 6, Table 7 and Table 8. The main observations are that (a) Ch1, Ch2, Ch4, Ch7, and Ch12 to Ch14 are the brain regions that present significant differences in HFD estimates in the valid case, and only in Ch14 in the paradox case, (b) consistently, the mean HFD values in the majority of patients with AN and in almost all channels, are higher in comparison to the healthy controls, enhancing further the widely held view that patients exhibit higher level of complexity in their recorded EEGs. Table 9 shows the results obtained by the One-Way ANOVA of Grand mean values of HFD across all 14 channels.

### 3.4. Nonparametric Tests of Extracted AppEn Feature

Due to the not normally distributed data of the AppEn feature data across subjects, nonparametric tests were performed to detect differences between the two groups. Initially, the null hypothesis was that the medians, range, and distribution of the AppEn values at each channel and for each group were the same across the group variable. Various tests such as the Independent Samples Median Test (ISMT), Kruskal–Wallis (KW), and Kolmogorov–Smirnov (KS) were employed, using the SPSS ver.20 statistical toolbox. As an example, the rejection of the null for the medians at channel 3 (ch_3, F3), in the case of valid syllogism, was attained via the ISMT. In the table below, we list the results of the KW nonparametric tests performed for each syllogism (the complete SPSS output tables are provided in the Supplementary Material, Tables S26–S28). 

From the tables, in the *valid case,* statistically significant differences in the AppEn values between the two groups are exhibited in F3, T7, P7, O1, O2, P8, T8, FC6, F4, and F8, while in the *invalid case,* this happened in *all* channels. In paradox syllogism, F3, FC5, T7, P7, O1, O2, P8, T8, F4 and F8. 

From Table 10 we observe that *invalid* syllogism produces cognitive loads that activate all 14 brain regions, while *valid* and *paradox* 10 regions. (The results of the non-parametric Kruskal–Wallis test for the Grand mean values of AppEn are shown in Table S29, Supplementary Material). For the Grand mean AppEn values, in the *valid* syllogism, there was a *significant effect* of the *group* variable (Controls vs. AN) (χ^2^ = 8.028, df = 1, *p* < 0.05), also in the *invalid* (χ^2^ = 15.4, df = 1, *p* < 0.05), as well as in the *paradox* (χ^2^ = 6.9, df = 1, *p* < 0.05). 

**Figure 5:**
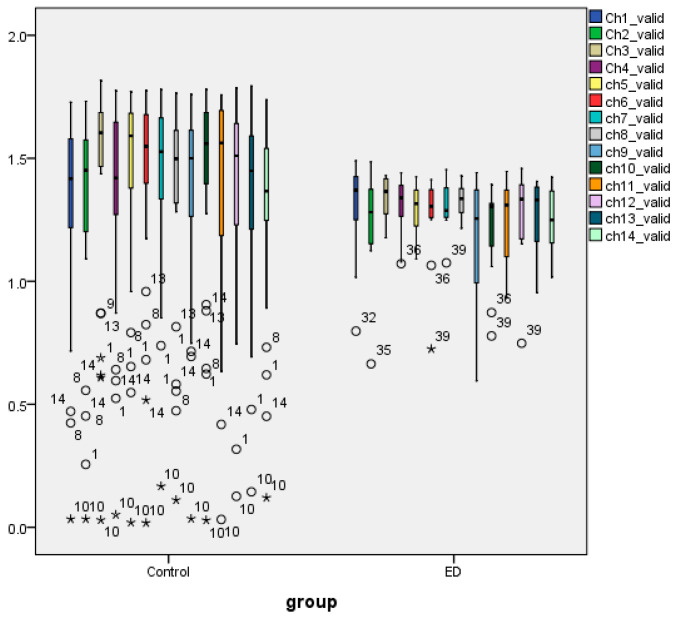
Box plot of AppEn values for the two groups across 14 channels for the valid syllogism. “O,*” symbolize the extreme values or outliers.

Figure 5 presents the box plots of AppEn data for all channels per group, with the pronounced result that in the AN group (ED), the medians in each channel are smaller than in the controls in the case of valid syllogism. An interesting finding *(in conflict with the results given by HFD data)* is also that the medians of the Grand Mean AppEn values in patients with AN are smaller than in controls, reflecting a lower-level complexity in the overall brain activation of patients, compared to that of controls (Figure S7 Supplementary Material).

**Figure 6:**
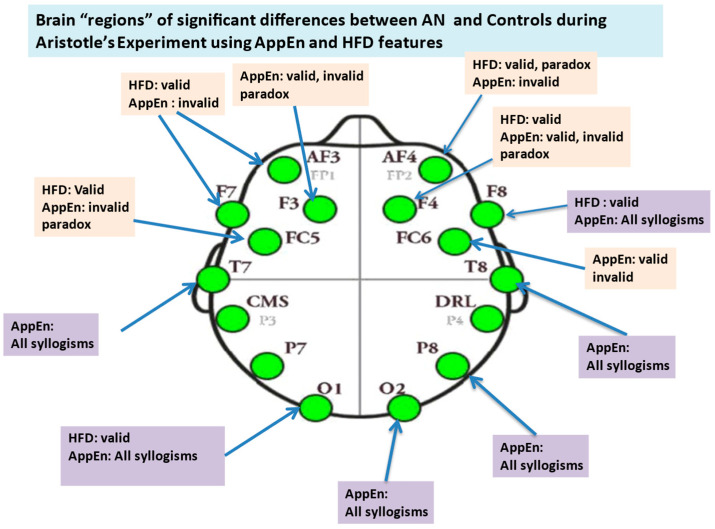
A pictorial presentation of the brain regions of the participants of the two groups that exhibit the highest differences of the HFD and AppEn features for the syllogisms shown.

Figure 6 pictorially summarizes all previous results, providing information on the brain regions on which the subjects of the two groups exhibit significant differences in the values of HFD and AppEn.

### 3.5. Nonparametric Tests of EEG Extracted Power Rhythms Feature

In Tables S30–S41 (Supplementary Material), we show the results of the Kruskal–Wallis nonparametric tests for the extracted, via wavelet analysis, EEG rhythms alpha, beta, delta, and theta, for the valid, invalid, and paradox type of syllogism, to detect any statistically significant differences in mean between the two groups. 

Regarding the *alpha rhythm*, in the case of *valid* syllogism, there is no significant effect of alpha rhythm (all *p*-values or sig. are large), so alpha rhythm does not differ in the two groups, i.e., it cannot discriminate patients with AN from healthy subjects (as shown in Table S30, Supplementary Material). In the *invalid* syllogism, only in channel ch9 (P8) is a significant effect, while in *paradox* syllogism, alpha rhythm has significant effects in channels ch2(F7) (*X*^2^ *=* 3.99, df = 1, *p* = 0.046), ch10(T8)(*X*^2^ *=* 3.976, df = 1, *p* = 0.046), ch11(FC6) (*X*^2^ *=* 4.420, df = 1, *p* = 0.036) and ch14(AF4) (*X*^2^ *=* 4.244, df = 1, *p* = 0.039) (Tables S31 and S32, Supplementary Material).

For the *beta rhythm* (Tables S33–S35, Supplementary Material), it has significant effect on ch1(AF3), ch3(F3), ch4(FC5) and ch11(FC6) in the *valid* syllogism, Ch1(AF3) and ch6(P7) in the *invalid* case, and ch8(O2) in *paradox*. *Delta rhythm* (Tables S36–S38, Supplementary Material) has no significant effect on all channels for almost all syllogisms except ch5(T7) in the *paradox* case. 

*Theta rhythm* (Tables S39–S41, Supplementary Material) has a significant effect, for *invalid* syllogism, on ch2(F7), ch11(FC6), and ch14(AF4), and no significant effects at all for the *valid* and *paradox* cases. 

Table 11 shows the brain regions (channels) at which there are significant differences in the values of EEG-specific bands (rhythms) between the healthy controls and patients with AN. We observe that significant differences in *alpha (α)* band between the two groups are located at frontal-temporal, frontal-central, anterior-frontal, and right-temporal regions in the case of *paradox* syllogisms and right parietal in *invalid* syllogism. Significant differences in *Beta (β)* are at all frontal regions in the *valid* syllogism, at the left anterior-frontal and left parietal invalid cases, and at right occipital in paradox cases. Differences in *delta (δ)* band are concentrated only at the left temporal in the *paradox* case. Finally, frontal-temporal, frontal-central, and anterior-frontal are the brain regions of the two groups that exhibit significant differences in *theta band (θ)* when the participants face invalid syllogisms. 

**Figure 7:**
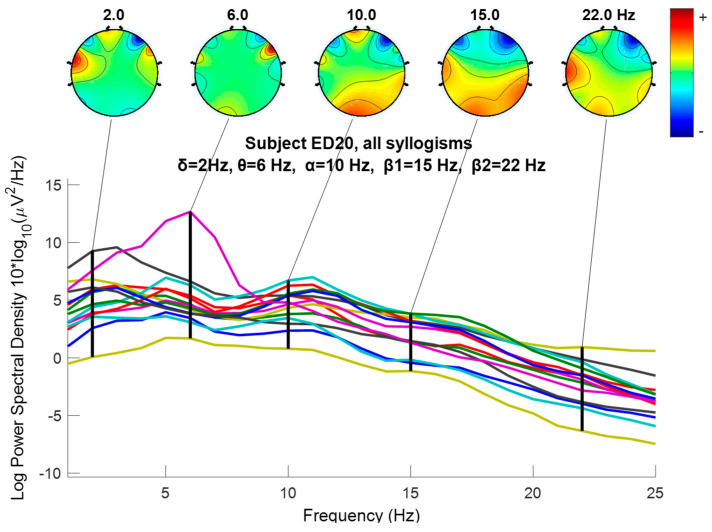
Channel spectra and topographical map: distribution of specific EEG spectra bands (δ, α, θ, β1, β2) on the scalp of a patient with AN (ED20), chosen arbitrarily. The EEG signals in 14 channels contain responses to all syllogisms and contain 50% of the total 334,464 data points (total 2613 s), i.e., 1306 sec (sampling rate *f_s_* = 128 Hz). The curves with different colors correspond to 14 channels.

Figure 7 shows the plot of the channel spectra and associated topographical maps for a representative patient with AN (#ED20), chosen arbitrarily. Each colored trace represents the spectrum of the brain activity of one EEG channel (electrode). The leftmost scalp map shows the scalp distribution of the power at 2.0 Hz (δ band), which in this data is concentrated strongly on the F7 (temporal, left) and gradually, with less intensity, on the F7 (Frontal-temporal, left), as well as on F8 (Frontal-temporal), restricted on a smaller region. In the same region and with similar intensity, band θ (6 Hz) activity is concentrated. Band α (10 Hz) activity is pronounced in a broader area, consisting of O1, O2 (Occipital regions), and P8 (Parietal, right), as well as on a smaller frontal region between anterior-frontal, left and right, AF3 and AF4, with less intensity. Thus, *band α activity dominates the occipital and right parietal regions.* Also, the entire lower scalp hemisphere, extended from T7 (strongly) to O1, O2, and P8 (strongly) regions, is “heavily” dominated by band β1 (15Hz). The temporal left region, T7, is activated strongly by the β2 band, while O1 and P8 are also β2 activated, but with lesser intensity.

In conclusion, and only for this randomly chosen patient with AN, bands α and β1 (10 and 15 Hz, respectively) activate strongly larger parts of the brain than other bands when the patient is exposed to all Aristotle’s syllogisms, with emphasis on occipital and parietal (left and right) regions. Taking the average of the spectral curves of these bands, of all subjects, different results of the average topographic map are expected for each group. 

**Figure 8:**
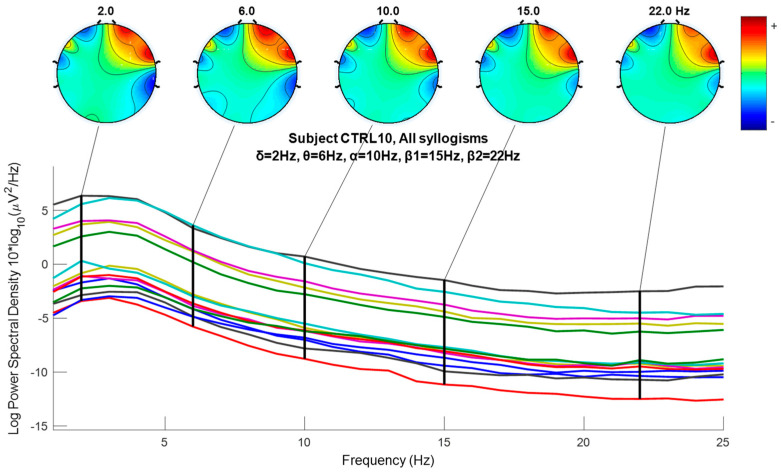
Channel spectra and topographical map: distribution of specific EEG spectra bands (δ, α, θ, β1, β2) on the scalp of a Healthy Control subject (CTRL10), chosen arbitrarily. The EEG signals in 14 channels contain responses to all syllogisms containing 50% of the total 341,888 data points (total 2671 sec), i.e., 1335 s (sampling rate *f_s_* = 128 Hz). The curves with different colors correspond to 14 channels.

Figure 8 depicts the distribution of EEG power of specific spectra (maps) for the healthy control subject #10 for all Aristotle’s syllogisms. In general, this figure, compared to the previous one for a patient with AN, shows emphatically that the brain’s activity in this case is concentrated on smaller regions, primarily in the part extended between AF4 (Frontal-temporal, right), F4 *Frontal, right) and F8 (Frontal-temporal, right). No concentration of activity of any specific spectra is exhibited on the left and occipital regions, a very interesting observation. This finding, however, concerns only the healthy subject under analysis, and it cannot be generalized at this stage of our analysis. However, the difference between the two figures is apparent, i.e., *the specific bands activate more brain regions in the case of patients with AN than in the case of healthy control,* a result that is further enhanced by the *AppEn and HFD complexity findings* that are *higher in the case of patients with AN,* when they face cognitive tasks of great difficulty such as syllogisms in the Aristotle’s experiment.

**Figure 9:**
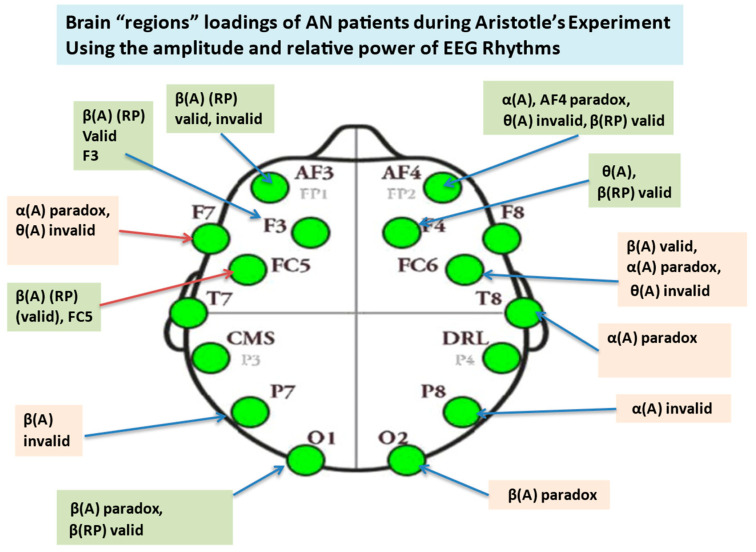
Results of amplitude and relative power values of EEG rhythms (from nonparametric tests) indicate the brain regions that were mostly activated during Aristotle’s experiment and differ significantly between the two groups.

The results from non-parametric tests of amplitude and relative power values of EEG rhythms are placed on a scalp map, as shown in Figure 9, to pictorially emphasize the brain regions that are mostly activated when Aristotle’s experiment is performed. A classification scheme of high accuracy can also be obtained by HFD-channels dyads, as shown in the figure. If instead of HFD, we consider AppEn on a similar figure (not depicted here), this feature will “occupy” all regions in the case of invalid syllogism.

### 3.6. Nonparametric Tests of EEG Extracted Rhythms–Relative Power 

As we have already mentioned before, in Section 2.6, relative power is associated with the question: Who is in charge here? RP can be realized as the *power in one frequency band compared to all other bands* or the *distributed total amount of power* at each brain location. It is used to determine whether a specific frequency (e.g., alpha) is *overpowering other vital brain frequencies* or if the power is *low*.

The variability of values is different between the four syllogism-induced workloads, which were explored by statistical evaluation of the selected relative power of EEG rhythms (features). We used one-way ANOVA for normally distributed features and Kruskall-Wallis (KW) nonparametric tests for not normally distributed variables of the relative power of EEG rhythms, individually for each syllogism and group, presenting statistical significance at *p*-values < 0.05 (95% confidence interval). ANOVA and KW tests were performed on both separate channel levels and on the level of *Average Grand Mean of Relative Power, AGMRP* (average of all 14 Grand mean values, in each channel, for each subject).

#### 3.6.1. Alpha Relative Power at Channels Level, Valid Syllogism

The relative power values at channels ch1, ch2, ch11 and ch13 were found to be (by Kolmogorov-Smirnov test, KS) not normal, for both patients with AN and controls. An ANOVA of these channels reveals that there are no significant effects of the group variable (F_(1,39)_ = 0.007, *p* = 0.870, F_(1,39)_ = 0.035, *p* = 0.365), F_(1,39)_ = 0.595, *p* = 0.445, F_(1,39)_ = 0.346, *p* = 0.560, for the channels mentioned respectively. The relative power at the rest of the channels is not normally distributed, so a KW test was performed. The test (results are given in supplementary material) also showed no significant effects of the group variable. Thus, the values of alpha relative power at all channels for valid syllogism do not differ significantly between the two groups. The average of Grand Mean Values (AGMV) of alpha power was found to be not normal. No significant effects of the group variable were found, using the KW test, for this syllogism (X^2^ = 1.075, df = 1, *p* = 0.300). 

#### 3.6.2. Alpha Relative Power for Invalid Syllogism

KS test showed that alpha relative power at ch6 and ch9 of the AN (ED) group is not normal (KS = 0.247, df = 12, *p* = 0.042, for ch6 and KS = 0.200, df = 13, *p* = 0.039, for ch9, respectively). ANOVA for the rest of the normal variables (corresponding to all other channels) indicated no significant effect on the group. Also, the nonparametric KW test for variables corresponding to ch6 and ch9 also indicated no significant effect. Regarding the average grand mean of the alpha relative power for this syllogism, the KS test showed that both variables for patients with AN and controls are normal. The ANOVA effect showed no significant effect (F = 0.030, *p* = 0.864). 

#### 3.6.3. Alpha Relative Power for Paradox Syllogism

Alpha RP at ch4, ch5, ch6, ch7, and ch11 were found normally distributed. Their ANOVA showed no significant effect on the group. No significant effect of the group was also found (by the KW test) in the alpha relative power at the rest of the channels (not normally distributed). For the paradox, the average of Grand Means of this power was found to be not normally distributed for controls and normally for patients with AN. Therefore, the KW test indicated no significant effect (X^2^ = 0.380, df = 1, *p* = 0.538).

#### 3.6.4. Beta Relative Power, Valid Syllogism

Due to the non-normality of both series in the Controls-AN pairs, a KW test was applied to the beta relative power variables at ch1, ch3, ch4, ch7, ch12, and ch14. The results shown below (Table 12) indicated a significant effect of the group variable (Beta relative power feature discriminates effectively between the two groups). Also, the average of Beta RP Grand Means values in the case of *valid* syllogism indicated significant effects of *group* variable (X^2^ = 3.957, df = 1, *p* = 0.047), and in fact, its value for patients with AN (22.23%) is larger than its value for controls (17.65%), indicating a higher level of complexity of the patients’ “total” brain activation.

#### 3.6.5. Beta Relative Power, Invalid and Paradox Syllogisms

KW tests applied to both these cases (due to not normally distributed variables) showed no significant effects at either individual channels or brain “Total” activation (average grand mean values) levels. 

#### 3.6.6. Gamma Relative Power 

For the relative power of this rhythm, only at channel 14 (AF4), and for *valid* syllogism, there was a significant effect (KW test, X^2^ = 4.298, df = 1, *p* = 0.038), as well as at channel 10 (T8) for *paradox* syllogism (X^2^ = 5.15, df = 1, *p* = 0.023). No significant effect was found for all syllogisms’ average Grand Mean values.

#### 3.6.7. Delta Relative Power

No significant group effects were found for all channels for all syllogisms. For all syllogisms, no significant group effects were found for the average Delta relative power variables.

#### 3.6.8. Theta Relative Power

The KW tests for the relative power of this rhythm, for the valid and invalid syllogisms, indicated that there was a significant effect at the channels shown in the following tables. In the case of paradox syllogism, no significant effects were found in any channel. 

From Table 13a,b, thus, patients with AN and healthy controls exhibit their highest differences in Theta rhythm relative power in all *frontal brain regions* in the cases of *valid and invalid* syllogisms.

Also, significant effects were found for the average of Grand Mean values (Brain’s “Total activity”) for the *valid* (X^2^ = 4.069, df = 1, *p* = 0.044) syllogisms. 

Table 14a,b provide a summary of the analysis results on both *separate channels* and “*Brain’s total activity*” levels for each syllogism. Bold letters indicate significant group effects (where patients with AN and healthy controls differ significantly).

Considering all the above results on both levels of analysis (on each separate channel and on AGMRP), we note that: 

Based on “mainstream” statistical tools of ANOVA and KW nonparametric tests, no significant group effects were found in all channels for Alpha, Delta, and Gamma relative power; thus, these rhythms cannot detect differences in the brain regions, as well as they are not suitable as inputs to a classification process, for grouping effectively and reliably, AN and healthy controls.

Regarding the “total brain activity”, of patients with AN and healthy controls, based also on ANOVA and KW tests:

- No significant group effects were found for the Alpha AGMRP, in all syllogisms;
- *Significant group effects* were found for the Beta AGMRP, in *valid* syllogisms;
- No significant group effects were found for the Delta AGMRP, in all syllogisms;
- No significant group effects were found for the Gamma AGMRP, in all syllogisms;
- *Significant group effects* were found for the Theta AGMRP, in *valid* syllogisms.

**Figure 10:**
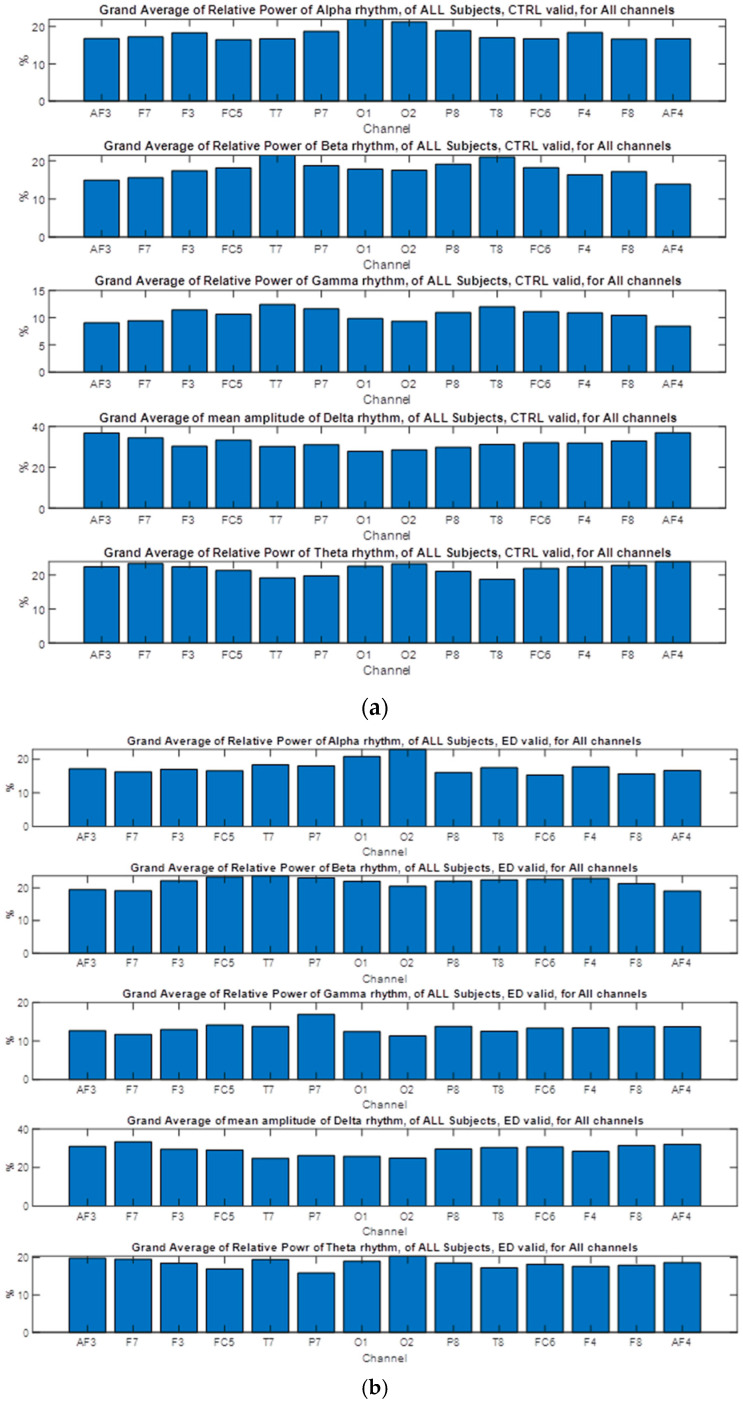
(**a**)**:** Grand average of the relative power of all rhythms, valid case, for healthy controls. (**b**): Grand average of the relative power of all rhythms, valid case, for AN (ED) patients.

According to Table 12, for the valid case, Figure 10a,b, indicate that the beta relative power values differ significantly at channels AF3, F3, FC5, O1, and F4. For example, of controls at O1 and O2 occipital channels, the bars indicating % of beta relative power are smaller than the bars in the AN (ED) group, indicating higher complexity at the occipital regions of patients with AN. In general, the complexity of patients is higher than that of controls in all channels shown in Table 12 and Table 13 and pictorially in Figure 10a,b. 

### 3.7. Results of Classification

#### 3.7.1. Classification Based on Higuchi and AppEn Features

Table 15a,b provides summary information of the classification results, using a variety of classification models (classifiers) and AppEn and Higuchi FD as input descriptive features, and “group” factor (Control, AN) as the target feature, with and without Principal Components (PC), respectively. Table 16a,b (Section 3.7.2) provides the same information but for the amplitude of EEG rhythms. The tables show the accuracy in %, the Area Under (ROC) Curve, AUC, and the OOPROC (sensitivity, specificity) coordinates. A first observation is that PC does not improve the classification and, in some cases, deteriorates it. 

The triad “*feature-syllogism-classifier*” constitutes all possible combinations examined in the present research experiment, used to detect the best classification results. Therefore, the *best classification* of subjects with AN is (a) “AppEn-Invalid-Enseble BT”, presenting an accuracy of 83.3% (see Section 2.8). (b) “Alpha amplitude-Valid-SVM”, achieving a similar accuracy of 83.3%.

#### 3.7.2. Classification Based on the Extracted Features of Amplitude of EEG Rhythms

Table 16a,b provides a summary information of the classification results, using a variety of classification models (classifiers) and the amplitude of EEG rhythms as input descriptive features and “group” factor (Control, AN) as the target feature, with and without Principal Components (PC). 

We observe that the highest accuracy of 83.3% is achieved by using both AppEn and Alpha rhythm input features for the invalid and valid syllogism, respectively, with Ensemble Bagged Trees as the best classifier for the first triad and SVM for the second one. 

We also point out that the accuracy attained by the Alpha rhythm feature and valid syllogism is higher than that achieved by using Higuch FD, indicating the superiority of the information-based feature of AppEn. The second higher accuracy, 75.6%, is achieved by an Ensemble Bagged tree, using the power of the Alpha (Rhythm) Relative Power feature for the valid case. We recall that alpha rhythm was found from Kruskal–Wallis nonparametric tests (the equivalent to ANOVA for not normal data tests) (see Section 3.5) to provide statistically significant values in channel ch9(P8) (invalid case) and ch2(F7), ch10(T8), ch11(FC6) and ch14(AF4) (paradox case). This indicates that the “linear” nonparametric tests are less capable of detecting differences between the two groups in the cases of valid syllogism, than the powerful classification tool of Ensemble BT, which detects differences with a 75.6% accuracy. The Kruskal-Wallis nonparametric tests and the Ensemble BT classifier indicate strongly that the dyad “valid syllogism-alpha rhythm” effectively detects the differences between patients with AN and controls. 

Regarding the good discriminatory performance of the triad “Theta-Invalid-KNN”, it is in accordance with the findings of the Kruskal–Wallis nonparametric tests, in which ch2(F7), ch11(FC6), ch14(AF4) for the invalid case, show significant values (effects). A very interesting observation is that ch2(F7), ch11(FC6), and ch14(AF4) show significant values in alpha and theta rhythms for paradox and invalid cases, respectively. Therefore, the differences in the level of activation of the brain of the subjects, at these regions, of the two groups (Control and AN) are high when they face the loads exerted by previously mentioned Aristotelian syllogisms. 

**Figure 11:**
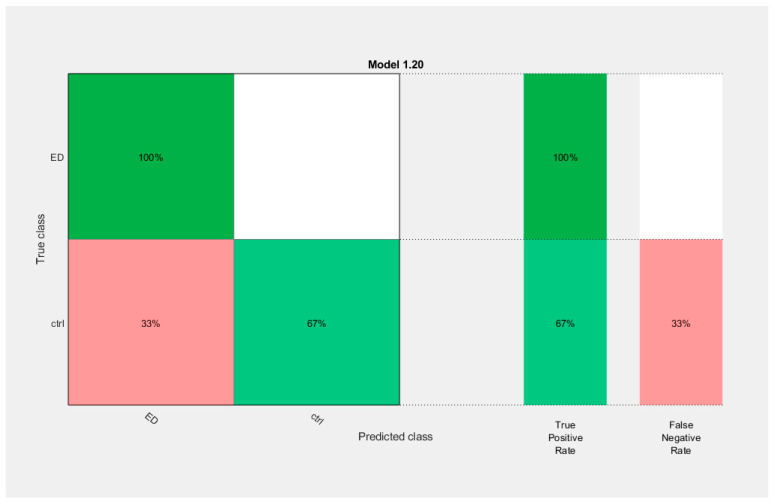
Confusion matrix for assessment of the best-found model *(Ensemble Bagged Trees*), for AppEn Rhythm, Invalid syllogism. The classification performance accuracy of the model is 83.3%.

From the above results, we opted to concentrate on the AppEn and Theta rhythm features that generate the highest accuracies in accurately classifying new information regarding patients with AN. In Figure 11, the *confusion matrix (CM)* is shown, a tool for assessing the performance of the “best” found model Ensemble BT, attaining an accuracy of 83.3%, using AppEn as the input descriptive features for the invalid syllogism. Other models have been found for the rest of the syllogism, attaining, however, less accuracy. 

The top row of the *confusion matrix* shows all subjects with true class ED (patients with AN). The columns show the predicted classes. In the top row, 100% of the subjects with AN are correctly classified, so 100% is the *true positive rate (TPR)* for correctly classified subjects in this class, shown in the green cell in the True Positive Rate column. No other subjects in the AN row are misclassified, i.e., incorrectly classified as Control. So, 0% is the *False Negative Rate (FNR)* for incorrectly classified subjects in this group, shown in the red cell (white here, because of zero) in the FNR column. Similarly, 33% of the true class ctrl are incorrectly classified as AN, while 67% are correctly classified. Thus, TPR = 67% and FNR = 33% in this case.

**Figure 12:**
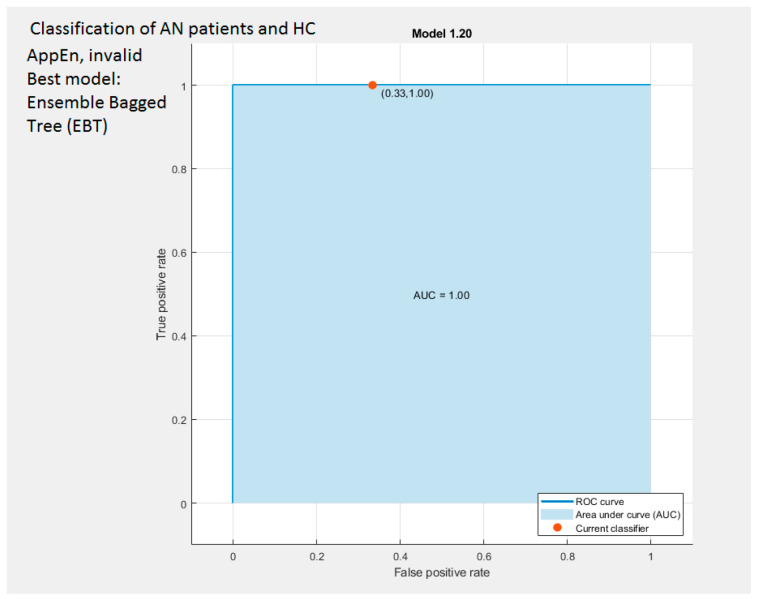
Receiver Operating Curve (ROC) was used to assess the performance of the “best” model found: Ensemble BT, with AppEn feature, for the invalid syllogism. Here, AN is considered to be a positive class.

The Receiver Operating Curve (ROC) in Figure 12 shows true and false rates, using AppEn values as input features again for the invalid syllogism, with AUC = 1.00. The AN group is chosen here (arbitrarily) to be the positive class and HC the negative one. The red marker on the plot shows the performance of the best classifier Ensemble BT, i.e., the (FPR, TPR) = (0.33, 1.00) coordinates. Since FPR = 0.33, the Ensemble BT classifier incorrectly assigns 0.33% of the observations to the AN class. Here also, TPR = 1.00, i.e., the model correctly assigns 100% of the observations to the AN class. This is a perfect result with no misclassified points and is a right angle to the top left of the plot. A poor result that is no better than random is a line at 45 degrees. The *Area Under Curve (AUC)* number, here AUC = 1.00, is a *measure of the overall quality* of the classifier. Larger AUC values indicate better classifier performance. The fifth column of Table 15 and Table 16 shows AUC values for all classifiers used in this paper. The result just found is in accordance with the CM for the AN group (positive class).

#### 3.7.3. Classification Results of Relative Power of EEG Rhythms

In the classification of relative power, the adopted feature selection method was the default one of MATLAB’s vers. 2018b, classification learner app. Table 17 below summarizes the results of the classification of healthy subjects and patients with AN based on the feature EEG rhythms relative power, i.e., shows the best-found triads “*EEG rhythms relative power-syllogism-classifier*”.

By comparing the results of the classification given in Table 17, based on the relative power of EEG rhythms, with the results of ANOVA and KW tests, given in Table 12, Table 13 and Table 14, we observe that they are not consistent. In fact, although ANOVA and KW tests showed no significant group effects for the alpha relative power on both levels of analysis (separate channels and “total brain activity”), the classification instead has revealed that alpha rhythm, combined with some classifiers, provides classification models with very good accuracy. More specifically, the triad “alpha RP-valid-fine tree”, has an accuracy of 75.6%, the triad “alpha RP-invalid-SVM”, an accuracy of 70%, etc. This inconsistency in the results of these two methods can be explained as follows: ANOVA estimates the mean value of alpha RP of controls and AN by terms of variance of the confidence interval, while the feature selection method, e.g., of a SVM scheme implemented, determines this feature’s prominence by weights deriving from the internal structure of SVM, mapping feature vectors in multidimensional space and estimating maximum hyperplane deviations [121]. Therefore, the two approaches work on a complementary basis; in fact, ANOVA and KW tests are used as the first “rough” tool to detect the extracted feature’s effectiveness in separating the subjects of the two groups. 

## 4. Discussion 

### 4.1. The findings from Our Study

The most important finding of this study is that the extraction of non-linear features linked to the complexity of EEG signals can lead to high and potentially useful tools for discrimination or separation of EEGs recorded from healthy control subjects and patients diagnosed with anorexia nervosa. We propose that Aristotelian syllogisms (constructs based on Aristotle’s logic designs) used in our experiment form a suitable “background” in which such features operate efficiently towards this end. These syllogisms exert demanding cognitive loads on the participants, producing intense activations in brain regions that can be detected via “sophisticated” tools. In this context, Wang et al. [122], in their meta-analysis of previous data related to neural substrates for deductive reasoning, highlighted the need for applying new techniques to explore how different brain regions are activated during this process.

In this work, we have used two features extracted from EEGs, originating from the nonlinear dynamical systems theory, Higuchi’s Fractal Dimension (HFD) and approximate entropy (AppEn), as well as the “mainstream” tool of EEG-specific frequency bands (alpha, beta, delta, and theta). Consequently, we combined these features with each of the valid, invalid, and paradox syllogisms, to form suitable dyads of “features-syllogism” used as input to various machine learning classification techniques. These inputs are used further in generating the triad “*feature-syllogism-classifier*”, exhibiting the highest classification accuracy. In other words, properly selecting these triads makes an important classification problem more tractable. To the best of our knowledge, we were the first to apply this type of triad on the particular classification task of discriminating healthy controls and patients with AN based on EEGs. We recapitulate at this point the most important findings of this paper: 

The mean HFD of healthy controls (valid case) is less than the mean HFD of patients with AN (see Table 18), i.e., on average, *the complexity in the brain activity of subjects with AN is higher* (in accordance with other results in the literature, that patients exhibit higher complexity). In the case of invalid syllogisms, there is a significant effect of the group variable (AN, Controls) for the AppEn. Actually, the mean AppEn of Controls (1.482) is larger than that of AN (1.210) (F_(1,38)_ = 14.512, *p* = 0.00). This indicates a significantly higher level of complexity of the controls’ average brain activity. Furthermore, the mean AppEn of healthy subjects in the invalid case is higher than the mean AppEn when they face valid syllogisms. Conversely, the mean AppEn of patients with AN, when they face invalid syllogism, is lower (*an opposite result than in the valid case*). In other words, when healthy controls face invalid syllogisms, their EEG signals show higher AppEn values than when they face valid syllogisms. On the contrary, patients with AN present EEG signals with lower values of AppEn (hence with higher predictability) when they face invalid syllogisms, in comparison with the condition of the valid type of syllogism. We may conclude, therefore, that healthy controls and patients with AN present lower values of AppEn in relation to the different types of syllogisms presented (valid and invalid syllogisms, respectively).

We observe that the average of the Grand Mean of HFD data for patients with AN, in all syllogisms, is larger than that of healthy subjects, enhancing the widely held view that patients’ EEGs are more complex than that of controls. However, the opposite occurs for the Grand Mean of AppEn, where in all syllogisms, the AppEn of patients with AN is smaller than that of healthy controls. This seemingly conflicting result is explained in Section 2.5.1. The signals of patients with AN are, in fact, fractals but also predictable with not much irregularity since AppEn is low despite the high fractal dimension (higher HFD). Table 18 facilitates the clarification of seemingly conflicting results comparing HFD and AppEn. 

Also, females show larger values of average Grand Mean of HFD than males in all syllogisms.

Regarding the descriptive statistics and correlations of behavioral data, no significant differences were found in the % of correct answers given by healthy subjects and patients with AN and in the level of confidence (%) for the given responses, in valid, invalid, and paradox syllogisms. 

The findings obtained from the correlation of behavioral data (*emotional intensity*, *emotional control*, *mood****,*** and *number of correct answers*) with mean values (across all subjects) of AppEn and HFD for all syllogisms showed that: 

For healthy controls, the only significant positive correlation (0.421, *p* = 0.026) was found between emotional control and mean AppEn in the case of *paradox* syllogism.

For patients with AN, the only significant and also positive correlation (0.635, *p* = 0.027) was found between emotional control and mean AppEn in the case of *invalid* syllogism. 

No significant correlations were found between mean HFD and behavioral data for healthy controls in all syllogisms.

For patients with AN, a significant and negative correlation was found between emotional intensity and mean HFD (−0.588, *p* = 0.045) in the *invalid* case. Additionally, (partial) significant and negative correlation was found for patients with AN between emotional control and mean HFD (−0.574, *p* = 0.051) in the *paradox* case. 

Thus, emotional control is found to be sensitive to the mean complexity of the EEGs, measured by AppEn (positively correlated), of both healthy participants and patients with AN, for paradox and invalid cases, respectively. Higher levels of complexity of their EEGs “generated” by the cognitive loads exerted by these two types of syllogisms reflect higher levels of emotional control of both healthy participants and patients. On the contrary, emotional control and intensity in patients with AN were found to be negatively correlated with mean HFD, again for paradox and invalid cases, respectively. In this case, higher levels of complexity of their EEGs “generated” by the cognitive loads exerted by these two types of syllogisms correlate with lower levels of emotional control and emotional intensity of AN patients. 

Therefore, taking into consideration the conflicting results obtained from these two measures (AppEn, HFD), we may conclude that, at this point, the failure of HFD in relation to AppEn emerges again since (as we have previously shown) EEG signals of AN patients are indeed fractal, but at the same time also predictable, with low levels of irregularity (low values of AppEn). Consequently, we can justify the lower level of emotional control and intensity found in patients with AN despite the higher complexity of their EEGs as measured by the HFD measure. 

In the case of *valid* syllogism and for patients with AN, the % correctness is *positively correlated* with the % DoC of these subjects, i.e., the higher % of correct answers is associated with higher DoC. Regarding differences in the mean of % DoC and % Correctness between the two groups, partial significant differences were found in the valid case. Their mean values were found to be *higher* in healthy controls than in patients with AN. 

From the MANOVA of HFD, for the Combined Dependent Variable (CDV) formed by all 14 channel values of this measure, there is a *group* effect (i.e., the average of CDV is different in the two groups only for the *valid* syllogism.

Also, from MANOVA, healthy controls and patients with AN *differ significantly* in the *valid* syllogism in terms of the mean HFD at channels AF3, F7, F3 (*frontal left*), O1, O2 (*occipital*), F4 and AF4 (*frontal right*) (not Bonferroni adjusted) and only at channel AF4 (Anterio-Frontal, right), in the valid syllogism, when Bonferroni condition is satisfied. 

MANOVA’s main findings also include: a) AF3, F7, FC5, T7, O1, and F4 to AF4 are the brain regions that present *significant differences* in HFD estimates, in the *valid* case, and only in AF14 in the *paradox* case, b) consistently, the *mean HFD values in the majority of patients with AN and in almost all channels, are higher compared to healthy controls,* enhancing further the widely held view that patients exhibit higher level of complexity in their recorded EEGs. 

Regarding the AppEn results, for the Grand mean AppEn values, there was a *significant effect of the group* variable (differences between the two groups) in all syllogisms. 

Also, for AppEn, in the *valid* case, statistically significant differences between the two groups are exhibited in F3, T7, P7, O1, O2, P8, T8, FC6, F4, and F8, while in the *invalid* case, this happened in all channels. In paradox syllogism, F3, FC5, T7, P7, O1, O2, P8, T8, F4 and F8. Also, we observe that *invalid* syllogism produces cognitive loads that activate all 14 brain regions, while *valid* and *paradox* 10 regions. This result further enhances our view that AppEn is a more effective measure than HFD since it detects, on average, a larger number of activated brain regions for most of the syllogisms.

### 4.2. The Contribution of Our Findings in the Existing Literature

Regarding the *Rhythms* results, as shown and commented on Table 11, we point out here that our results are in accordance with the findings in other works, from which the most relevant to us is the paper by Hatch et al. [80], although it should be noted that the sample of patients with AN in that case consisted of adolescents. According to Demos [123] and Blume et al. [124], oscillations in *beta* (β) band are linked to conscious precision, strong focus, and enhanced ability in problem-solving. We also compare our results with those described in a review paper by Blume et al. [124] and the work of Leonard et al. [125]. In the last paper, EEG was derived at three stages, at a 1-min resting state, “eyes open” before, during, and after a test meal. No differences were found in δ, θ, α, and β power between the three conditions and, in comparison, between a group of patients with AN and healthy controls. Therefore, the test-meal condition failed in discriminating the subjects of the two groups, while in our work, the use of Aristotle’s syllogisms proved to be an efficient-suitable background towards this end. Increased beta activity (in terms of frequency analysis) in fronto-temporal and occipito-temporo-parietal areas was reported by Hilui et al. [126] in their review of Binge-purge eating disorders (BP-ED), a finding considered by the authors as indicative of dysfunctional brain network in individuals with this type of ED. Furthermore, a recent study of deductive inferences (logically valid and invalid tasks with the same content) in healthy participants showed two distinct beta-2 (*β2*, 19–30Hz) activations in logically valid inferences in left temporal and central areas, proposing that the specific cortical features found could reflect a distinct neural process for this type of inferences [127].

The results from our study are consistent with previous AN studies reporting reduced alpha [77,79], as well as abnormalities in the beta band in AN [77]. In the paper of Hatch et al. [80], it was found that in an “eyes open” condition, patients with AN (underweight) exhibited significantly *lower alpha* power relative to controls in the frontal, central, and temporal brain regions. Patients with AN also showed significantly *higher beta* in frontal regions than controls. More specifically, even though more demanding cognitive tasks now replace the “eyes open” condition, we observe (see Table 11) that significant differences in alpha band values between the two groups are documented in frontal and temporal regions in paradox and in the parietal region in the invalid case. Thus, a reduced alpha band in patients with AN, compared to controls, is observed in frontal and temporal regions in the case of paradox (of our experiment) and in the “eyes open” condition of the work by Hatch et al. [80]. The invalid case adds new information: a reduced alpha band is documented in patients with AN and the parietal region when facing this cognitive task in Aristotle’s experiment. However, a lower relative alpha 1 (8–11 Hz) band in the parietal area in patients with AN has been reported in the past by Toth et al. [81] in the condition of processing gustatory stimuli. Our results for the beta band capture the same information as in the paper of Hatch et al. [80], showing a higher beta band in frontal regions (valid and invalid cases), but add new information that a higher beta band also occurs in occipital (paradox) and left parietal (invalid case) in patients with AN. Therefore, the main contribution of our work is that the Aristotelian cognitive tasks exert more loads on the participants, activating more brain regions as reflected by significant differences of alpha and beta, delta, and theta bands than in the case of more “naïve” tasks [80]. We emphasize here also that in our work, the left temporal region for the delta band in paradox, as well as frontal (in invalid) regions for theta band, showed significant differences, enhancing further the value of Aristotelian cognitive tasks, in detecting more accurately and reliably all brain regions activated that cannot be detected by using more naïve conditions. 

We recall at this point that brain abnormalities in the frontal region, in both alpha and beta bands are consistent with the *executive function deficits* found in AN, which, according to different authors [80,128,129], are contingent on *prefrontal and frontal brain functioning*. In addition to the above-mentioned subcortical disturbances, fMRI works in AN have shown alterations to exist in prefrontal activity in AN relative to controls [130]. Alpha is considered to manifest “relaxed wakefulness”, while beta “alert attentiveness” [80,131]. The reduced alpha/increased beta pattern has been reported to reveal a total increased cortical activation [132]. 

Regarding changes in theta (θ) bands in healthy individuals and patients with AN, we first recall that performance in inhibition tasks, executive functioning, and working memory are linked to theta rhythms [123,124]. Theta band’s activity is considered not to be related to appetitive learning in Binger-Eating-Disorder (BED) and Bulimia Nervosa (BN). When subjects with normal weight were compared to those with obesity, the last group was found to exhibit reduced theta (θ) power in frontal regions, indicating difficulties in executive functioning. Our results showed that theta band’s values in healthy subjects and patients with AN are significantly different in the *invalid* cases, and more specifically, *theta values* are *lower* in patients with AN, evidence that they face difficulties in dealing with these demanding cognitive loads (i.e., they are deficient in executive functioning). Memory maintenance is also strongly linked to theta activity in frontal regions [133], which, if enhanced, reveals a synchronous activity in these regions when the subjects are involved in cognitively demanding tasks. It seems, therefore, natural to conclude that increased theta activity in frontal regions of healthy subjects and reduced in patients with AN (thus a significant difference), reveals a deficit in executive functions for patients with AN, i.e., they cannot activate frontal regions needed to “manage” adequately demanding cognitive loads. The discrepancies noted with previous studies that reported increased theta activity in patients with AN [82,134] could be attributed to different stimuli/experimental conditions. In the study of Hestad et al. [82], EEG recording was examined in an “eyes-closed” condition, while Spalatro et al. [134] explored basal EEG and reactivity to music stimuli in patients with AN. Additionally, as has been stated before in a review of EEG data in eating disorders by Jáuregui-Lobera [135], the elevated theta power in parietal-occipital areas observed in AN during an “eyes-closed” condition is suggested to be a stable characteristic that remains after refeeding [80]. 

Regarding the results of the *relative power* of EEG rhythms, no significant differences were found between patients with AN and healthy controls in the values of alpha, delta, and gamma waves in all syllogisms examined. On the level of analysis of “Total Brain Activity”, no significant differences in the values of the relative power of alpha, delta, and gamma were found between the two groups. In contrast, significant differences were found in beta and theta waves. Thus, the relative power values of beta and theta (in the “Total Brain Activity” level of analysis) are suitable for effective discrimination of the subjects of the two groups but only for the valid syllogism. 

We must note here that in this paper, we used HFD and AppEn, two nonlinear measures that are able to detect differential aspects of the signal under analysis. More specifically, while HFD examines the complexity in the *time domain*, AppEn an information-oriented approach, can characterize the *irregularity* of a signal or its *predictability* (indicated indirectly as complexity changes in a physical sense). Their results are not consistent since HFD showed higher complexity in patients with anorexia nervosa when compared to healthy subjects, while AppEn showed less complexity in patients with AN than in controls. As explained in Section 2.5.1, Higuchi’s Fractal Dimension (HFD) and Approximate Entropy (AppEn) are particularly well matched in a methodological sense [136], showing different sensitivity for the frequency content of the signal. AppEn shows *better performance in the lower frequency band* and HFD *in higher frequencies* of EEG. 

In summary, differences in information processing of patients with AN in respect of the brain EEG activity may become evident by the concomitant use of demanding cognitive tasks (Aristotle’s syllogisms in our experiment) and entropy-oriented features (AppEn in our case). Previous neuropsychological data in AN have shown that cognitive deficits are manifested as core characteristics of the disorder but also can be remediated [137] and consequently formed the basis for further studies in this field. Therefore, the documentation of possible specific information-processing styles in this eating disorder becomes of great importance.

## 5. Conclusions

Compared to the above literature, we believe that our work has sufficient originality, including the combination of two EEG extracted nonlinear features, AppEn and HFD, as well as main EEGs specific frequency bands or rhythms (α,β,δ,θ), with a set of innovative cognitive tasks (Aristotle’s syllogisms), and a variety of classification methods used in machine learning (with and without principal component analysis–PCA) that prove to produce efficient and very accurate classifiers. Additionally, our findings provide further evidence for specific information-processing styles in anorexia nervosa, as documented by the combination of the tools mentioned before. The importance of shedding light on the reasoning process for this eating disorder has significant implications in targeting psychotherapeutic approaches as well as specialized neurocognitive interventions.

## 6. Limitations 

One limitation of our study is the small sample size, especially for the group of patients with AN, since several EEGs were excluded from an initial group of subjects due to many artifacts. In addition, considering that this is the first study in anorexia nervosa in which the combination of specific EEG features with a set of innovative cognitive tasks (Aristotle’s experiment) is applied, the interpretation of the results should be made with caution. The findings from our study need to be replicated by future studies in this field, using larger sample sizes.

Future EEG studies in AN focusing on the reasoning process could help further shed light on the underlying cognitive mechanisms for this population and the concomitant use of innovative cognitive tasks with specific EEG features seems promising in this direction. 

Furthermore, the discrimination between patients with AN and healthy controls via machine learning techniques puts forward the importance of using these features as possible powerful diagnostic tools in other psychiatric disorders.

## Figures and Tables

**Table 1:** Channel index, name, and location [20].

Channel Index	Channel Name	Location
1	AF3	Anterio-frontal, left
2	F7	Frontal-temporal, left
3	F3	Frontal, left
4	FC5	Frontal-central, left
5	T7	Temporal, left
6	P7	Parietal, left
7	01	Occipital, left
8	02	Occipital, right
9	P8	Parietal, right
10	T8	Temporal, right
11	FC6	Frontal-central, right
12	F4	Frontal, right
13	F8	Frontal-temporal, right
14	AF4	Anterio-frontal, right

**Table 2:** Examples of valid, invalid, and paradoxical syllogism.

Syllogism	
Valid	Paradox
All humans are mortal. All Greeks are humans. All Greeks are mortal.	Achilles and the tortoise decided to race. The tortoise started 100 m ahead of Achilles as the latter is 100 times faster than the tortoise. Therefore, when Achilles will have advanced 100 m the tortoise will be 1 m ahead of him. When Achilles covers this 1 m, the tortoise will be 1/100 m ahead of him. When Achilles covers this 1/100 m the tortoise will be 1/10,000 m ahead etc. Therefore, the tortoise will always be ahead of Achilles.
Invalid	
Some photographers are humans. Some doctors are not humans. Hence, no doctor is photographer.	

**Table 3:** Scenarios that may yield “conflicting” results about a signal’s complexity by HFD and AppEn.

EEG	Signal Characteristics	HFD	AppEn
1.	Non-fractal but irregular: Irregular and complex but lacking self-similarity	Low	High
2.	Fractal but predictable: Clear fractal properties, Predictable patterns	High	Low
3.	Sensitivity to different types of complexity: -strong spatial self-similarity -lacking temporal irregularity	High	Low

**Table 4:** Multivariate Tests of HFD estimations.

MANOVA Multivariate Tests of HIGUCHI Estimations				
Effect: Group, Pillais Trace				
Syllogism	Value	F	Sig. (*p*-Value)	Partial Eta Squared
Valid	0.573	2.395	0.028	0.573
invalid	0.320	0.840	0.624	0.320
Paradox	0.428	1.335	0.256	0.428

**Table 5:** Test between subjects.

HIGUCHI FD Analysis. MANOVA Results. Tests between Subjects Effects. F, *p*-Values, Partial Eta Squared (Using the Bonferroni Adjusted Alpha Value (*p* < 0.0035))			
CHANNEL	Valid	Invalid	Paradox
	F, *p*-Value, η^2^	F, *p*-Value, η^2^	F, *p*-Value, η^2^
AF3	6.082, 0.018, 0.138	1.547, 0.221, 0.039	0.964, 0.332, 0.025
F7	4.50, 0.040, 0.106	1.363, 0.250, 0.035	0.736, 0.396, 0.016
F3	3.303, 0.077, 0.080	0.242, 0.625, 0.006	0.299, 0.588, 0.008
FC5	5.422, 0.025, 0.125	0.238, 0.628, 0.006	0.135, 0.715, 0.004
T7	0.746, 0.393, 0.019	0.010, 0.922, 0.000	0.014, 0.906, 0.000
P7	2.840, 0.100, 0.070	0.156, 0.695, 0.004	0.376, 0.544, 0.010
O1	5.566, 0, 024, 0.128	0.710, 0.374, 0.021	0.186, 0.669, 0.005
O2	5.029, 0.031, 0.117	0.244, 0.624, 0.006	0.136, 0.712, 0.004
P8	3.136, 0.085, 0.076	0.172, 0.681, 0.004	0.148, 0.702, 0.004
T8	1.039, 0.315, 0.027	0.522, 0.474, 0.014	1.899, 0.176, 0.048
FC6	2.294, 0.138, 0.057	0.330, 0.596, 0.009	0.003, 0.957, 0.000
F4	8.227, 0.007, 0.178	0.199, 0.658, 0.005	0.156, 0.695, 0.004
F8	5.413, 0.025, 0.125	1.791, 0.189, 0.045	0.231, 0.634, 0.006
AF4	13.776, 0.001, 0.266	3.300, 0.077, 0.080	4.379, 0.043, 0.103

**Table 6:** Mean (SE) and One-way ANOVA of HFD, valid syllogism.

HFD, One-Way ANOVA, Syllogism: Valid				
Channel	Healthy Controls Mean (SE)	AN Mean (SE)	F-Stats	*p*-Value
Ch1	1.647 (0.021)	1.737 (0.026)	6.082	0.018 *
Ch2	1.658 (0.019)	1.730 (0.022)	4.541	0.040 *
Ch3	1.683 (0.022)	1.754 (0.028)	3.303	0.077
Ch4	1.690 (0.021)	1.774 (0.025)	5.422	0.025 *
Ch5	1.730 (0.025)	1.768 (0.027)	0.746	0.393
Ch6	1.701 (0.023)	1.769 (0.025)	2.840	0.100
Ch7	1.665 (0.022)	1.753 (0.024)	5.566	0.024 *
Ch8	1.655 (0.019)	1.733 (0.027)	5.029	0.031
Ch9	1.696 (0.018)	1.758 (0.030)	3.136	0.085
Ch10	1.719 (0.022)	1.759 (0.031)	1.039	0.315
Ch11	1.684 (0.022)	1.744 (0.030)	2.294	0.138
Ch12	1.675 (0.020)	1.773 (0.022)	8.227	0.007 *
Ch13	1.674 (0.023)	1.767 (0.028)	5.413	0.025 *
Ch14	1.625 (0.018)	1.746 (0.025)	13.77	0.001 **

Note: * *p* < 0.05, ** *p* < 0.01.

**Table 7:** Mean (SE) and One-way ANOVA of HFD, invalid syllogism.

HFD, One-Way ANOVA, Syllogism: Invalid				
Channel	Healthy Controls Mean (SE)	AN Mean (SE)	F-Stats	*p*-Value
Ch1	1.671 (0.017)	1.716 (0.036)	1.547	0.221
Ch2	1.676 (0.017)	1.715 (0.034)	1.363	0.250
Ch3	1.700 (0.017)	1.718 (0.034)	0.242	0.625
Ch4	1.706 (0.018)	1.725 (0.037)	0.238	0.628
Ch5	1.755 (0.020)	1.759 (0.035)	0.010	0.922
Ch6	1.732 (0.017)	1.745 (0.030)	0.156	0.695
Ch7	1.694 (0.019)	1.728 (0.033)	0.810	0.374
Ch8	1.697 (0.016)	1.714 (0.037)	0.244	0.624
Ch9	1.720 (0.019)	1.734 (0.041)	0.172	0.681
Ch10	1.747 (0.020)	1.717 (0.040)	0.522	0.474
Ch11	1.705 (0.018)	1.727 (0.037)	0.330	0.569
Ch12	1.698 (0.018)	1.713 (0.027)	0.199	0.658
Ch13	1.695 (0.017)	1.739 (0.029)	1.791	0.189
Ch14	1.648 (0.017)	1.709 (0.031)	3.300	0.077

**Table 8:** Mean (SE) and One-way ANOVA of HFD, paradox syllogism.

HFD, One-Way ANOVA, Syllogism: Paradox				
Channel	Healthy Controls Mean (SE)	AN Mean (SE)	F-Stats	*p*-Value
Ch1	1.678 (0.018)	1.713 (0.034)	0.964	0.332
Ch2	1.687 (0.018)	1.716 (0.026)	0.736	0.396
Ch3	1.713 (0.018)	1.732 (0.029)	0.299	0.588
Ch4	1.729 (0.016)	1.742 (0.034)	0.135	0.715
Ch5	1.763 (0.018)	1.759 (0.032)	0.014	0.906
Ch6	1.731 (0.017)	1.751 (0.025)	0.376	0.544
Ch7	1.705 (0.019)	1.720 (0.025)	0.186	0.669
Ch8	1.692 (0.017)	1.680 (0.030)	0.138	0.712
Ch9	1.733 (0.018)	1.719 (0.032)	0.148	0.702
Ch10	1.767 (0.018)	1.716 (0.036)	1.899	0.176
Ch11	1.718 (0.020)	1.716 (0.034)	0.003	0.957
Ch12	1.723 (0.017)	1.736 (0.033)	0.156	0.695
Ch13	1.719 (0.018)	1.736 (0.034)	0.231	0.634
Ch14	1.657 (0.017)	1.723 (0.025)	4.379	0.043 *

Note: * *p* < 0.05

**Table 9:** One-Way ANOVA of Grand Mean values (across all 14 channels) of HFD.

HFD, One-Way ANOVA, All Channels						
		Sum of Squares	df	Mean Square	F	Sig.
Mean valid Higuchi	Between groups Within groups Total	0.049 0.354 0.403	1 38 39	0.049 0.009	5.219	0.028
Mean invalid Higuchi	Between groups Within groups Total	0.004 0.318 0.323	1 38 39	0.004 0.008	0.510	0.479
Mean paradox Higuchi	Between groups Within groups Total	0.001 0.287 0.288	1 38 39	0.001 0.008	0.114	0.737

**Table 10:** Channels that exhibit significant (s) or not significant (ns) differences in AppEn estimates between the two groups.

Channel Index	Channel Name	Valid	Invalid	Paradox
1	AF3	ns	s	ns
2	F7	ns	s	ns
3	F3	s	s	s
4	FC5	ns	s	s
5	T7	s	s	s
6	P7	s	s	s
7	O1	s	s	s
8	O2	s	s	s
9	P8	s	s	s
10	T8	s	s	s
11	FC6	s	s	ns
12	F4	s	s	s
13	F8	s	s	s
14	AF4	ns	s	ns

**Table 11:** Channels at which there are significant differences in the *amplitude* values of EEG specific bands (rhythms) between the healthy controls and patients with AN.

	Alpha (α) (8–13 Hz)	Beta (β) (13–30 Hz)	Delta (δ) (1–4 Hz)	Theta (θ) (4–8 Hz)
Valid	-	AF3, F3 FC5, FC6	-	-
Invalid	P8	AF3, P7	-	F7, FC6, AF4
Paradox	F7, T8 FC6, AF4	O2	T7	-

**Table 12:** Channels of significant group effects (*p* < 0.05), of average of Grand Mean values of *Beta* relative power, for *valid* syllogism.

Channel	*X* ^2^	df	*p*
1 (AF3)	4.654	1	0.031
3 (F3)	4.183	1	0.041
4 (FC5)	4.776	1	0.029
7 (O1)	4.069	1	0.044
12 (F4)	5.278	1	0.022
14 (AF4)	5.941	1	0.015

**Table 13:** Channels of significant group effects of Grand Mean values of *Theta* relative power for (**a**) *valid* and (**b**) *invalid* cases.

(**a**)				
**Valid Case**	**Ch3 (F3)**	**Ch4 (FC5)**	**Ch12 (F4)**	**Ch14 (AF4)**
X^2^	4.899	3.957	5.941	7.693
df	1	1	1	1
*p*	0.027	0.047	0.015	0.006
(**b**)				
**Invalid Case**	**Ch1 (AF3)**	**Ch4 (FC5)**	**Ch11 (FC6)**	**Ch13 (F8)**
X^2^	4.268	3.680	5.031	4.770
df	1	1	1	1
*p*	0.039	0.055 (partial)	0.025	0.029

**Table 14:** (**a**): Summary of all EEG Rhythms Relative power on channels and “Brain’s Total Activation” (Average of Grand Means Relative Power, AGMRP) levels of analysis. Bold letters indicate significant group effects (i.e., patients with AN and Healthy Controls groups differ significantly). (**b**): Summary of all EEG Rhythms Average value of Grand Means Relative Power (AGMRP) (%) (Brain’s “Total Activity”). Bold letters indicate significant group effects.

(**a**)										
	**Valid**			**Invalid**			**Paradox**			
**EEG Wave RP**	**Channel** **Level** **Analysis**	**Brain’s** **Total** **Activation Analysis**		**Channel** **Level** **Analysis**	**Brain’s** **Total** **Activation Analysis**		**Channel** **Level** **Analysis**	**Brain’s** **Total** **Activation Analysis**		
alpha	No	No		No	No		No	No		
**beta**	**Yes** **AF3, F3** **FC5, O1** **F4, AF4**	Yes		No	No		No	No		
**gamma**	**Yes** **AF4**	No		No	No		No	No		
delta	No	No		No	No		No	No		
**theta**	**Yes** **F3, FC5** **F4, AF4**	**Yes**		**Yes** **AF3, FC5** **FC6, F8**	No		No	No		
(**b**)										
	Alpha		**Beta**		Delta		Gamma		**Theta**	
	CTRL	AN	CTRL	AN	CTRL	AN	CTRL	AN	CTRL	AN
Valid	18.013	17.864	**17.658**	**22.23**	31.96	27.90	10.54	13.73	**21.81**	**18.25**
Invalid	17.849	18.204	18.345	19.77	30.36	32.19	11.07	11.54	22.35	18.28
Paradox	17.65	19.11	19.11	18.54	30.09	32.00	12.91	11.08	20.20	19.33

**Table 15:** Optimum triad “feature–Syllogism–Classifier”, rendering the higher accuracy of the Higuchi FD and AppEn features of anorexia nervosa EEGs.

**(a) Without PCA**					
**Feature** **(Measure)**	**Syllogism**	**Best Classification** **Model**	**Accuracy, %**	**Area Under Curve (AUC)**	**OOPROC** **(Sensitivity, Specificity)**
Higuchi FD	Valid	SVM (Linear) SVM (Medium Gaussian) Ensemble (subspace Disc)	66.7 66.7 66.7	0.83 0.56 0.58	(0.33, 0.67) (0.33, 0.67) (0.50, 0.83)
AppEn	Invalid	Ensemble (Bagged Trees, BT)	83.3		
**(b) With PCA**					
**Feature** **(Measure)**	**Syllogism**	**Best Classification** **Model**	**Accuracy, %**	**Area Under Curve (AUC)**	**OOPROC** **(Sensitivity, Specificity)**
Higuchi FD	Valid	Linear Discriminant (*) Logistic Regression (*) KNN (Fine) (*)	75 75 75	0.78 0.76 0.75	(0.33, 0.83) (0.33, 0.83) (0.33, 0.83)
AppEn	Invalid	Ensemble (Bagged Trees, BT)	83.3		

Notes: * four (4) PCs were kept with variances: 86.7%, 4.6%, 3.3% and 2.7%.

**Table 16:** Optimum triad “feature–Syllogism–Classifier”, rendering the higher accuracy, using the amplitude of EEG Rhythms.

**(a) Without PCA**					
**Rhythm**	**Syllogism**	**Best Classification Model**	**Accuracy, %**	**Area Under Curve (AUC)**	**OOPROC** **(Sensitivity, Specificity)**
Alpha	Valid	SVM fine Gaus. Linear Discriminant	83.3 66.7	0.59 0.69	(0.00, 0.50) (0.38, 0.75)
Theta	Invalid	SVM Cubic KNN Fine KNN weighted	75 75 66.7	0.69 0.75 0.58	(0.50, 1.00) (0.50, 1.00) (0.67, 1.00)
**(b) With PCA**					
**Rhythm**	**Syllogism**	**Best Classification Model**	**Accuracy, %**	**Area Under Curve (AUC)**	**OOPROC** **(Sensitivity, Specificity)**
Alpha	Valid	SVM fine Gaus. KNN	83.3 66.7	0.50 0.50	(0.00, 0.50) (0.25, 0.50)
Theta	Invalid	KNN SVM Quadrat SVM cubic	75 66.7 66.7	0.75 0.78 0.83	(0.50, 1.00) (0.67, 1.00) (0.67, 1.00)

**Table 17:** Classification results of *Relative Power* of EEG rhythms (specific bands).

	Valid		Invalid		Paradox	
EEG Rhythm (Wave)	Model	Accuracy %	Model	Accuracy %	Model	Accuracy %
Alpha Relative Power	Fine Tree	75.6 no split 70.0 split	SVM Coarse Logistic regression	70 no split. 55 split	SVM Linear Ensemble BT	75.6 no split 85 split
Beta Relative Power	SVM Ensemble subspace Discr.	70.7 no split 85.0 split	SVM SVM (cubic)	75.0 no split 70.0 split	SVM fine Gaussian SVM cubic	68.3 no split 75.0 split
Delta Relative Power	Ensemble BT Logistic Regression	78.0 no split 60.0 split	SVM Fine Gaussian Ensemble subspace KNN	70.0 no split 90.0 split	SVM Quadrat. Ensemble BT	70.7 no split 75 split
Gamma Relative Power	KNN (cubic) SVM (linear)	73.2 no split 85.0 split	SVM Gaussian SVM Cubic	70.0 no split 65.0 split	Linear Discr. LD Logistic Regr.	73.3 no split 65.0 split
Theta Relative Power	SVM linear KNN weighted	68.3 no split 80.0 split	SVM fine Gaussian SVM Coarse Gaussian	70.0 no split 70.0 split	KNN weighted SVM Quadratic	70.7 no split 75.0 split

**Table 18:** Comparing AppEn and HFD results.

	Grand Mean AppEn			Grand Mean HFD		
Group	Valid	Invalid	Paradox	Valid	Invalid	Paradox
Healthy Controls	1.371	1.482	1.389	1.679	1.703	1.715
AN Patients	1.259	1.210	1.283	1.755	1.726	1.726
Comparison	AppEn_AN_ < AppEn_HC_	Same **	same	HFD_AN_ > HFD_HC_	same	same
Result *	Not normal	Not normal	Not normal	Normal	Normal	Normal

Note *: Normal = EEG Complexity in patients > EEG Complexity in healthy controls. **: same= as in the case of valid

## Data Availability

The data that supported the findings of the present work are available from the corresponding author upon reasonable request. The data are not publicly available due to privacy reasons.

## Supplementary Materials

The following supporting information can be downloaded at https://www.mdpi.com/article/10.3390/brainsci14030251/s1, Figure S1: Box plot of the distribution of the Grand Mean (across all channels) of the AppEn (per group), Figure S2: Box plot of the distribution of the Grand Mean (across all channels) of the AppEn (per gender), Figure S3: Box plot of the distribution of the Grand Mean (across all channels) of the Higuchi FD (per group), Figure S4: Box plot of the distribution of the Grand Mean (across all channels) of the Higuchi FD (per gender), Figure S5: Distribution of the % correctness, across syllogisms and groups, Figure S6: Distribution of the % degree of confidence (DoC), across syllogisms and groups. Table S1: Descriptive Statistics of Gran Mean (across all channels) of AppEn (per group), Table S2: Descriptive Statistics of Gran Mean (across all channels) of AppEn (per gender), Table S3: Normality test for Grand Mean (across all channels) of AppEn (per group), Table S4: Normality test for Grand Mean (across all channels) of AppEn (per gender), Table S5: Descriptive Statistics of Gran Mean (across all channels) of Higuchi FD (per group), Table S6: Descriptive Statistics of Gran Mean (across all channels) of the Higuchi FD (per gender), Table S7: Normality test for Grand Mean (across all channels) of Higuchi FD (per group), Table S8: Normality test for Grand Mean (across all channels) of Higuchi FD (per gender), Table S9: Correlation of Behavioral data (emotional intensity, emotional control, mood and number of correct answers) with AppEn, *Valid* syllogism, Healthy Controls, Table S10: Correlation of Behavioral data (emotional intensity, emotional control, mood and number of correct answers) with AppEn, *Invalid* syllogism, Healthy Controls, Table S11: Correlation of Behavioral data (emotional intensity, emotional control, mood and number of correct answers) with AppEn, *Paradox* syllogism, Healthy Controls, Table S12: Correlation of Behavioral data (emotional intensity, emotional control, mood, and number of correct answers) with AppEn, *valid* syllogism, AN patients, Table S13: Correlation of Behavioral data (emotional intensity, emotional control, mood, and number of correct answers) with AppEn, *Invalid* syllogism, AN patients, Table S14: Correlation of Behavioral data (emotional intensity, emotional control, mood, and number of correct answers) with AppEn, *Paradox* syllogism, AN patients, Table S15: Correlation of Behavioral data (emotional intensity, emotional control, mood, and number of correct answers) with HFD, *Valid* syllogism, Healthy Controls, Table S16: Correlation of Behavioral data (emotional intensity, emotional control, mood, and number of correct answers) with HFD, *Invalid* syllogism, Healthy Controls, Table S17: Correlation of Behavioral data (emotional intensity, emotional control, mood, and number of correct answers) with HFD, *Paradox* syllogism, Healthy Controls, Table S18: Correlation of Behavioral data (emotional intensity, emotional control, mood, and number of correct answers) with HFD, *Valid* syllogism, AN patients, Table S19: Correlation of Behavioral data (emotional intensity, emotional control, mood, and number of correct answers) with HFD, *Invalid* syllogism, AN patients, Table S20: Correlation of Behavioral data (emotional intensity, emotional control, mood, and number of correct answers) with HFD, *Paradox* syllogism, AN patients, Table S21: Correlations of Behavioral Data (% Degree of Confidence, DoC and % Correctness) with AppEn (Spearman’s rho, Nonparametric), Healthy Controls, Table S22: Correlations of Behavioral Data (% Degree of Confidence, DoC and % Correctness) with AppEn (Spearman’s rho, Nonparametric), AN patients, Table S23: Correlations of Behavioral Data (% Degree of Confidence, DoC and % Correctness) with HFD (Spearman’s rho, Nonparametric), Healthy Controls, Table S24: Correlations of Behavioral Data (% Degree of Confidence, DoC and % Correctness) with HFD (Spearman’s rho, Nonparametric), AN patients, Table S25: Independent samples *t*-test of % DoC and % Correctness, Table S26: Kruskal–Wallis test for the AppEn, valid syllogism, Table S27: Kruskal–Wallis test for AppEn, the invalid syllogism, Table S28: Kruskal–Wallis test for the AppEn, paradox syllogism, Table S29: Non-parametric, Kruskal–Wallis test for the *Grand mean values* of AppEn, Table S30: Kruskal–Wallis Test of EEG Alpha rhythm, valid syllogism, Table S31: Kruskal–Wallis Test of EEG Alpha rhythm, invalid syllogism, Table S32: Kruskal–Wallis Test of EEG Alpha rhythm, paradox syllogism, Table S33: Kruskal–Wallis Test of EEG Beta rhythm, valid syllogism, Table S34: Kruskal–Wallis Test of EEG Beta rhythm, invalid syllogism, Table S35: Kruskal–Wallis Test of EEG Beta rhythm, paradox syllogism, Table S36: Kruskal–Wallis Test of EEG Delta rhythm, valid syllogism, Table S37: Kruskal–Wallis Test of EEG Delta rhythm, invalid syllogism, Table S38: Kruskal–Wallis Test of EEG Delta rhythm, paradox syllogism, Table S39: Kruskal–Wallis Test of EEG Theta rhythm, valid syllogism, Table S40: Kruskal–Wallis Test of EEG Theta rhythm, invalid syllogism, Table S41: Kruskal–Wallis Test of EEG Theta rhythm, paradox syllogism.

## Appendix A

**Table A1:** (**a**) Extracted Approximate Entropy (AppEn) Descriptive features. (**b**) Extracted Higuchi Fractal Dimension (HFD) Descriptive features. (**c**) Extracted EEG Rhythms (alpha, beta, gamma, delta, theta) Descriptive features.

(**a**)					
**Extracted Approximate Entropy (AppEn) Descriptive Features** **(Number of Data Analyzed: 2(Groups) × 31(Subjects) × 14(Channels) = 868)**					**Target Feature**
	EEG Channels (electrodes)				Group
Participant	Ch-1	Ch-2	…	Ch-14	
CON-1	AppEn(1,1)	AppEn(1,2)	…	AppEn(1,14)	CON
CON-2	AppEn(2,1)	AppEn(2,2)	…	AppEn(2,14)	CON
…	…	…		…	
CON-30	AppEn(21,1)	AppEn(21,2)	…	AppEn(21,14)	CON
ED-1	AppEn(1,1)	AppEn(1,2)	…	AppEn(1,14)	ED
ED-2	AppEn(2,1)	AppEn(2,2)	…	AppEn(2,14)	ED
…	…	…	…	…	…
ED-31	AppEn(21,1)	AppEn(21,2)	…	AppEn(21,14)	ED
(**b**)					
**Extracted Higuchi Fractal Dimension (HFD) Descriptive Features** **(Number of data analyzed: 2(groups) × 31(subjects) × 14(channels) = 868)**					**Target Feature**
	EEG Channels (electrodes)				Group
Participant	Ch-1	Ch-2	…	Ch-14	
CON-1	HFD(1,1)	HFD(1,2)	…	HFD(1,14)	CON
CON-2	HFD(2,1)	HFD(2,2)	…	HFD(2,14)	CON
…	…	…		…	
CON-21	HFD(21,1)	HFD(21,2)	…	HFD(21,14)	CON
ED-1	HFD(1,1)	HFD(1,2)	…	HFD(1,14)	ED
ED-2	HFD(2,1)	HFD(2,2)	…	HFD(2,14)	ED
…	…	…		…	…
ED-21	HFD(21,1)	HFD(21,2)	…	HFD(21,14)	ED
(**c**)					
**Extracted EEG Rhythms (alpha, beta, gamma, delta, theta) Descriptive Features** **Number of data analyzed: 2(groups) × 31(subjects) × 4(Rhythms) × 14(channels) = 3472**					**Target Feature**
	EEG Channels (electrodes)				Group
Participant	Ch-1	Ch-2	…	Ch-14	
CON-1	α(1,1) β(1,1) δ(1,1) θ(1,1)	α(1,2) β(1,2) δ(1,2) θ(1,2)	…	α(1,14) β(1,14) δ(1,14) θ(1,14)	CON
CON-2	α(2,1) β(2,1) δ(2,1) θ(2,1)	α(2,2) β(2,2) δ(2,2) θ(2,2)	…	α(2,14) β(2,14) δ(2,14) θ(2,14)	CON
…	…	…		…	
CON-31	α(21,1) β(21,1) δ(21,1) θ(21,1)	α(21,2) β(21,2) δ(21,2) θ(21,2)	…	α(21,14) β(21,14) δ(21,14) θ(21,14)	CON
ED-1	α(1,1) β(1, 1) δ(1, 1) θ(1, 1)	α(1,2) β(1,2) δ(1,2) θ(1,2)	…	α(1,14) β(1,14) δ(1,14) θ(1,14)	ED
ED-2	α(2, 1) β(2, 1) δ(2, 1) θ(2, 1)	α(2,2) β(2,2) δ(2,2) θ(2,2)	…	α(2,14) β(2,14) δ(2,14) θ(2,14)	ED
…	…	…		…	…
ED-31	α(21,1) β(21,1) δ(21,1) θ(21,1)	α(21,2) β(21,2) δ(21,2) θ(21,2)	…	α(21,14) β(21,14) δ(21,14) θ(21,14)	ED

**Table A2:** EEG rhythms in brain regions and associated cognitive activities found in literature.

Brain Rhythm (Wave)	Frequency (HZ)	Amplitude (μV)	Brain Region	Associated Cognitive Activity
Delta (δ)	*f* <4	20–200	Frontal Temporal Occipital	Waking state Deep sleep Sleeping adults Normal in infants
Theta (θ)	4 ≤ *f* ≤ 7	20–100	Temporal Occipital	-Locomotion -Sensory information -Consciousness slips towards drowsiness -unconscious material -deep meditation -creative inspiration -emotional studies -drowsiness -spatial memory processes *Common in children and young adults than in older* *adults*
Alpha (α)	8 ≤ *f* ≤ 12	20–60	Occipital	-mental fatigue -cognitive disorders -no attention condition -during dreaming -awake but relaxed -attenuation as signal of visual activity -semantic memory processes to any type of task -during visually presented simulation
Beta (β)	13 ≤ *f* ≤ 29	2–30	Frontal Central Parietal	-active attention -active thinking -focus on external world -solving concrete problems -panic states -is found in normal adults -drowsiness -sensory motor area -light sleep -REM sleep
Gamma (γ)	30 ≤ *f* ≤ 64		-Anterior temporal -posterial temporal -occipital sites	-Prominent during higher focus and concentration -imbalanced excitatory/inhibitory Activity in autism group
