# Supplementary material for: Using Electroencephalogram-Extracted Nonlinear Complexity and Wavelet-Extracted Power Rhythm Features during the Performance of Demanding Cognitive Tasks (Aristotle’s Syllogisms) in Optimally Classifying Patients with Anorexia Nervosa
Source: Brain Sci. 2024 Mar 4;14(3):251. doi: 10.3390/brainsci14030251 (PMC10969099; doi:10.3390/brainsci14030251)
Supplement: Supplementary file 1 [file brainsci-14-00251-s001.zip › brainsci-2852987-supplementary-7.3.24.pdf]

SUPPLEMENTARY MATERIAL

Table S1: Descriptive Statistics of Grand Mean (across all channels) of AppEn (per group).

	Valid		Invalid		Paradox	
	Control	AN	Control	AN	Control	AN
Mean	1.371	1.259	1.482	1.210	1.389	1.283
St.Dev	0.401	0.130	0.207	0.201	0.335	0.099
Min	0.080	0.958	0.733	0.671	0.180	1.117
Max	1.727	1.408	1.742	1.415	1.674	1.403
Skewness	-1.891	-1.316	-2.125	-1.866	-2.237	-0.617
Kurtosis	3.159	1.513	5.830	4.541	5.651	-1.200

Table S2: Descriptive Statistics of Grand Mean (across all channels) of AppEn (per gender).

	Valid		Invalid		Paradox	
	Male	Female	Male	Female	Male	Female
Mean	1.342	1.337	1.413	1.383	1.353	1.357
St.Dev	0.346	0.349	0.087	0.249	0.372	0.280
Min	0.734	0.080	1.438	0.671	0.697	0.180
Max	1.567	1.727	1.650	1.742	1.586	1.674
Skewness	-2.037	-1.820	0.563	-1.136	-2.061	-2.256
Kurtosis	4.242	4.114	0.476	1.454	4.377	8.144

Table S3: Normality test for Grand Mean (across all channels) of AppEn (per group).

Tests of Normality

		Kolmogorov-Smirnov[a]			Shapiro-Wilk		
	group	Statistic	df	Sig.	Statistic	df	Sig.
mean_valid _AE	Control	0.293	28	0.000	0.743	28	0.000
	ED	0.231	12	0.078	0.884	12	0.099
mean_invalid _AE	Control	0.186	28	0.014	0.802	28	0.000
	ED	0.200	12	0.200*	0.826	12	0.019
mean_paradox_ AE	Control	0.244	28	0.000	0.738	28	0.000
	ED	0.237	12	0.062	0.873	12	0.070

*. This is a lower bound of the true significance.

a. Lilliefors Significance Correction

Table S4: Normality test for Grand Mean (across all channels) of AppEn (per gender).

Tests of Normality

	gender	Kolmogorov-Smirnov[a]			Shapiro-Wilk		
		Statistic	df	Sig.	Statistic	df	Sig.
mean_valid _AE	Male	0.355	5	0.038	0.721	5	0.016
	Female	0.187	35	0.003	0.834	35	0.000
mean_invalid _AE	Male	0.225	5	0.200*	0.895	5	0.381
	Female	0.118	35	0.200*	0.914	35	0.010
mean_paradox_ AE	Male	0.397	5	0.010	0.700	5	0.010
	Female	0.163	35	0.019	0.806	35	0.000

*. This is a lower bound of the true significance.

a. Lilliefors Significance Correction

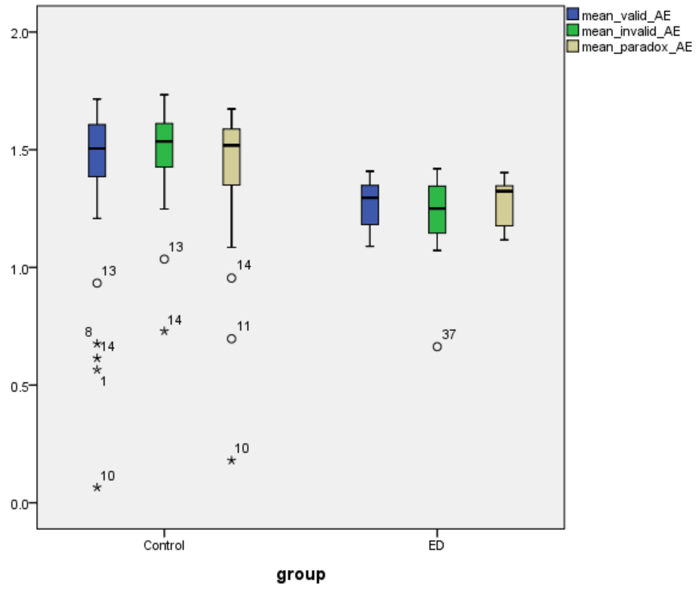

Figure S1: Box plot of the distribution of the Grand Mean (across all channels) of the AppEn (per group).

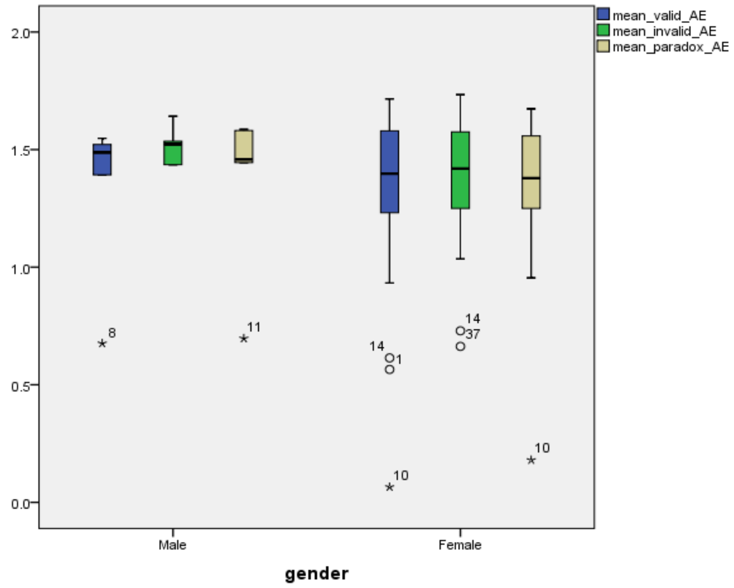

Figure S2: Box plot of the distribution of the Grand Mean (across all channels) of the AppEn (per gender).

Table S5: Descriptive Statistics of Grand Mean (across all channels) of Higuchi FD, per group.

	Valid		Invalid		Paradox	
	Control	AN	Control	AN	Control	AN
Mean	1.679	1.755	1.703	1.726	1.715	1.726
St.Dev	0.102	0.079	0.084	0.105	0.084	0.092
Min	1.488	1.639	1.544	1.543	1.574	1.594
Max	1.887	1.905	1.968	1.910	1.967	1.888
Skewness	-0.299	0.393	0.789	0.337	0.722	0.347
Kurtosis	-0.519	0.637	2.294	0.123	1.620	-0.829

Table S6: Descriptive Statistics of Grand Mean (across all channels) of Higuchi FD, per gender.

	Valid		Invalid		Paradox	
	Male	Female	Male	Female	Male	Female
Mean	1.641	1.710	1.684	1.714	1.677	1.724
St.Dev	0.079	0.102	0.065	0.094	0.050	0.088
Min	1.546	1.488	1.618	1.543	1.606	1.574
Max	1.723	1.905	1.752	1.968	1.728	1.967
Skewness	-0.356	-0.418	0.293	0.579	-0.741	0.480
Kurtosis	-2.697	-0.003	-3.050	0.940	-1.265	0.326

Table S7: Normality test for Grand Mean (across all channels) of Higuchi FD (per group).

Tests of Normality

	group	Kolmogorov-Smirnov[a]			Shapiro-Wilk		
		Statistic	df	Sig.	Statistic	df	Sig.
mean_valid _higuchi	Control	0.116	28	0.200*	0.971	28	0.601
	ED	0.116	12	0.200*	0.974	12	0.951
mean_invalid _higuchi	Control	0.129	28	0.200*	0.946	28	0.158
	ED	0.188	12	0.200*	0.945	12	0.562
mean_paradox_higuchi	Control	0.085	28	0.200*	0.957	28	0.288
	ED	0.134	12	0.200*	0.956	12	0.732

*. This is a lower bound of the true significance.

a. Lilliefors Significance Correction

Table S8: Normality test for Grand Mean (across all channels) of Higuchi FD (per gender).

Tests of Normality

	gender	Kolmogorov-Smirnov[a]			Shapiro-Wilk		
		Statistic	df	Sig.	Statistic	df	Sig.
mean_valid _higuchi	Male	0.214	5	0.200*	0.893	5	0.373
	Female	0.080	35	0.200*	0.974	35	0.563
mean_invalid _higuchi	Male	0.249	5	0.200*	0.829	5	0.138
	Female	0.122	35	0.200*	0.959	35	0.210
mean_paradox_ higuchi	Male	0.267	5	0.200*	0.915	5	0.497
	Female	0.075	35	0.200*	0.975	35	0.598

*. This is a lower bound of the true significance.

a. Lilliefors Significance Correction

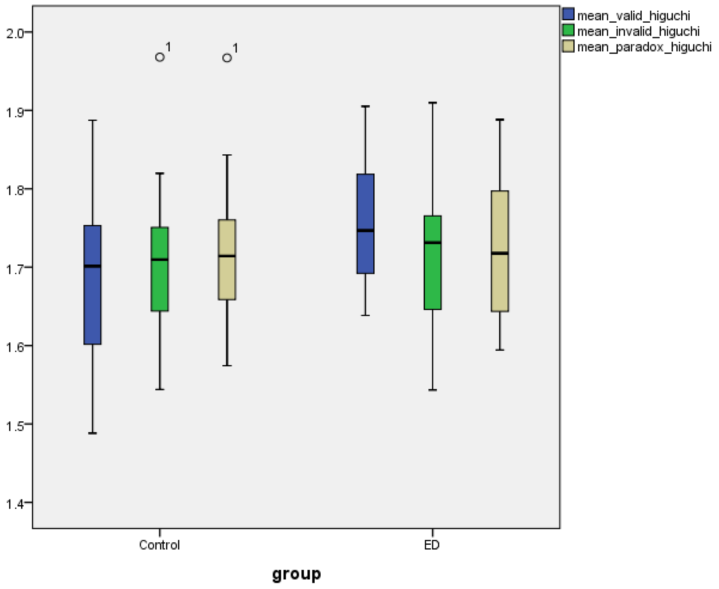

Figure S3: Box plot of the distribution of the Grand Mean (across all channels) of the Higuchi FD (per group).

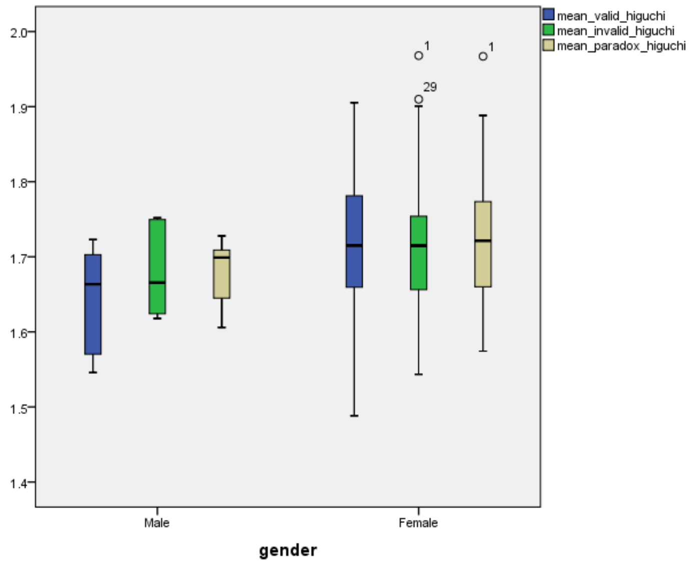

Figure S4: Box plot of the distribution of the Grand Mean (across all channels) of the Higuchi FD (per gender).

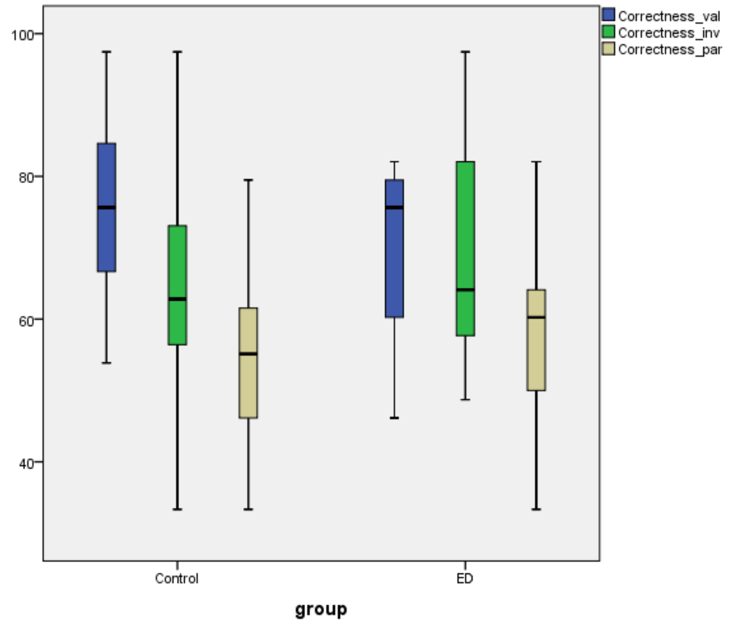

Figure S5: Distribution of the % correctness, across syllogisms and groups.

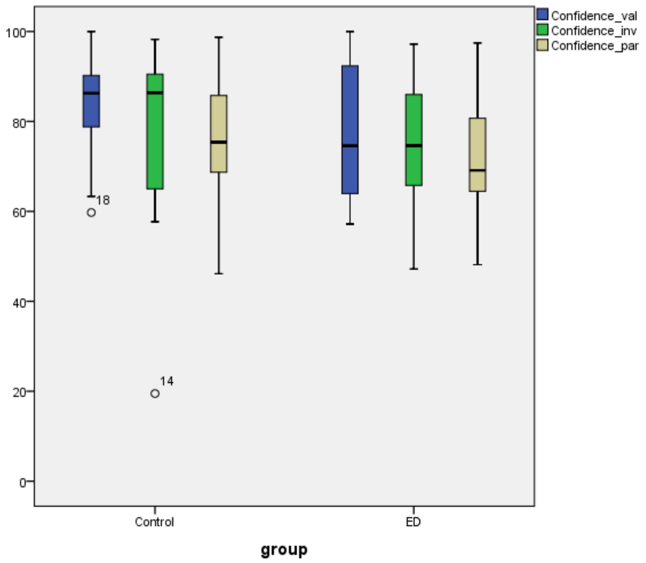

Figure S6: Distribution of the % degree of confidence (DoC), across syllogisms and groups.

Table S9: Correlation of Behavioral data (emotional intensity, emotional control, mood and number of correct answers) with **AppEn**, ***Valid*** syllogism, Healthy Controls.

Correlations

		valid_emotional_intensity_AE	valid_emotional_control_AE	valid_mood_AE	valid_correct_answers_AE	mean_valid_AE
valid_emotional_intensity_AE	Pearson Correlation	1	-0.369	-0.323	-0.064	-0.205
	Sig. (2-tailed)		0.053	0.094	0.746	0.294
	N	28	28	28	28	28
valid_emotional_control_AE	Pearson Correlation	-0.369	1	0.352	0.041	0.096
	Sig. (2-tailed)	0.053		0.067	0.837	0.626
	N	28	28	28	28	28
valid_mood_AE	Pearson Correlation	-0.323	0.352	1	-0.115	0.141
	Sig. (2-tailed)	0.094	0.067		0.561	0.476
	N	28	28	28	28	28
valid_correct_answers_AE	Pearson Correlation	-0.064	0.041	-0.115	1	-0.036
	Sig. (2-tailed)	0.746	0.837	0.561		0.856
	N	28	28	28	28	28
mean_valid_AE	Pearson Correlation	-0.205	0.096	0.141	-0.036	1
	Sig. (2-tailed)	0.294	0.626	0.476	0.856	
	N	28	28	28	28	28

Table S10: Correlation of Behavioral data (emotional intensity, emotional control, mood and number of correct answers) with **AppEn**, ***Invalid*** syllogism, Healthy Controls.

Correlations

		invalid_emotional_ intensity_AE	invalid_emotional_ control_AE	invalid_mood _AE	invalid_correct_ answers_AE	mean_invalid _AE
invalid_emotional_ intensity_AE	Pearson Correlation	1	-0.627**	-0.539**	-0.452*	-0.103
	Sig. (2-tailed)		0.000	0.003	0.016	0.602
	N	28	28	28	28	28
invalid_emotional_ control_AE	Pearson Correlation	-0.627**	1	0.480**	0.480**	-0.203
	Sig. (2-tailed)	0.000		0.010	0.010	0.300
	N	28	28	28	28	28
invalid_mood _AE	Pearson Correlation	-0.539**	0.480**	1	0.440*	0.089
	Sig. (2-tailed)	0.003	0.010		0.019	0.654
	N	28	28	28	28	28
invalid_correct_ answers_AE	Pearson Correlation	-0.452*	0.480**	0.440*	1	-0.147
	Sig. (2-tailed)	0.016	0.010	0.019		0.454
	N	28	28	28	28	28
mean_invalid _AE	Pearson Correlation	-0.103	-0.203	0.089	-0.147	1
	Sig. (2-tailed)	0.602	0.300	0.654	0.454	
	N	28	28	28	28	28

**. Correlation is significant at the 0.01 level (2-tailed).

*. Correlation is significant at the 0.05 level (2-tailed).

Table S11: Correlation of Behavioral data (emotional intensity, emotional control, mood and number of correct answers) with **AppEn**, ***Paradox*** syllogism, Healthy Controls.

Correlations

		paradox_emotional_ intensity_AE	paradox_emotional_ control_AE	paradox_mood _AE	paradox_correct _answers_AE	mean_paradox _AE
paradox_emotional_ intensity_AE	Pearson Correlation	1	-0.630**	-0.325	0.028	-0.272
	Sig. (2-tailed)		0.000	0.092	0.887	0.162
	N	28	28	28	28	28
paradox_emotional_ control_AE	Pearson Correlation	-0.630**	1	0.311	-0.194	0.421*
	Sig. (2-tailed)	0.000		0.107	0.323	0.026
	N	28	28	28	28	28
paradox_mood _AE	Pearson Correlation	-0.325	0.311	1	-0.244	0.164
	Sig. (2-tailed)	0.092	0.107		0.211	0.405
	N	28	28	28	28	28
paradox_correct_ answers_AE	Pearson Correlation	0.028	-0.194	-0.244	1	-0.067
	Sig. (2-tailed)	0.887	0.323	0.211		0.734
	N	28	28	28	28	28
mean_paradox _AE	Pearson Correlation	-0.272	0.421*	0.164	-0.067	1
	Sig. (2-tailed)	0.162	0.026	0.405	0.734	
	N	28	28	28	28	28

**. Correlation is significant at the 0.01 level (2-tailed).

*. Correlation is significant at the 0.05 level (2-tailed).

Table S12: Correlation of Behavioral data (emotional intensity, emotional control, mood, and number of correct answers) with **AppEn**, ***valid*** syllogism, AN patients.

Correlations

		valid_emotional_ intensity_AE	valid_emotional_ control_AE	valid_mood _AE	valid_correct_ answers_AE	mean_valid _AE
valid_emotional_ intensity_AE	Pearson Correlation	1	-0.543	-0.535	0.282	0.217
	Sig. (2-tailed)		0.068	0.073	0.375	0.497
	N	12	12	12	12	12
valid_emotional_ control_AE	Pearson Correlation	-0.543	1	0.493	-0.189	-0.048
	Sig. (2-tailed)	0.068		0.103	0.556	0.883
	N	12	12	12	12	12

valid_mood _AE	Pearson Correlation	-0.535	0.493	1	-0.328	-0.212
	Sig. (2-tailed)	0.073	0.103		0.298	0.508
	N	12	12	12	12	12
valid_correct_ answers_AE	Pearson Correlation	0.282	-0.189	-0.328	1	0.089
	Sig. (2-tailed)	0.375	0.556	0.298		0.784
	N	12	12	12	12	12
mean_valid _AE	Pearson Correlation	0.217	-0.048	-0.212	0.089	1
	Sig. (2-tailed)	0.497	0.883	0.508	0.784	
	N	12	12	12	12	12

Table S13: Correlation of Behavioral data (emotional intensity, emotional control, mood, and number of correct answers) with **AppEn**, ***Invalid*** syllogism, AN patients.

Correlations

		invalid_emotional_ intensity_AE	invalid_emotional_ control_AE	invalid_mood _AE	invalid_correct_ answers_AE	mean_invalid _AE
invalid_emotional_ intensity_AE	Pearson Correlation	1	-0.429	-0.523	-0.466	-0.148
	Sig. (2-tailed)		0.164	0.081	0.126	0.646
	N	12	12	12	12	12
invalid_emotional_ control_AE	Pearson Correlation	-0.429	1	0.672*	0.100	0.635*
	Sig. (2-tailed)	0.164		0.017	0.756	0.027
	N	12	12	12	12	12
invalid_mood _AE	Pearson Correlation	-0.523	0.672*	1	0.049	0.480
	Sig. (2-tailed)	0.081	0.017		0.879	0.115
	N	12	12	12	12	12
invalid_correct_ answers_AE	Pearson Correlation	-0.466	0.100	0.049	1	-0.167
	Sig. (2-tailed)	0.126	0.756	0.879		0.603
	N	12	12	12	12	12
mean_invalid _AE	Pearson Correlation	-0.148	0.635*	0.480	-0.167	1
	Sig. (2-tailed)	0.646	0.027	0.115	0.603	
	N	12	12	12	12	12

*. Correlation is significant at the 0.05 level (2-tailed).

Table S14: Correlation of Behavioral data (emotional intensity, emotional control, mood, and number of correct answers) with **AppEn, *Paradox*** syllogism, AN patients.

Correlations

		paradox_emotional_ intensity_AE	paradox_emotional_ control_AE	paradox_mood _AE	paradox_correct_ answers_AE	mean_paradox _AE
paradox_emotional_ intensity_AE	Pearson Correlation	1	-0.706*	-0.243	0.413	0.227
	Sig. (2-tailed)		0.010	0.447	0.182	0.477
	N	12	12	12	12	12
paradox_emotional_ control_AE	Pearson Correlation	-0.706*	1	0.587*	0.082	-0.530
	Sig. (2-tailed)	0.010		0.045	0.799	0.076
	N	12	12	12	12	12
paradox_mood _AE	Pearson Correlation	-0.243	0.587*	1	0.355	-0.472
	Sig. (2-tailed)	0.447	0.045		0.258	0.121
	N	12	12	12	12	12
paradox_correct_ answers_AE	Pearson Correlation	0.413	0.082	0.355	1	-0.300
	Sig. (2-tailed)	0.182	0.799	0.258		0.344
	N	12	12	12	12	12
mean_paradox _AE	Pearson Correlation	0.227	-0.530	-0.472	-0.300	1
	Sig. (2-tailed)	0.477	0.076	0.121	0.344	
	N	12	12	12	12	12

*. Correlation is significant at the 0.05 level (2-tailed).

Table S15: Correlation of Behavioral data (emotional intensity, emotional control, mood, and number of correct answers) with **HFD,** ***Valid*** syllogism, Healthy Controls.

Correlations

		valid_emotional_ intensity_HI	valid_emotional_ control_HI	valid_mood _HI	valid_correct_ answers_HI	mean_valid _HI
valid_emotional_ intensity_HI	Pearson Correlation	1	-0.369	-0.323	-0.064	0.116
	Sig. (2-tailed)		0.053	0.094	0.746	0.558
	N	28	28	28	28	28
valid_emotional_ control_HI	Pearson Correlation	-0.369	1	0.352	0.041	0.134
	Sig. (2-tailed)	0.053		0.067	0.837	0.496
	N	28	28	28	28	28
valid_mood _HI	Pearson Correlation	-0.323	0.352	1	-0.115	0.259
	Sig. (2-tailed)	0.094	0.067		0.561	0.183
	N	28	28	28	28	28
valid_correct_ answers_HI	Pearson Correlation	-0.064	0.041	-0.115	1	-0.049
	Sig. (2-tailed)	0.746	0.837	0.561		0.806
	N	28	28	28	28	28
mean_valid _HI	Pearson Correlation	0.116	0.134	0.259	-0.049	1
	Sig. (2-tailed)	0.558	0.496	0.183	0.806	
	N	28	28	28	28	28

Table S16: Correlation of Behavioral data (emotional intensity, emotional control, mood, and number of correct answers) with **HFD,** ***Invalid*** syllogism, Healthy Controls.

Correlations

		invalid_emotional_ intensity_HI	invalid_emotional_ control_HI	invalid_mood _HI	invalid_correct_ answers_HI	mean_invalid _HI
invalid_emotional_ intensity_HI	Pearson Correlation	1	-0.627**	-0.539**	-0.452*	-0.322
	Sig. (2-tailed)		0.000	0.003	0.016	0.095
	N	28	28	28	28	28
invalid_emotional_ control_HI	Pearson Correlation	-0.627**	1	0.480**	0.480**	0.107
	Sig. (2-tailed)	0.000		0.010	0.010	0.587
	N	28	28	28	28	28
invalid_mood _HI	Pearson Correlation	-0.539**	0.480**	1	0.440*	0.010
	Sig. (2-tailed)	0.003	0.010		0.019	0.958
	N	28	28	28	28	28
invalid_correct_ answers_HI	Pearson Correlation	-0.452*	0.480**	0.440*	1	-0.044
	Sig. (2-tailed)	0.016	0.010	0.019		0.823
	N	28	28	28	28	28
mean_invalid _HI	Pearson Correlation	-0.322	0.107	0.010	-0.044	1
	Sig. (2-tailed)	0.095	0.587	0.958	0.823	
	N	28	28	28	28	28

**. Correlation is significant at the 0.01 level (2-tailed).

*. Correlation is significant at the 0.05 level (2-tailed).

Table S17: Correlation of Behavioral data (emotional intensity, emotional control, mood, and number of correct answers) with **HFD,** ***Paradox*** syllogism, Healthy Controls.

Correlations

		paradox_emotional_intensity_HI	paradox_emotional_control_HI	paradox_mood_HI	paradox_correct_answers_HI	mean_paradox_HI
paradox_emotional_intensity_HI	Pearson Correlation	1	-0.630**	-0.325	0.028	-0.033
	Sig. (2-tailed)		0.000	0.092	0.887	0.868
	N	28	28	28	28	28
paradox_emotional_control_HI	Pearson Correlation	-0.630**	1	0.311	-0.194	0.053
	Sig. (2-tailed)	0.000		0.107	0.323	0.788
	N	28	28	28	28	28
paradox_mood_HI	Pearson Correlation	-0.325	0.311	1	-0.244	-0.076
	Sig. (2-tailed)	0.092	0.107		0.211	0.702
	N	28	28	28	28	28
paradox_correct_answers_HI	Pearson Correlation	0.028	-0.194	-0.244	1	-0.023
	Sig. (2-tailed)	0.887	0.323	0.211		0.907
	N	28	28	28	28	28
mean_paradox_HI	Pearson Correlation	-0.033	0.053	-0.076	-0.023	1
	Sig. (2-tailed)	0.868	0.788	0.702	0.907	
	N	28	28	28	28	28

**. Correlation is significant at the 0.01 level (2-tailed).

Table S18: Correlation of Behavioral data (emotional intensity, emotional control, mood, and number of correct answers) with **HFD,** ***Valid*** syllogism, AN patients.

Correlations

		valid_emotional_intensity_HI	valid_emotional_control_HI	valid_mood_HI	valid_correct_answers_HI	mean_valid_HI
valid_emotional_intensity_HI	Pearson Correlation	1	-0.543	-0.535	0.282	-0.146
	Sig. (2-tailed)		0.068	0.073	0.375	0.650
	N	12	12	12	12	12
valid_emotional_control_HI	Pearson Correlation	-0.543	1	0.493	-0.189	-0.315
	Sig. (2-tailed)	0.068		0.103	0.556	0.318
	N	12	12	12	12	12
valid_mood_HI	Pearson Correlation	-0.535	0.493	1	-0.328	0.092
	Sig. (2-tailed)	0.073	0.103		0.298	0.776
	N	12	12	12	12	12
valid_correct_answers_HI	Pearson Correlation	0.282	-0.189	-0.328	1	0.346
	Sig. (2-tailed)	0.375	0.556	0.298		0.270
	N	12	12	12	12	12
mean_valid_HI	Pearson Correlation	-0.146	-0.315	0.092	0.346	1
	Sig. (2-tailed)	0.650	0.318	0.776	0.270	
	N	12	12	12	12	12

Table S19: Correlation of Behavioral data (emotional intensity, emotional control, mood, and number of correct answers) with **HFD,** ***Invalid*** syllogism, AN patients.

Correlations

		invalid_emotional_intensity_HI	invalid_emotional_control_HI	invalid_mood_HI	invalid_correct_answers_HI	mean_invalid_HI
invalid_emotional_intensity_HI	Pearson Correlation	1	-0.429	-0.523	-0.466	-0.588*
	Sig. (2-tailed)		0.164	0.081	0.126	0.045
	N	12	12	12	12	12
invalid_emotional_control_HI	Pearson Correlation	-0.429	1	0.672*	0.100	0.364
	Sig. (2-tailed)	0.164		0.017	0.756	0.244
	N	12	12	12	12	12
invalid_mood_HI	Pearson Correlation	-0.523	0.672*	1	0.049	0.378
	Sig. (2-tailed)	0.081	0.017		0.879	0.226
	N	12	12	12	12	12

invalid_correct_<br>answers_HI	Pearson Correlation<br>Sig. (2-tailed)<br>N	-0.466<br>0.126<br>12	0.100<br>0.756<br>12	0.049<br>0.879<br>12	1<br><br>12	0.009<br>0.977<br>12
mean_invalid<br>_HI	Pearson Correlation<br>Sig. (2-tailed)<br>N	-0.588*<br>0.045<br>12	0.364<br>0.244<br>12	0.378<br>0.226<br>12	0.009<br>0.977<br>12	1<br><br>12

*. Correlation is significant at the 0.05 level (2-tailed).

Table S20: Correlation of Behavioral data (emotional intensity, emotional control, mood, and number of correct answers) with **HFD, *Paradox*** syllogism, AN patients.

Correlations

		paradox_emotional_<br>intensity_HI	paradox_emotional_<br>control_HI	paradox_mood<br>_HI	paradox_correct_<br>answers_HI	mean_paradox<br>_HI
paradox_emotional_<br>intensity_HI	Pearson Correlation<br>Sig. (2-tailed)<br>N	1<br><br>12	-0.706*<br>0.010<br>12	-0.243<br>0.447<br>12	0.413<br>0.182<br>12	0.359<br>0.252<br>12
paradox_emotional_<br>control_HI	Pearson Correlation<br>Sig. (2-tailed)<br>N	-0.706*<br>0.010<br>12	1<br><br>12	0.587*<br>0.045<br>12	0.082<br>0.799<br>12	-0.574<br>0.051<br>12
paradox_mood<br>_HI	Pearson Correlation<br>Sig. (2-tailed)<br>N	-0.243<br>0.447<br>12	0.587*<br>0.045<br>12	1<br><br>12	0.355<br>0.258<br>12	-0.244<br>0.444<br>12
paradox_correct_<br>answers_HI	Pearson Correlation<br>Sig. (2-tailed)<br>N	0.413<br>0.182<br>12	0.082<br>0.799<br>12	0.355<br>0.258<br>12	1<br><br>12	0.284<br>0.371<br>12
mean_paradox<br>_HI	Pearson Correlation<br>Sig. (2-tailed)<br>N	0.359<br>0.252<br>12	-0.574<br>0.051<br>12	-0.244<br>0.444<br>12	0.284<br>0.371<br>12	1<br><br>12

*. Correlation is significant at the 0.05 level (2-tailed).

Table S21: Correlations of Behavioral Data (% Degree of Confidence, DoC and % Correctness) with AppEn (Spearman's rho, Nonparametric), Healthy Controls.

Correlations

			mean_valid_<br>AE	mean_invalid_<br>AE	mean_<br>paradox_<br>AE	Confidence_val	Confidence_inv	Confidence_par	Correctness_val	Correctness_inv	Correctness_par
Spearhman's rho	mean_valid_<br>AE	Correlation Coefficient<br>Sig. (2-tailed)<br>N	1.000<br>.<br>28	0.711**<br>0.000<br>28	0.568**<br>0.002<br>28	0.180<br>0.360<br>28	0.018<br>0.929<br>28	0.169<br>0.390<br>28	0.010<br>0.961<br>28	0.294<br>0.129<br>28	-0.111<br>0.573<br>28
	mean_invali<br>d_AE	Correlation Coefficient<br>Sig. (2-tailed)<br>N	0.711**<br>0.000<br>28	1.000<br>.<br>28	0.530**<br>0.004<br>28	0.110<br>0.576<br>28	0.092<br>0.643<br>28	0.095<br>0.632<br>28	0.061<br>0.758<br>28	0.196<br>0.318<br>28	-0.078<br>0.694<br>28
	mean_parad<br>ox_ AE	Correlation Coefficient<br>Sig. (2-tailed)<br>N	0.568**<br>0.002<br>28	0.530**<br>0.004<br>28	1.000<br>.<br>28	0.157<br>0.426<br>28	0.252<br>0.196<br>28	0.299<br>0.122<br>28	0.180<br>0.359<br>28	0.013<br>0.947<br>28	0.001<br>0.994<br>28
	Confidence_<br>val	Correlation Coefficient<br>Sig. (2-tailed)<br>N	0.180<br>0.360<br>28	0.110<br>0.576<br>28	0.157<br>0.426<br>28	1.000<br>.<br>28	0.693**<br>0.000<br>28	0.848**<br>0.000<br>28	0.284<br>0.143<br>28	0.052<br>0.791<br>28	-0.099<br>0.615<br>28
	Confidence_<br>inv	Correlation Coefficient<br>Sig. (2-tailed)<br>N	0.018<br>0.929<br>28	0.092<br>0.643<br>28	0.252<br>0.196<br>28	0.693**<br>0.000<br>28	1.000<br>.<br>28	0.779**<br>0.000<br>28	0.012<br>0.950<br>28	0.290<br>0.135<br>28	-0.111<br>0.575<br>28
	Confidence_<br>par	Correlation Coefficient<br>Sig. (2-tailed)<br>N	0.169<br>0.390<br>28	0.095<br>0.632<br>28	0.299<br>0.122<br>28	0.848**<br>0.000<br>28	0.779**<br>0.000<br>28	1.000<br>.<br>28	0.137<br>0.487<br>28	0.066<br>0.738<br>28	-0.167<br>0.397<br>28

	Correctness_val	Correlation Coefficient	0.010	0.061	0.180	0.284	0.012	0.137	1.000	-0.555**	0.306
		Sig. (2-tailed)	0.961	0.758	0.359	0.143	0.950	0.487	.	0.002	0.114
		N	28	28	28	28	28	28	28	28	28
	Correctness_inv	Correlation Coefficient	0.294	0.196	0.013	0.052	0.290	0.066	-0.555**	1.000	-0.435*
		Sig. (2-tailed)	0.129	0.318	0.947	0.791	0.135	0.738	0.002	.	0.021
		N	28	28	28	28	28	28	28	28	28
	Correctness_par	Correlation Coefficient	-0.111	-0.078	0.001	-0.099	-0.111	-0.167	0.306	-0.435*	1.000
		Sig. (2-tailed)	0.573	0.694	0.994	0.615	0.575	0.397	0.114	0.021	.
		N	28	28	28	28	28	28	28	28	28

**. Correlation is significant at the 0.01 level (2-tailed).

*. Correlation is significant at the 0.05 level (2-tailed).

Table S22: Correlations of Behavioral Data (% Degree of Confidence, DoC and % Correctness) with AppEn (Spearman's rho, Nonparametric), AN patients.

Correlations

			mean_valid_AE	mean_invalid_AE	mean_paradox_AE	Confidence_val	Confidence_inv	Confidence_par	Correctness_val	Correctness_inv	Correctness_par
Spearhman's rho	mean_valid_AE	Correlation Coefficient	1.000	0.280	0.524	0.427	0.720**	0.401	0.226	-0.246	-0.070
		Sig. (2-tailed)	.	0.379	0.080	0.127	0.008	0.196	0.480	0.440	0.828
		N	12	12	12	12	12	12	12	12	12
	mean_invalid_AE	Correlation Coefficient	0.280	1.000	0.350	0.238	0.503	0.190	-0.053	0.123	-0.112
		Sig. (2-tailed)	0.379	.	0.265	0.457	0.095	0.554	0.870	0.703	0.728
		N	12	12	12	12	12	12	12	12	12
	mean_paradox_ AE	Correlation Coefficient	0.524	0.350	1.000	0.336	0.336	0.289	0.155	-0.479	-0.446
		Sig. (2-tailed)	0.080	0.265	.	0.286	0.286	0.363	0.629	0.115	0.147
		N	12	12	12	12	12	12	12	12	12
	Confidence_val	Correlation Coefficient	0.427	0.238	0.336	1.000	0.797**	0.986**	0.629*	0.127	0.109
		Sig. (2-tailed)	0.167	0.457	0.286	.	0.002	0.000	0.028	0.695	0.736
		N	12	12	12	12	12	12	12	12	12
	Confidence_inv	Correlation Coefficient	0.720**	0.503	0.336	0.797 **	1.000	0.782**	0.459	0.222	0.077
		Sig. (2-tailed)	0.008	0.095	0.286	0.002	.	0.003	0.133	0.488	0.812
		N	12	12	12	12	12	12	12	12	12
	Confidence_par	Correlation Coefficient	0.401	0.190	0.289	0.986 **	0.782**	1.000	0.680*	0.096	0.205
		Sig. (2-tailed)	0.196	0.554	0.363	0.000	0.003	.	0.015	0.767	0.523
		N	12	12	12	12	12	12	12	12	12
	Correctness_val	Correlation Coefficient	0.226	-0.053	0.155	0.629*	0.459	0.680*	1.000	-0.203	0.473
		Sig. (2-tailed)	0.480	0.870	0.629	0.028	0.133	0.015	.	0.527	0.120
		N	12	12	12	12	12	12	12	12	12
	Correctness_inv	Correlation Coefficient	-0.246	0.123	-0.479	0.127	0.222	0.096	-0.203	1.000	0.035
		Sig. (2-tailed)	0.440	0.703	0.115	0.695	0.488	0.767	0.527	.	0.913
		N	12	12	12	12	12	12	12	12	12
	Correctness_par	Correlation Coefficient	-0.070	-0.112	-0.446	0.109	0.077	0.205	0.473	0.035	1.000
		Sig. (2-tailed)	0.828	0.728	0.147	0.736	0.812	0.523	0.120	0.913	.
		N	12	12	12	12	12	12	12	12	12

**. Correlation is significant at the 0.01 level (2-tailed).

*. Correlation is significant at the 0.05 level (2-tailed).

Table S23: Correlations of Behavioral Data (% Degree of Confidence, DoC and % Correctness) with HFD (Spearman's rho, Nonparametric), Healthy Controls.

Correlations

			mean_valid_higuchi	mean_invalid_higuchi	mean_paradox_higuchi	Confidence_val	Confidence_inv	Confidence_par	Correctness_val	Correctness_inv	Correctness_par
Spearhman's rho	mean_valid_higuchi	Correlation Coefficient	1.000	0.456 *	0.528**	-0.022	-0.135	-0.025	0.136	0.007	0.035
		Sig. (2-tailed)	.	0.015	0.004	0.912	0.493	0.901	0.490	0.973	0.861
		N	28	28	28	28	28	28	28	28	28
	mean_invalid_higuchi	Correlation Coefficient	0.456*	1.000	0.719 **	0.255	0.254	0.264	0.005	0.195	-0.104
		Sig. (2-tailed)	0.015	.	0.000	0.190	0.192	0.175	0.981	0.321	0.597
		N	28	28	28	28	28	28	28	28	28
	mean_paradox_higuchi	Correlation Coefficient	0.528**	0.719 **	1.000	0.162	0.246	0.240	-0.036	0.167	-0.069
		Sig. (2-tailed)	0.004	0.000	.	0.409	0.207	0.219	0.855	0.395	0.729

		Sig. (2-tailed)<br>N	28	28	28	28	28	28	28	28	28
	Confidence_val	Correlation<br>Coefficient<br>Sig. (2-tailed)<br>N	-0.022<br>0.912<br>28	0.255<br>0.190<br>28	0.162<br>0.409<br>28	1.000<br>.<br>28	0.693**<br>0.000<br>28	0.848**<br>0.000<br>28	0.284<br>0.143<br>28	0.052<br>0.791<br>28	-0.099<br>0.615<br>28
	Confidence_inv	Correlation<br>Coefficient<br>Sig. (2-tailed)<br>N	-0.135<br>0.493<br>28	0.254<br>0.192<br>28	0.246<br>0.207<br>28	0.693**<br>0.000<br>28	1.000<br>.<br>28	0.779**<br>0.000<br>28	0.012<br>0.950<br>28	0.290<br>0.135<br>28	-0.111<br>0.575<br>28
	Confidence_par	Correlation<br>Coefficient<br>Sig. (2-tailed)<br>N	-0.025<br>0.901<br>28	0.264<br>0.175<br>28	0.240<br>0.219<br>28	0.848**<br>0.000<br>28	0.779**<br>0.000<br>28	1.000<br>.<br>28	0.137<br>0.487<br>28	0.066<br>0.738<br>28	-0.167<br>0.397<br>28
	Correctness_val	Correlation<br>Coefficient<br>Sig. (2-tailed)<br>N	0.136<br>0.490<br>28	0.005<br>0.981<br>28	-0.036<br>0.855<br>28	0.284<br>0.143<br>28	0.012<br>0.950<br>28	0.137<br>0.487<br>28	1.000<br>.<br>28	-0.555**<br>0.002<br>28	0.306<br>0.114<br>28
	Correctness_in<br>v	Correlation<br>Coefficient<br>Sig. (2-tailed)<br>N	0.007<br>0.973<br>28	0.195<br>0.321<br>28	0.167<br>0.395<br>28	0.052<br>0.791<br>28	0.290<br>0.135<br>28	0.066<br>0.738<br>28	-0.555**<br>0.002<br>28	1.000<br>.<br>28	-0.435*<br>0.021<br>28
	Correctness_pa<br>r	Correlation<br>Coefficient<br>Sig. (2-tailed)<br>N	0.035<br>0.861<br>28	-0.104<br>0.597<br>28	-0.069<br>0.729<br>28	-0.099<br>0.615<br>28	-0.111<br>0.575<br>28	-0.167<br>0.397<br>28	0.306<br>0.114<br>28	-0.435*<br>0.021<br>28	1.000<br>.<br>28

*. Correlation is significant at the 0.05 level (2-tailed).

**. Correlation is significant at the 0.01 level (2-tailed).

Table S24: Correlations of Behavioral Data (% Degree of Confidence, DoC and % Correctness) with HFD (Spearman's rho, Nonparametric), AN patients.

Correlations

			mean_valid_higuchi	mean_invalid_higuchi	mean_paradox_higuchi	Confidence_val	Confidence_inv	Confidence_par	Correctness_val	Correctness_inv	Correctness_par
Spearhman's rho	mean_valid_hig<br>uchi	Correlation<br>Coefficient<br>Sig. (2-tailed)<br>N	1.000<br>.<br>12	0.678*<br>0.015<br>12	0.706*<br>0.010<br>12	-0.126<br>0.697<br>12	-0.084<br>0.795<br>12	-0.099<br>0.760<br>12	0.244<br>0.445<br>12	-0.560<br>0.058<br>12	0.344<br>0.274<br>12
	mean_invalid_h<br>iguchi	Correlation<br>Coefficient<br>Sig. (2-tailed)<br>N	0.678*<br>0.015<br>12	1.000<br>.<br>12	0.881**<br>0.000<br>12	0.308<br>0.331<br>12	0.322<br>0.308<br>12	0.331<br>0.293<br>12	0.442<br>0.151<br>12	-0.190<br>0.554<br>12	0.102<br>0.753<br>12
	mean_paradox_<br>higuchi	Correlation<br>Coefficient<br>Sig. (2-tailed)<br>N	0.706*<br>0.010<br>12	0.881**<br>0.000<br>12	1.000<br>.<br>12	0.343<br>0.276<br>12	0.259<br>0.417<br>12	0.352<br>0.262<br>12	0.339<br>0.281<br>12	-0.208<br>0.517<br>12	0.130<br>0.688<br>12
	Confidence_val	Correlation<br>Coefficient<br>Sig. (2-tailed)<br>N	-0.126<br>0.697<br>12	0.308<br>0.331<br>12	0.343<br>0.276<br>12	1.000<br>.<br>12	0.797**<br>0.002<br>12	0.986**<br>0.000<br>12	0.629*<br>0.028<br>12	0.127<br>0.695<br>12	0.109<br>0.736<br>12
	Confidence_inv	Correlation<br>Coefficient<br>Sig. (2-tailed)<br>N	-0.084<br>0.795<br>12	0.322<br>0.308<br>12	0.259<br>0.417<br>12	0.797 **<br>0.002<br>12	1.000<br>.<br>12	0.782**<br>0.003<br>12	0.459<br>0.133<br>12	0.222<br>0.488<br>12	0.077<br>0.812<br>12
	Confidence_par	Correlation<br>Coefficient<br>Sig. (2-tailed)<br>N	-0.099<br>0.760<br>12	0.331<br>0.293<br>12	0.352<br>0.262<br>12	0.986 **<br>0.000<br>12	0.782**<br>0.003<br>12	1.000<br>.<br>12	0.680*<br>0.015<br>12	0.096<br>0.767<br>12	0.205<br>0.523<br>12
	Correctness_val	Correlation<br>Coefficient<br>Sig. (2-tailed)<br>N	0.244<br>0.445<br>12	0.442<br>0.151<br>12	0.339<br>0.281<br>12	0.629*<br>0.028<br>12	0.459<br>0.133<br>12	0.680*<br>0.015<br>12	1.000<br>.<br>12	-0.203<br>0.527<br>12	0.473<br>0.120<br>12
	Correctness_in<br>v	Correlation<br>Coefficient<br>Sig. (2-tailed)<br>N	-0.560<br>0.058<br>12	-0.190<br>0.554<br>12	-0.208<br>0.517<br>12	0.127<br>0.695<br>12	0.222<br>0.488<br>12	0.096<br>0.767<br>12	-0.203<br>0.527<br>12	1.000<br>.<br>12	0.035<br>0.913<br>12
	Correctness_pa<br>r	Correlation<br>Coefficient<br>Sig. (2-tailed)<br>N	0.344<br>0.274<br>12	0.102<br>0.753<br>12	0.130<br>0.688<br>12	0.109<br>0.736<br>12	0.077<br>0.812<br>12	0.205<br>0.523<br>12	0.473<br>0.120<br>12	0.035<br>0.913<br>12	1.000<br>.<br>12

**. Correlation is significant at the 0.01 level (2-tailed).

*. Correlation is significant at the 0.05 level (2-tailed).

Table S25: Independent samples t-test of % DoC and % Correctness.

Variable	t	df	p-value
% DoC_valid	1.615	38	0.057<br>partial<br>(0.115/2)

% Correctness_Valid	1.595	38	0.059 Partial (0.119/2)

Table S26: Kruskal-Wallis test for the AppEn, valid syllogism.

Test Statistics[a,b]

	Ch1_valid	Ch2_valid	Ch3_valid	Ch4_valid	Ch5_valid	Ch6_valid	Ch7_valid	Ch8_valid	Ch9_valid	Ch10_valid	Ch11_valid	Ch12_valid	Ch13_valid	Ch14_valid
Chi-Square	1.258	2.447	8.028	3.348	8.028	8.028	6.001	4.268	7.534	9.063	5.031	3.457	4.268	2.178
df	1	1	1	1	1	1	1	1	1	1	1	1	1	1
Asymp. Sig.	0.262	0.118	0.005	0.067	0.005	0.005	0.014	0.039	0.006	0.003	0.025	0.063	0.039	0.140

a. Kruskal Wallis Test
b. Grouping Variable: group

Table S27: Kruskal-Wallis test for AppEn, the invalid syllogism.

Test Statistics[a,b]

	Ch1_invalid	Ch2_invalid	Ch3_invalid	Ch4_invalid	Ch5_invalid	Ch6_invalid	Ch7_invalid	Ch8_invalid	Ch9_invalid	Ch10_invalid	Ch11_invalid	Ch12_invalid	Ch13_invalid	Ch14_invalid
Chi-Square	9.422	9.973	11.123	10.540	17.073	17.564	13.829	11.520	10.540	13.829	8.711	8.711	12.129	6.900
df	1	1	1	1	1	1	1	1	1	1	1	1	1	1
Asymp. Sig.	0.002	0.002	0.001	0.001	0.000	0.000	0.000	0.001	0.001	0.000	0.003	0.003	0.000	0.009

a. Kruskal Wallis Test
b. Grouping Variable: group

Table S28: Kruskal-Wallis test for the AppEn, paradox syllogism.

Test Statistics[a,b]

	Ch1_paradox	Ch2_paradox	Ch3_paradox	Ch4_paradox	Ch5_paradox	Ch6_paradox	Ch7_paradox	Ch8_paradox	Ch9_paradox	Ch10_paradox	Ch11_paradox	Ch12_paradox	Ch13_paradox	Ch14_paradox
Chi-Square	3.136	2.635	3.348	7.056	6.746	4.516	5.715	7.373	8.537	8.366	3.794	6.593	5.575	0.223
df	1	1	1	1	1	1	1	1	1	1	1	1	1	1
Asymp. Sig.	0.077	0.105	0.067	0.008	0.009	0.034	0.017	0.007	0.003	0.004	0.051	0.010	0.018	0.637

a. Kruskal Wallis Test
b. Grouping Variable: group

Table S29: Non-parametric, Kruskal-Wallis test for the ***Grand mean values*** of AppEn.

Test Statistics[a,b]

	mean_valid_AE	mean_invalid_AE	mean_paradox _AE
Chi-Square	8.028	15.409	6.900
df	1	1	1
Asymp. Sig.	0.005	0.000	0.009

a. Kruskal Wallis Test

b. Grouping Variable: group

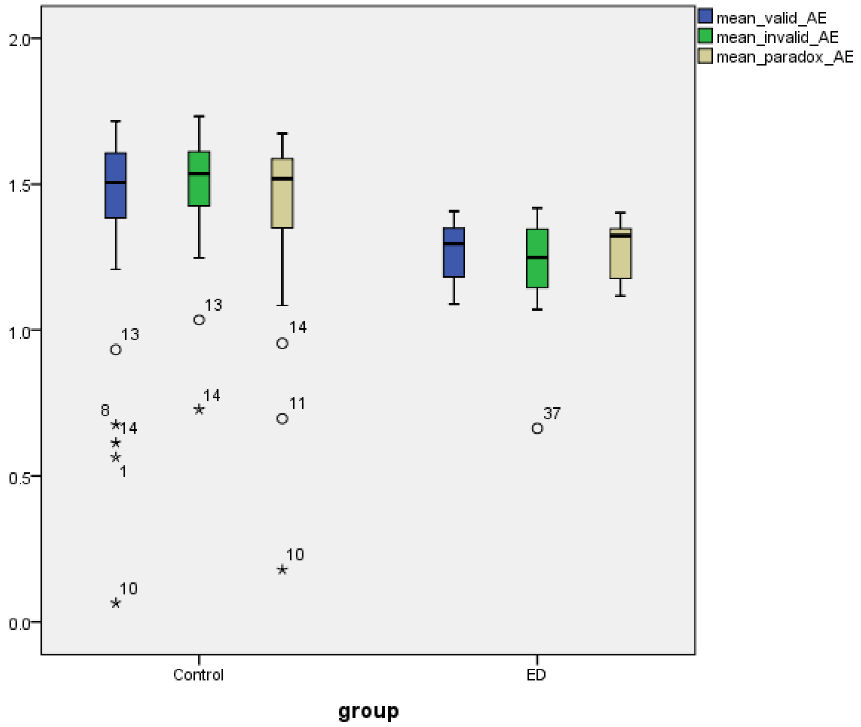

Figure S7: Box plot of Grand mean AppEn values for the two groups, across 14 channels, all syllogisms.

Table S30: Kruskal-Wallis Test of EEG Alpha rhythm, valid syllogism.

Test Statistics[a,b]

	Ch1_valid	Ch2_valid	Ch3_valid	Ch4_valid	Ch5_valid	Ch6_valid	Ch7_valid	Ch8_valid	Ch9_valid	Ch10_valid	Ch11_valid	Ch12_valid	Ch13_valid	Ch14_valid
Chi-Square	0.440	0.408	0.133	0.052	1.462	0.079	0.001	0.284	0.317	3.351	0.079	0.050	0.756	3.641
df	1	1	1	1	1	1	1	1	1	1	1	1	1	1

Asymp. Sig.	0.507	0.523	0.715	0.820	0.227	0.778	0.978	0.594	0.573	0.067	0.779	0.823	0.385	0.056

a. Kruskal Wallis Test
b. Grouping Variable: group

Table S31: Kruskal-Wallis Test of EEG Alpha rhythm, invalid syllogism.

Test Statistics[a,b]

	Ch1_invalid	Ch2_invalid	Ch3_invalid	Ch4_invalid	Ch5_invalid	Ch6_invalid	Ch7_invalid	Ch8_invalid	Ch9_invalid	Ch10_invalid	Ch11_invalid	Ch12_invalid	Ch13_invalid	Ch14_invalid
Chi-Square	0.293	1.899	0.013	2.679	0.016	0.535	0.720	0.231	3.964	2.551	0.079	3.749	1.180	0.064
df	1	1	1	1	1	1	1	1	1	1	1	1	1	1
Asymp. Sig.	0.589	0.168	0.910	0.102	0.899	0.465	0.396	0.631	0.046	0.110	0.778	0.053	0.277	0.800

a. Kruskal Wallis Test
b. Grouping Variable: group

Table S32: Kruskal-Wallis Test of EEG Alpha rhythm, paradox syllogism.

Test Statistics[a,b]

	Ch1_paradox	Ch2_paradox	Ch3_paradox	Ch4_paradox	Ch5_paradox	Ch6_paradox	Ch7_paradox	Ch8_paradox	Ch9_paradox	Ch10_paradox	Ch11_paradox	Ch13_paradox	Ch14_paradox
Chi-Square	0.620	3.999	1.812	0.154	0.781	0.273	0.064	0.096	0.618	3.976	4.420	0.058	4.244
df	1	1	1	1	1	1	1	1	1	1	1	1	1
Asymp. Sig.	0.431	0.046	0.178	0.694	0.377	0.602	0.800	0.757	0.432	0.046	0.036	0.810	0.039

a. Kruskal Wallis Test
b. Grouping Variable: group

Table S33: Kruskal-Wallis Test of EEG Beta rhythm, valid syllogism.

Test Statistics[a,b]

	Ch1_valid	Ch2_valid	Ch3_valid	Ch4_valid	Ch5_valid	Ch6_valid	Ch7_valid	Ch8_valid	Ch9_valid	Ch10_valid	Ch11_valid	Ch12_valid	Ch13_valid	Ch14_valid
Chi-Square	5.375	0.113	7.020	5.860	0.000	0.017	0.409	2.570	0.205	2.081	7.440	0.252	0.276	2.843
df	1	1	1	1	1	1	1	1	1	1	1	1	1	1
Asymp. Sig.	0.020	0.736	0.008	0.015	1.000	0.897	0.522	0.109	0.651	0.149	0.006	0.616	0.600	0.092

a. Kruskal Wallis Test
b. Grouping Variable: group

Table S34: Kruskal-Wallis Test of EEG Beta rhythm, invalid syllogism.

Test Statistics[a,b]

	Ch1_invalid	Ch2_invalid	Ch3_invalid	Ch4_invalid	Ch5_invalid	Ch6_invalid	Ch7_invalid	Ch8_invalid	Ch9_invalid	Ch10_invalid	Ch11_invalid	Ch12_invalid	Ch13_invalid	Ch14_invalid
Chi-Square	5.474	0.149	2.942	0.137	1.032	4.141	0.054	0.993	0.164	1.241	2.177	0.294	2.109	2.637
df	1	1	1	1	1	1	1	1	1	1	1	1	1	1
Asymp. Sig.	0.019	0.700	0.086	0.711	0.310	0.042	0.816	0.319	0.686	0.265	0.140	0.588	0.146	0.104

a. Kruskal Wallis Test
b. Grouping Variable: group

Table S35: Kruskal-Wallis Test of EEG Beta rhythm, paradox syllogism.

Test Statistics[a,b]

	Ch1_paradox	Ch2_paradox	Ch3_paradox	Ch4_paradox	Ch5_paradox	Ch6_paradox	Ch7_paradox	Ch8_paradox	Ch9_paradox	Ch10_paradox	Ch11_paradox	Ch12_paradox	Ch13_paradox	Ch14_paradox

Chi-Square	2.344	0.743	2.451	1.441	1.642	1.238	0.409	0.039	1.636	0.673	0.040	0.338	0.647	0.024
df	1	1	1	1	1	1	1	1	1	1	1	1	1	1
Asymp. Sig.	0.126	0.389	0.117	0.230	0.200	0.266	0.522	0.043	0.201	0.412	0.842	0.561	0.421	0.876

a. Kruskal Wallis Test
b. Grouping Variable: group

Table S36: Kruskal-Wallis Test of EEG Delta rhythm, valid syllogism.

Test Statistics[a,b]

	Ch1_valid	Ch2_valid	Ch3_valid	Ch4_valid	Ch5_valid	Ch6_valid	Ch7_valid	Ch8_valid	Ch9_valid	Ch10_valid	Ch11_valid	Ch12_valid	Ch13_valid	Ch14_valid
Chi-Square	1.133	0.133	0.007	0.000	0.531	0.078	1.256	2.374	0.572	0.064	0.227	0.133	0.113	0.615
df	1	1	1	1	1	1	1	1	1	1	1	1	1	1
Asymp. Sig.	0.287	0.716	0.933	1.000	0.466	0.779	0.262	0.123	0.449	0.801	0.634	0.716	0.737	0.433

a. Kruskal Wallis Test
b. Grouping Variable: group

Table S37: Kruskal-Wallis Test of EEG Delta rhythm, invalid syllogism.

Test Statistics[a,b]

	Ch1_invalid	Ch2_invalid	Ch3_invalid	Ch4_invalid	Ch5_invalid	Ch6_invalid	Ch7_invalid	Ch8_invalid	Ch9_invalid	Ch10_invalid	Ch11_invalid	Ch12_invalid	Ch13_invalid	Ch14_invalid
Chi-Square	0.038	0.346	0.804	0.113	2.826	0.452	0.660	0.227	0.254	0.020	0.154	1.075	0.227	1.590
df	1	1	1	1	1	1	1	1	1	1	1	1	1	1
Asymp. Sig.	0.845	0.556	0.370	0.737	0.093	0.501	0.416	0.634	0.614	0.889	0.695	0.300	0.634	0.207

a. Kruskal Wallis Test
b. Grouping Variable: group

Table S38: Kruskal-Wallis Test of EEG Delta rhythm, paradox syllogism.

Test Statistics[a,b]

	Ch1_paradox	Ch2_paradox	Ch3_paradox	Ch4_paradox	Ch5_paradox	Ch6_paradox	Ch7_paradox	Ch8_paradox	Ch9_paradox	Ch10_paradox	Ch11_paradox	Ch12_paradox	Ch13_paradox	Ch14_paradox
Chi-Square	0.078	1.520	0.095	0.154	6.358	1.451	1.194	0.254	2.042	0.346	0.615	0.415	1.075	0.001
df	1	1	1	1	1	1	1	1	1	1	1	1	1	1
Asymp. Sig.	0.779	0.218	0.758	0.695	0.012	0.228	0.275	0.614	0.153	0.556	0.433	0.519	0.300	0.978

a. Kruskal Wallis Test
b. Grouping Variable: group

Table S39: Kruskal-Wallis Test of EEG Theta rhythm, valid syllogism.

Test Statistics[a,b]

	Ch1_valid	Ch2_valid	Ch3_valid	Ch4_valid	Ch5_valid	Ch6_valid	Ch7_valid	Ch8_valid	Ch9_valid	Ch10_valid	Ch11_valid	Ch12_valid	Ch13_valid	Ch14_valid
Chi-Square	0.234	0.020	0.016	0.021	2.436	0.096	1.112	0.491	0.020	0.013	0.034	0.815	0.002	1.742
df	1	1	1	1	1	1	1	1	1	1	1	1	1	1
Asymp. Sig.	0.628	0.888	0.899	0.885	0.119	0.756	0.292	0.483	0.888	0.911	0.854	0.367	0.966	0.187

a. Kruskal Wallis Test
b. Grouping Variable: group

Table S40: Kruskal-Wallis Test of EEG Theta rhythm, invalid syllogism.

Test Statistics[a,b]

	Ch1_invalid	Ch2_invalid	Ch3_invalid	Ch4_invalid	Ch5_invalid	Ch6_invalid	Ch7_invalid	Ch8_invalid	Ch9_invalid	Ch10_invalid	Ch11_invalid	Ch12_invalid	Ch13_invalid	Ch14_invalid
Chi-Square	0.029	5.103	0.010	0.965	0.758	0.126	2.788	4.087	2.867	0.785	5.699	1.146	3.249	4.034
df	1	1	1	1	1	1	1	1	1	1	1	1	1	1
Asymp. Sig.	0.865	0.024	0.922	0.326	0.384	0.723	0.095	0.043	0.090	0.376	0.017	0.284	0.071	0.045

a. Kruskal Wallis Test
b. Grouping Variable: group

Table S41: Kruskal-Wallis Test of EEG Theta rhythm, paradox syllogism.

Test Statistics[a,b]

	Ch1_paradox	Ch2_paradox	Ch3_paradox	Ch4_paradox	Ch5_paradox	Ch6_paradox	Ch7_paradox	Ch8_paradox	Ch9_paradox	Ch10_paradox	Ch11_paradox	Ch12_paradox	Ch13_paradox	Ch14_paradox
Chi-Square	3.239	0.114	0.711	2.692	0.013	0.001	0.013	0.144	0.064	0.064	0.114	1.069	0.457	0.020
df	1	1	1	1	1	1	1	1	1	1	1	1	1	1
Asymp. Sig.	0.072	0.735	0.399	0.101	0.910	0.978	0.910	0.704	0.800	0.800	0.735	0.301	0.499	0.887

a. Kruskal Wallis Test
b. Grouping Variable: group
